# Re-Discovery of *Giardiavirus*: Genomic and Functional Analysis of Viruses from *Giardia duodenalis* Isolates

**DOI:** 10.3390/biomedicines9060654

**Published:** 2021-06-08

**Authors:** Gianluca Marucci, Ilaria Zullino, Lucia Bertuccini, Serena Camerini, Serena Cecchetti, Agostina Pietrantoni, Marialuisa Casella, Paolo Vatta, Alex D. Greenwood, Annarita Fiorillo, Marco Lalle

**Affiliations:** 1 Unit of Foodborne and Neglected Parasitic Disease, Department of Infectious Diseases, Istituto Superiore di Sanità, Viale Regina Elena 299, 00161 Rome, Italy; gianluca.marucci@iss.it (G.M.); ilariazullino14@gmail.com (I.Z.); paolo.vatta@iss.it (P.V.); 2 Core Facilities, Istituto Superiore di Sanità, Viale Regina Elena 299, 00161 Rome, Italy; lucia.bertuccini@iss.it (L.B.); serena.camerini@iss.it (S.C.); serena.cecchetti@iss.it (S.C.); agostina.pietrantoni@iss.it (A.P.); marialuisa.casella@iss.it (M.C.); 3 Leibniz Institute for Zoo and Wildlife Research, 10315 Berlin, Germany; greenwood@izw-berlin.de; 4 Department of Veterinary Medicine, Freie Universität Berlin, 14195 Berlin, Germany; 5 Department of Biochemical Science “A. Rossi-Fanelli”, Sapienza University, 00185 Rome, Italy; annarita.fiorillo@uniroma1.it

**Keywords:** *Giardia duodenalis*, *Giardiavirus*, RNA viruses, virion, genomics, proteomics

## Abstract

Giardiasis, caused by the protozoan parasite *Giardia duodenalis*, is an intestinal diarrheal disease affecting almost one billion people worldwide. A small endosymbiotic dsRNA viruses, *G. lamblia* virus (GLV), genus *Giardiavirus*, family *Totiviridae*, might inhabit human and animal isolates of *G. duodenalis*. Three GLV genomes have been sequenced so far, and only one was intensively studied; moreover, a positive correlation between GLV and parasite virulence is yet to be proved. To understand the biological significance of GLV infection in *Giardia*, the characterization of several GLV strains from naturally infected *G. duodenalis* isolates is necessary. Here we report high-throughput sequencing of four GLVs strains, from *Giardia* isolates of human and animal origin. We also report on a new, unclassified viral sequence (designed GdRV-2), unrelated to *Giardiavirus*, encoding and expressing for a single large protein with an RdRp domain homologous to *Totiviridae* and *Botybirnaviridae*. The result of our sequencing and proteomic analyses challenge the current knowledge on GLV and strongly suggest that viral capsid protein translation unusually starts with a proline and that translation of the RNA-dependent RNA polymerase (RdRp) occurs via a +1/−2 ribosomal frameshift mechanism. Nucleotide polymorphism, confirmed by mass-spectrometry analysis, was also observed among and between GLV strains. Phylogenetic analysis indicated the occurrence of at least two GLV subtypes which display different phenotypes and transmissibility in experimental infections of a GLV naïve *Giardia* isolate.

## 1. Introduction

*Giardia duodenalis* (syn. *G. lamblia* and *G. intestinalis*) is a flagellated protozoan parasite pathogenic for humans and various mammals, including pets and livestock. It is responsible for giardiasis, the most common non-viral/non-bacterial diarrheal disease worldwide [1]. Humans infection is almost exclusively associated with two genetic groups, namely Assemblage A and B, whereas more host-specific Assemblages (C–H) are responsible for giardiasis in animals; even if zoonotic potential has been recently recognized also for Assemblage A and B [2]. Children and young animals are commonly more susceptible to *G. duodenalis* infection, likely related to hygiene and immunocompetence [1]. Although the infection is asymptomatic in the majority of human and animal cases and symptoms are self-limited in acute infection, giardiasis may occur as a chronic disease or give rise to post-infectious sequelae, such as irritable bowel syndrome [3]. Several attempts to associate giardiasis outcomes, especially in humans, with infection with either one or the other *G. duodenalis* assemblages remain inconclusive. Similarly, occurrence of clinical cases refractory to antigiardial-drug treatment, i.e., metronidazole, or the selection of tolerant *G. duodenalis* isolates are not clearly associated with acquired genetic traits [2,3]. However, an interplay between *G. duodenalis* and the gut microflora has been implicated in several aspect of giardiasis (e.g., *G. duodenalis* colonization of the gut and host immune modulation) [4].

Evidences have linked the severity of disease caused by some protozoan parasites with the presence of viral endosymbionts in the parasite cytoplasm. Two examples are represented by the relationship between *Leishmania* spp. and *Trichomonas vaginalis* with their respective double-strand (ds) RNA virus endosymbionts, *Leishmaniavirus* (LRV) and *Trichomonasvirus* (TVV), and their effect on the human host [5]. Although LRV does not directly affect *Leishmania* growth, the release of the viral dsRNA from degenerated LRV subverts the human innate immune response by inducing hyper-inflammation via the activation of the host Toll-like receptor 3 (TLR3) signaling, thus increasing parasite burden, exacerbating the disease and leading to larger lesions at the peak of infection [6]. Similarly, the presence of TVV can also modulate the human immune response to *T. vaginalis* infection increasing colonization of vaginal pathogenic bacteria associated with vaginosis [7]. Additionally, TVV also impairs parasite growth and alters the parasite proteome promoting its virulence [8].

*G. duodenalis* can be infected with the *Giardia lamblia virus* (GLV) [5]. GLV was first identified, about 35 years ago, in a *G. duodenalis* isolate from a human patient (HP-1, Human Portland-1) [9,10]. GLV is a small non-enveloped virus with a non-segmented dsRNA genome and it is the only recognized species of the genus *Giardiavirus,* family *Totiviridae*. *Totiviridae* also include other four officially recognized genera, *Leishmaniavirus* and *Trichomonasvirus,* which exclusively infect protozoa with host-specificity, and *Totivirus,* and *Victorivirus,* which infect fungi [5]. Although many unclassified *Totiviridae* have been so far reported, belonging to the genera *Artivirus* which infect arthropod or fish and *Insevirus,* with insect hosts, it has recently been proposed that they can be included in the same clade with *Giardiavirus* [11,12,13].

The genome of the GLV strain from *G. duodenalis* HP-1 isolate was fully sequenced in the 1990s (GenBank ID L13218.1) [10]. The GLV genome (≈6.3 Kb) contains two partially overlapping ORFs that encode for the capsid protein (CP, ORF1) and for the RNA-dependent RNA polymerase (RdRp, ORF2), respectively [10]. *Giardiavirus* mRNA lacks a conventional 7-methyl-G(5′)ppp(5′)G capping, and CP translation depends on an internal ribosomal entry site (IRES), a cis-acting RNA region that promotes internal initiation of protein synthesis, using cap-independent mechanisms [14]. The minimal GLV IRES (nucleotides 114–631) encompass 367 nt of the 5′ untranslated region (5′-UTR) and a 264 nt stretch of the downstream CP coding sequence (ATG position at nt 367–369 according to L13218.1) [15,16,17,18,19,20]. The RdRp is instead exclusively expressed, with low efficiency, as CP/RdRp fusion protein by means of a -1 programmed ribosomal frameshift (PRF) occurring during the translation process [21]. An early report suggests that the 100 kDa CP undergoes a post-translational maturation process with the removal of the first 32 N-terminal residues by an unidentified *G. duodenalis* cysteine protease [22]. The GLV viral capsid (diameter 48.5 nm) displays an icosahedral structure built by 120 CP subunits (with 1 or 2 CP/RdRp per virion) in a T = 2 lattice [23]. GLV particle has a great stability, it is easily purified intact (i.e., containing the dsRNA genome) and is more thermostable than other *Totiviridae* virions, providing explanation for the ability of GLV to be transmitted extracellularly, a unique feature among *Totiviridae* infecting protozoan and fungi [23]. This is consistent with (i) the phylogenetic relationship of GLV with *Totiviridae*-like viruses isolated from arthropods and bony fishes that are also able to be transmitted by extracellular means [23,24]; and (ii) the observation that GLV, released and purified from trophozoites culture media, can infect naïve *G. duodenalis* isolates of Assemblage A and B [25]. Regardless of the Assemblage, not all *Giardia* isolates seem to be susceptible to GLV infection suggesting the virus might enter the parasite via endocytosis (endocytosis blocking agents inhibits GLV infection) mediated by a not-yet-identified receptor [26,27].

Despite how up to 30% of *G. duodenalis* isolates have been found to be positive when tested for GLV [28,29,30,31], only three full-length viral genomes have been deposited in GenBank, two from *G. duodenalis* human isolates and one from a dog isolate [32,33]. A comparison of these three GLV sequences provided evidence only for a limited variability at both the nucleotide and protein level [32,33].

In order to expand the current knowledge on GLV, we applied a high-throughput sequencing of viral genomes from different *G. duodenalis* isolates (having different host origins and belonging to zoonotic and host-specific assemblages) and an integrated mass-spectrometry-based proteome analysis of the viral proteins. We also investigated biological properties of the identified viral strains, both in their original *G. duodenalis* isolate and by experimental infection of a naïve *G. duodenalis* isolate.

## 2. Materials and Methods

### 2.1. Parasite Culture

*G. duodenalis* isolates used in this work are listed in Table 1. Trophozoites were axenically grown at 37 °C in 10 mL screw-cap tubes (Nunc^TM^, Thermo Fisher Scientific, Waltham, MA, USA) filled with TYI-S33 medium, supplemented with 10% adult bovine serum (Euroclone S.p.A., Milan, Italy) and bovine bile (Sigma-Aldrich, Merck Life Science S.r.l., Milan, Italy) [34] and sub-cultured into fresh medium when confluence was reached. Where appropriate, parasites were grown in a 50 or 500 mL screw-cap culture flask (Nunc^TM^, Thermo Fisher Scientific, Waltham, MA, USA).

### 2.2. Viral dsRNA Isolation and RNAseq Experiments

Total nucleic acids (DNA and RNA) were extracted from ~1 × 10^7^ GLV-infected trophozoites, using the hot phenol protocol. Briefly, parasites were detached from culture tube by chilling on ice and cells harvested by centrifugation (900× *g*, 10 min, 4 °C), cell pellet was washed twice with cold PBS, resuspended in 500 µL of buffer (0.1 M Na-acetate pH 5.0/sodium dodecyl-sulfate 1%) and mixed quickly. The lysate was extracted twice with hot (70 °C) water saturated phenol, followed by one extraction with phenol/chloroform/isoamylalcohol (25:24:1) and one with chloroform/isoamylalcohol (24:1). Finally, nucleic acids were precipitated for 1 h, at −20 °C, by addition of 1:10 vol. of 3 M Na-acetate, pH 5.0, and 2.5 vol. of ice cold 96% EtOH 2.5 vol, pellet washed once with 70% EtOH and finally resuspended in 50 μL of molecular grade H_2_O. The band corresponding to the viral dsRNA (migrating as satellite band of ~7.0 Kb below the band of *G. duodenalis* genomic DNA) was separated on 0.8% agarose gels (run on 1X MOPS buffer), purified from gel by using the QIAquick gel extraction kit (Qiagen, Italy), resuspended in 30–50 μL of molecular grade H_2_O and stored at −80 °C until use. Purified RNA was used for RNAseq experiments with Illumina technology (Biodiversa srl, Rovereto, Italy). Viral RNA (>10 ng) was denaturated at 95 °C for 5 min, quick frozen and used for library preparation with NEBNext^®^ Ultra™ RNA Library Prep Kit for Illumina (New England Biolabs, Évry-Courcouronnes, France) according to manufacturer’s instruction. Illumina libraries were sequenced on an Illumina HiSeq 4000 platform. RNAseq statistics are reported (Supplementary Materials Table S1). Raw sequencing data (fastq file format) are available from the SRA database, under the BioProject accession number PRJNA720885 (27 April 2021).

### 2.3. RNA Extraction, Reverse-Transcription (RT)-PCR and Sequencing

For RT-PCR experiments, total RNA was extracted from ~1 × 10^7^ trophozoites, using the RNAeasy mini kit (Qiagen, Hilden, Germany), according to the manufacturer. One to two hundred ng of total RNA was used in 50 μL reaction containing 50 pmol of each primer (Supplementary Materials Table S2), 10 μL of 5X OneStep buffer (Qiagen, Hilden, Germany), 2 μL of dNTP mix (10 mM each) and 2 μL of OneStep RT-PCR Enzyme Mix (Qiagen, Hilden, Germany). RT-PCR amplifications were performed in a Biometra T-Personal thermocycler (Analytik Jena AG, Jena, Germany). Reverse-transcription step was performed at 53 °C, for 1 h, followed by an inactivation/activation step at 95 °C for 15 min, according to manufacturer. PCR protocol consisted of 40 cycles each of 30 s at 95 °C; 30 s at 53 °C; 1 min/kbp at 68 °C, ended with a 10 min. extension step at 68 °C. Nucleotide sequence of the PCR products was obtained following standard Sanger sequencing. Sequences of all viral genomes were confirmed by RT-PCR amplification of partially overlapping fragments and Sanger sequencing (Supplementary Materials Table S2) and nucleotide gaps, eventually present in contigs, filled. Sequences were deposited in GenBank, under accession numbers from MW659703 to MW659707(25 February 2021).

### 2.4. Vectors’ Construction, Expression and Purification of the Recombinant Protein

Standard procedures were used for plasmid purification and nucleic acid enzymatic restriction. Two portions of the GLV capsid protein were selected for antigen production: an 822 bp fragment (nt 460–1281, from reference L13218.1), encoding for a 274 amino acids N-terminal portion (Ala32-Ser305), and a 250 bp fragment (nt 2294–3250), encoding for a 85 amino acids C-terminal portion. RT-PCR amplification was performed by using the primer pairs GLV-CP-NT_F/GLV-CP-NT_R and GLV-CP-CT_ F/GLV-CP-CT_R (Supplementary Materials Table S3), as detailed before (Tm = 55 °C), using as template total RNA extracted from the *G. duodenalis* HP isolate. To produce an antigen specific for the J17/10 contig, a 258 nt fragment, encoding for an 86 amino acids N-terminal portion of the single ORF, was selected and RT-PCR amplified with the primer pair JA_B-NT_F/JA_B-NT_R, using as template total RNA extracted from the *G. duodenalis* J17/10 isolate. The GLV capsid N-terminal fragment was digested with EcoRI/BamHI and cloned in EcoRI/BamHI-linearized pQE30 vector (Qiagen, Germany), transformed *E. coli M15* strain to express a 6xHIS-tagged fusion protein. GLV capsid C-terminal fragment and the J17/10_B ORF1 N-terminal fragment were digested with PstI/BamHI, cloned in PstI/BamHI-linearized pGEX6P1 vector (GE Healthcare, Chicago, IL, USA) and transformed in *E. coli* XL1Blue strain. Expression of recombinant proteins and purification under native conditions either by glutathione or metal affinity chromatography were performed as previously described [38].

### 2.5. Production of Polyclonal Antibodies

Two BALB/c mice (Charles River Laboratories International, Inc., Wilmington, MA, USA) were immunized intraperitoneally at days 0, 21 and 42 with 150 μL of phosphate-buffered saline (PBS) containing 50 μg of 6xHIS or GST fusion protein. Immunization protocol was applied as previously described [38].

### 2.6. Virus Purification

Viral particles were isolated and purified from the culture medium of infected *G. duodenalis* trophozoites as previously described [29]. Briefly, parasites were grown in TripleFlask (Nunc, Thermo Fisher Scientific, Waltham, MA, USA) with 500 mL of TYI-S33 medium for 72 h. Medium was collected and unattached trophozoites, and cell debris were pelleted by centrifugation (2500× *g*, 10 min, 4 °C). Medium supernatant was then filter-sterilized (0.22 μM) and viral particles were pelleted by ultracentrifugation (143,000× *g*, 2 h, 4 °C), using Optima L100XP Ultracentrifuge (Beckman Coulter SRL, Milan, Italy) and the Ti45 rotor (Beckman Coulter SRL, Milan, Italy). Pellet containing the viral particles was washed with sterile cold PBS and ultracentrifuged (143,000× *g*, 2 h, 4 °C) twice. Viral pellets were carefully resuspended in 10 mL of cold PBS and ClCs powder (Sigma-Aldrich, Merck Life Science S.r.l., Milan, Italy) was add to a final density of 1.39 g/mL (approximately 5.3 g). Volume was adjusted to 12 mL with PBS/CsCl at equivalent density and virions were banded by density gradient centrifugation at 152,000× *g* for 16 h at 4 °C in SW41 rotor (Beckman Coulter SRL, Milan, Italy). The gradient was then fractionated by withdrawing 500 μL aliquots from the top of the gradient. The virion positive fractions were identified by phenol extraction of nucleic acids from 50 μL of each fraction and visualization by gel electrophoresis [29]. Positive fractions were pooled and dialyzed o.n. in sterile PBS/glycerol 20%. Finally, solution was adjusted to 50% glycerol, filter sterilized and aliquot of the preparation stored at −80 °C until use. Concentration of intact virions (virions containing the dsRNA genome) was estimated by quantification of phenol extracted dsRNA assuming the viral genome to be 6300 Kb (6 ng viral RNA = 1 × 10^9^ virions). To check overall quality of the preparation, purified virions were negative stained with ammonium molybdate 4% (pH 7) and examined by transmission electron microscopy (TEM) as previously described [39].

### 2.7. G. duodenalis Experimental Infection with GLV

Logarithmically growing trophozoites were collected, counted in hemocytometer and inoculated in 10 mL screw-cap tubes at 2 × 10^4^ parasites/mL, in the presence of GLV particles at a parasite-to-virus ratio of 1:10,000 (intact virions). Cultures were grown undisturbed at 37 °C for 72 h. After the incubation time, medium was discarded, and attached parasites were washed twice with fresh medium (pre-warmed at 37 °C) and resuspended in 10 mL fresh medium. Parasite culture was diluted 1:10 and growth at 37 °C until confluence (48 h). After three passages in culture, establishment of the virus infection was verified by IF analysis and RNA isolation as previously described. Persistence of viral infection was routinely checked.

### 2.8. G. duodenalis Protein Extracts

For protein extracts, trophozoites (~2 × 10^6^) were collected as detailed above, and, after PBS washing, cell pellet was resuspended in 100 μL of PBS/1% Triton-X100, supplemented with protease/phosphatase inhibitor cocktail (Halt^TM^, Themo Fisher Scientific, Waltham, MA, USA) for 1 h an in ice bath. The lysate was centrifuged at 13,000× *g* for 15 min at 4 °C, and the supernatant was collected. Protein concentration was determined by Bradford assay (Pierce, Rockford, IL, USA), and samples were stored at −70 °C.

### 2.9. Western Blot Analysis

NuPAGE gels (Novex, Invitrogen, Carlsbad, CA, USA) at appropriate concentrations were used for proteins separation as described [40,41]. Filters were probed with the following antibodies at reported dilution: mouse polyclonal anti-GLV-capsid protein_N-terminal (GLV-CP_NT) 1:2000; mouse polyclonal anti-GLV-capsid protein_C-terminal (GLV-CP_CT) 1:2000; mouse polyclonal anti-J17/10_B-ORF1 N-terminal (J_B-ORF1_NT) 1:2000; mouse anti-αTubulin (clone B-5-1-2, Sigma-Aldrich, Merck Life Science S.r.l., Milan, Italy), 1:10,000; rabbit polyclonal N14 (anti-g14-3-3) [40] 1:5000. Interaction was revealed by incubation with HRP-conjugated secondary Ab (Bio-Rad, Hercules, CA USA) at 1:2000–1:3000 dilution, followed by chemiluminescence (Millipore, Merck Life Science S.r.l., Milan, Italy).

### 2.10. Confocal Laser Scanning Microscopy (CLSM)

CLSM analyses of *G. duodenalis* trophozoites and encysting parasites were performed on a Leica TCS SP2 AOBS apparatus (Leica Microsystems, Wetzlar, Germany), as previously described [41]. The following antibodies were used: mouse polyclonal anti-GLV-capsid protein (GLV-CP) at 1:800 dilution; mouse monoclonal anti dsRNA (clone J2; Scicons, Hungary) at 1:100 dilution; rabbit polyclonal N14 (anti-g14-3-3) antiserum [40] at 1:100 dilution; mouse Cy5-conjugated anti-CWP mAb (Waterborne Inc., New Orleans, LA, USA), at dilutions of 1:20. Alexa-Fluor 647- and 488-conjugated anti-rabbit and anti-mouse secondary Ab (Invitrogen, Thermo Fisher Scientific, Waltham, MA, USA ), were used at 1:500 dilution. After staining, coverslips were extensively rinsed and then mounted, using Vectashield^®^ mounting medium (Vector Laboratories Inc., Burlingame, CA, USA) containing 300 nM of 4′,6-diamidino-2-phenylindole (DAPI). Image deconvolution was performed by using Huygens 19.04 software (Scientific Volume Imaging BV, Hilversum, The Netherlands).

### 2.11. Parasite Growth Curve and Virus Replication Analysis

For this set of experiments only trophozoites attached to the culture tube were used. Parasites were inoculated (1 × 10^5^/mL) in duplicate in 10 mL crew cap tubes (NuncTM, Thermo Fisher Scientific, Waltham, MA, USA ) and incubated at 37 °C. Every 24 h, parasites were detached from the tube by incubation on ice and an aliquot counted in a hemocytometer. Viability was determined by addition of Trypan blue (0.2%). Three independent experiments were performed. To follow virus replication and extracellular release, parasites were collected by centrifugation at 1200× *g* for 20 min. After PBS washing, pellet was normalized based on cell number, subdivided equally and then processed for immunofluorescence analysis, protein or nucleic acid extraction, as detailed above. Culture medium was then centrifuged at 10,000× *g* for 30 min. to remove any cellular debris and further ultracentrifuged at 100,000× *g* for 120 min at 4 °C, using a Beckman TL 100.2 rotor (Beckman-Coulter SRL, Milan, Italy), in an Optima MAX-TL centrifuge, to collect viral particles. The viral particles pellet was resuspended in 100 μL of PBS, equally subdivided and either mixed with Laemmli sample buffer for protein gel electrophoresis and Western blot analysis or used for total nucleic acid extraction by hot-phenol.

### 2.12. Mass Spectrometry Analysis

Purified viral particles or protein extracts from *G. duodenalis* isolates infected with the virus, prepared as described above, were separated on 8% SDS-PAGE in Tris-glycine SDS buffer in duplicate. Separated proteins were either subject to western blot (see above paragraph) with anti-GLV-CP polyclonal serum (1:2000), to identify the CP and the CP/RdRp fusion proteins, or stained with Coomassie (Colloidal Blue Staining kit, Invitrogen, Thermo Fisher Scientific, Waltham, MA, USA). Coomassie stained protein bands corresponding to anti-GLV-CP positive bands were cut and treated with dithiothreitol (DTT) and iodoacetamide (IAM), and, finally, separated digestions were performed by using the following different sequencing-grade enzymes: trypsin, (Promega Corporation, Madison, WI, USA), chymotrypsin, (Roche Diagnostics, Mannheim, Germany), GluC and AspN (Roche Diagnostics, Mannheim, Germany). Propionylation experiments were performed treating the dried gel bands with 10 µL solution containing 100 mM propionic anhydride in 2 M NaCl and 50 mM sodium phosphate buffer at pH 8 for 5 min on ice. Then, 90 µL of 50 mM sodium phosphate buffer was added, and the incubation proceeded for 25 min on ice. The reaction was quenched by adding 50 mM Tris at pH 8.5, and the bands were subjected to 5-min-long washes with Tris for three times and 20-min-long washes with ammonium bicarbonate for other three times, before proceeding with DTT, IAM and trypsin digestion as described before. Mass spectrometry analyses were performed with an Orbitrap Fusion Tribrid (Thermo Fisher Scientific, Waltham, CA, USA) mass spectrometer equipped with an Ultimate 3000 HPLC (Dionex, Thermo Fisher Scientific, Waltham, CA, USA). After protein digestion, the peptides were desalted on a trap column (Acclaim PepMap 100 C18, Thermo Fisher Scientific, Waltham, CA, USA) at a flow rate of 20 μL/min before being separated onto a 20 cm–long silica capillary (Silica Tips FS 360-75-8, New Objective, Littleton, MA, USA), packed in-house with a C18, 5 μm, 100 Å resin (Michrom BioResources, Auburn, CA, USA). A binary system of buffer A (95% water, 5% acetonitrile, and 0.1% formic acid) and buffer B (95% acetonitrile, 5% water, and 0.1% formic acid) was adopted over a 60-min gradient (from 5% to 30% of buffer B in 35 min to 80% in 5 min, followed by washing and conditioning steps) with a flow rate of 250 nL/min. The peptide ionization was performed with a Flex-Spray ion nano-source (Thermo Fisher Scientific, Waltham, CA, USA) held at 1.8 KV and with an inlet capillary at 275 °C. Full-scan MS data were acquired in the Orbitrap at 120 K resolution, in a 350–1550 *m*/*z* window f. Data-dependent MS/MS analysis was performed in the Orbitrap at 30 K resolution, in top speed mode with a 3 s cycle time and dynamic exclusion enabled for 60 s. The most intense precursors were selected through a monoisotopic precursor selection filter and with charge >1, isolated in the quadrupole in 1.6 *m*/*z* window, and fragmented by 30% normalized higher-energy collision dissociation (HCD). Automatic gain control (AGC) targets were 4 × 10^5^ for MS and 5 × 10^4^ for MS/MS, with 50 ms for MS and dynamic max injection time for MS/MS. The mass spectra were processed by using the Proteome Discoverer software version 2.4 (PD, Thermo Fisher Scientific, Waltham, CA, USA). A database including *G. duodenalis* database (*Giardia* WB_DB-43_*G intestinalis* Assemblage AWB containing 9295 sequences downloaded from www.giardiadb.org on 23 January 2019), bovine database (6909 sequences downloaded from UniProtKB/Swiss-Prot on 23 January 2019) and the *Giardiavirus* sequences of CP and CP/RdRp proteins from HP and CAT GLV were used to identify GLV proteins from trypsin digested samples. Semi-tryptic cleavage was considered for peptide identification, and 1% FDR was allowed by Percolator node in PD workflow. The database containing the virus proteins was used in the search of peptides deriving from every enzymatic digestion to perform a multi-consensus view of the proteins coverage; no specific cleavage was selected, and a fixed-value PSM validator node in the PD workflow was used. Precursor and fragment tolerance were set to 10 ppm and 0.02 Da, respectively. The searches included fixed modifications for cysteine carbamidomethylation (+57.021464) and variable modifications for methionine oxidation (+15.9949), and N-acetylation (+42.010565) on protein terminus and N-propionylation on protein and peptide N-terminus (+56.026215). The mass spectrometry proteomics data were deposited to the ProteomeXchange Consortium via the PRIDE (http://www.proteomexchange.org/) partner repository with the dataset identifier PXD025785” (4 May 2021).

### 2.13. Transmission Electron Microscopy (TEM)

*G. duodenalis* trophozoites were fixed in 1% glutaraldehyde and 4% paraformaldehyde in 0.1 M sodium cacodylate buffer, pH 7.2, overnight, at 4 °C, and processed according to Perry and Gilbert with slight modifications [42]. Parasites were washed in cacodylate buffer and post-fixed with 1% OsO4 in 0.1 M sodium cacodylate buffer for 1 h, at RT, treated with 1% tannic acid in 0.05 M cacodylate buffer for 30 min and rinsed in 1% sodium sulfate in 0.05 M cacodylate buffer for 10 min. Post-fixed specimens were washed, dehydrated through a graded series of ethanol solutions (30–100% ethanol) and embedded in Agar 100 (Agar Scientific Ltd., Stansted, Essex, UK). Ultrathin sections, obtained by an UC6 ultramicrotome (Leica Microsystems, Wetzlar, Germany), were stained with uranyl acetate and Reynolds’ lead citrate and examined at 100 kV with a FEI/Philips EM 208S Transmission Electron Microscope equipped with acquisition system/Megaview SIS camera (Olympus, Hamburg, Germany).

### 2.14. Computational Analyses

All bioinformatics analyses were performed by using the CLC Genomic Workbench version 11.0.1 (Qiagen, Hilden, Germany). Paired-end reads obtained from Illumina were quality-checked and trimmed (quality limit 0.05; ambiguous limit 2). The reference mapping versus GLV reference sequence (GenBank accession number L13218.1, 15 October 2018) was conducted according to the following settings: match score 1, mismatch cost 2; linear gap cost selected—insertion cost 3, deletion cost 3, length fraction 0.5, similarity fraction 0.8; auto-detect paired distance selected. Unmapped reads obtained from HP, CAT, P2-MER were re-mapped versus *G. duodenalis* WBC6 genome (Assemblage A; ATCC 50803; GCA_000002435.1 GL2), whereas J17/10 unmapped reads were re-mapped versus *G. duodenalis* P15 genome (Assemblage E; GCA_000182665.1). Reads remained after the second mapping analysis were submitted as query in a Nucleotide Basic Local Alignment Search Tool (BLAST). De novo assembly of Illumina RNAseq datasets of HP, CAT, P2MER and J17/10 was carried out with the following settings: word size = 45, bubble size = 98 and minimum contig length = 500. Obtained contigs were filtered by a minimum consensus length of 1000 bp and listed on reads count. Amino acidic sequences of the CP and CP/RdRp proteins were inferred from viral genomic consensus sequences, using ORFfinder (https://www.ncbi.nlm.nih.gov/orffinder/, accessed on 4 May 2021) setting for ORF starting with ATG and alternative initiation codons. Multiple sequence alignments were performed by Clustal W algorithm included in CLC Workbench 8.0.1 software (Qiagen, Hilden, Germany); GLV genome and protein sequences deposited in GenBank were included in the alignment (accession numbers AF525216.1; DQ238861.1; L13218.1). Phylogenetic analysis was conducted with MEGA X [43], by using the Maximum Likelihood method and Tamura–Nei model [44] with 1000 Bootstrap replication. The percentage of trees in which the associated taxa clustered together are shown next to the branches. Initial tree(s) for the heuristic search were obtained automatically by applying Neighbor-Join and BioNJ algorithms to a matrix of pairwise distances estimated by using the Tamura–Nei model, and then selecting the topology with superior log likelihood value.

The minimum free energy (MFE) folding for the viral RNA putative Internal ribosomal entry site region (IRES) was predicted by RNAlifold tool (http://rna.tbi.univie.ac.at/cgi-bin/RNAWebSuite/RNAalifold.cgi, accessed on 4 May 2021), pseudoknots were predicted by using IPknot web server (http://rtips.dna.bio.keio.ac.jp/ipknot/, accessed on 4 May 2021) and secondary structure visualization by using a force directed graph layout (http://rna.tbi.univie.ac.at/forna/, accessed on 4 May 2021).

Automated protein-structure homology modeling of CP was performed by using Swiss-model server (https://swissmodel.expasy.org/, accessed on 4 May 2021) and, as template, the solved structure (3.4 Å resolution) of the ScV-L-A virus viral particle (pdb 1m1c). PyMol software (https://pymol.org/2/, accessed on 4 May 2021) was used for the rendering and superposition of the 3D structures. Chimera software (https://www.cgl.ucsf.edu/chimera/, accessed on 4 May 2021) was used to fit the CP models with the CryoEM map of GLV-CP at 6 Å (EMDB-5948) [23]

Statistical analysis: Data are expressed as mean ± SEM, using GraphPad Prism 5.0 (GraphPad Software, San Diego, CA, USA) and *p* < 0.05 or less was considered to be significant. The statistical analyses were performed by paired and unpaired Student’s *t*-test.

## 3. Results

### 3.1. Sequencing and Sequence Analysis of Giardiavirus Strains

**Figure 1:**
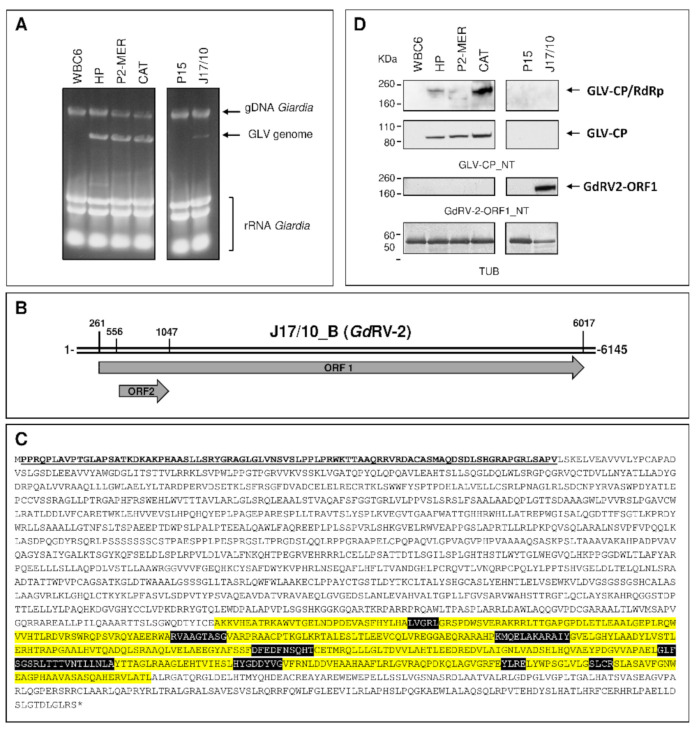
Assessment of virus infection in *G. duodenalis* isolates. (**A**) Ethidium bromide stained agarose gel electrophoresis (0.8%) of the total nucleic acid isolated from *G. duodenalis* isolates infected or not with GLV. The band corresponding to *Giardiavirus* dsRNA is indicated (GLV genome), together with the *G. duodenalis* genomic DNA (gDNA) and ribosomal RNA (rRNA). (**B**) Genomic organization of the J17/10_ B virus-like (GdRV-2) sequence (6145 bp) encoding for ORF1 (frame +3) and a small ORF2 (frame +1) (see Table 2 for details). (**C**) Deduced amino acid sequence of ORF1 encoded by the J17/10_ B virus-like (GdRV-2). Sequence recognized by the mouse GdRV-2-ORF1_N-terminal pAb are in bold and underlined. BlastP detected domains are indicated by color codes. Putative viral RNA-directed RNA-polymerase domain (RdRp, pfam02123) is yellow-boxed. The eight RdRp conserved subdomain (I to VII) are black-boxed [46]. (**D**) Expression of the GLV capsid protein (GLV-CP) and GdRV-2 ORF1 in *G. duodenalis.* Western blot analysis of protein extracts (50 µg) from trophozoites of *G. duodenalis* isolates harboring the viruses (HP, CAT, P2-MER and J17/10) or from control naïve isolates (WBC6 and P15). Immunoblotting was performed with mouse anti-GLV-capsid protein_N-terminal pAb (GLV-CP_NT) and mouse anti-GdRV-2-ORF1 N-terminal (GdRV-2-ORF1_NT) pAb. The anti-α-tubulin (α-α-TUB) was used as loading control. Molecular size markers (kDa) are reported on the left. Arrows indicate the bands corresponding to the GLV-CP, GLV-CP/RdRp and GdRV-2 ORF1. The analysis is representative of three independent experiments.

We collected four different axenized *G. duodenalis* isolates previously reported to be GLV positive (one belonging to the hoofed-specific Assemblage E and three to the zoonotic Assemblage AI) (Table 1), including the human isolate HP, where GLV infection was originally discovered and first GLV sequenced [9,45]. Non-infected controls *G. duodenalis* isolate WB-C6 and P15, for Assemblage A and E, respectively, were included in our study. Unfortunately, no isolates of *G. duodenalis* Assemblage B positive for GLV could be retrieved. The presence of viral RNA in the *G. duodenalis* isolates was initially confirmed by total nucleic acid isolation and gel electrophoresis. As shown (Figure 1), a nucleic acid band of estimated 6.5 Kbp was visible in all four GLV positive *G. duodenalis* isolates. Disappearance of this band following treatment with RNAse-A further confirms the RNA nature (data not shown).

When RNA bands were purified and subjected to RNAseq by Illumina paired-ends sequencing, a total of 60, 57, 53 and 44 millions of reads for HP, P2-MER, CAT and J17/10, respectively, were obtained. The amount of reads mapping on GLV reference sequence (L13218.1) was 86% for CAT, 80% for HP and 76% for P2MER and 0.7% for J17/10 (Supplementary Materials Table S4). The majority of reads unmapped on the GLV reference sequence, mapped either against the *G. duodenalis* WBC6 (97.6% for HP, 77.7% for P2MER, 69.3% for CAT datasets) or the P15 reference genomes (93.4% for J17/10 dataset). Genome sizes of GLV_HP_, GLV_CAT_, GLV_P2MER_ and GLV_J17/10_A_ were comparable with the GLV reference genome (L13218.1) (Table 2). For each viral strain, two main and partially overlapping ORFs were predicted, coding respectively for the capsid protein (CP, ORF1) and the RNA-dependent RNA polymerase (RdRp, ORF2) (Table 2).

**Table 2:** Viral genomes summary.

GLV Strain	Genome Size (nt)	ORF1 (CP) ^b^			ORF1 + ORF2 (CP/RdRp Fusion)			+1/−2 PRF Position (nt)
		Size (nt)	Size (aa)	MW (kDa)	Size (nt)	Size (aa)	MW (kDa)	
L13218.1	6277	2661	886	98.4	5613	1870 ^c^	210.2	NA
CAT	6276	2886 (2796)	961 (931)	106.8 (103.6)	5719 (5629)	1904 (1874)	213.5 (210.3)	3233–3240
HP	6275	2796	931	103.6	5629	1873	210.4	3234–3241
P2-MER	6275	2796	931	103.7	5629	1871	210.3	3234–3241
J17/10_A	6275	2796	931	103.7	5629	1871	210.3	3234–3241
**Unclassified (GdRV-2)**	**Genome size (nt)**	**ORF1**			**ORF2**			
J17/10_B	6145 ^a^	5757	1918	208.3	483	160	17	NA

Abbreviations: NA, not applicable; nt, nucleotide; aa, amino acid; MW, molecular weight; kDa, kilo Dalton; PRF, programmed ribosomal frameshift. ^a^ The 3′- or 5′- ends extension are not confirmed. ^b^ Deduced ORF protein length and protein molecular weight from alternative starting codon are in brackets (see main text for explanation). ^c^ Protein length, including the cleaved N-terminal peptide.

Despite a low degree of divergence (96–99% nucleotide identity), resulting from sequence alignment and cluster analysis (Supplementary Materials Figures S1 and S2), GLV_HP_, GLV_J17/10_A_ and GLV_P2MER_ gather together and in a separate branch respect to GLV_CAT_ that instead clusters with the reference L13218. The same clustering was confirmed when either the CP or the RdRp protein sequences were used for the analysis (Supplementary Materials Figures S2–S4), with all cladogram branch points supported by high bootstrap values. These results point toward the occurrence of two GLV possible “lineages” or strains, of which GLV_HP_ and GLV_CAT_ could be considered prototypes.

To verify the presence of viral RNA sequences other than GLV, de novo assembly of HP, CAT, P2MER and J17/10 datasets was performed. Except those covering the GLV sequence, no contig showing homology (either at nucleotide or amino acid sequence) with other viral-like sequence was found in HP, CAT and P2MER datasets. On the contrary, de novo assembly of J17/10, lead to the identification of an additional long contig, designated as J17/10_B (6145 bp in length). BLAST analysis of contig J17/10_B did not show any homology with known sequences. However, a single large ORF (nt 261–6017), spanning almost the full length of contig J17/10_B, was predicted to code for a ≈200 kDa putative protein (Table 2) having at the C-terminal region (aa 901–1815) homology with viral RNA-directed RNA-polymerase family (pfam 02123) (Figure 1; Supplementary Materials Excel File S1 and Figure S5). This protein family includes RdRPs from ssRNA(+) *Luteovirus* and, dsRNA *Totivirus* and *Rotavirus*. The higher score of homology was indeed found with *totivirus*-like members but also with members of the family *Botybirnaviridae* (Figure 1; Supplementary Materials Excel File S1 and Figure S5), a proposed novel bipartite dsRNA virus family of Fungi with each genome segment in the range of 5.8–6.5 Kbp. [47,48]. A small ORF (nt 565–1047), without an AUG start codon and encoding for a putative 17 kDa protein, with no significant similarity in GenBank, was also detected in the +1 frame (Figure 1 and Table 2). Several attempts to PCR amplify the contig J17/10_B sequence from the genomic DNA of the *G. duodenalis* isolate J17/10, using different primers combination, did not retrieve any result (data not shown), indicating that the sequence is not integrated in the parasite genome. These evidences suggest that *G. duodenalis* isolate J17/10 is infected with both GLV and a new RNA virus we tentatively named GdRV-2 (for *Giardia duodenalis* RNA Virus-2).

To confirm viral proteins expression in the *G. duodenalis* infected trophozoites, immunoblot were performed with in-house produced mouse pAbs raised against the N-terminus of either GLV capsid protein (Supplementary Materials Figure S3) or the GdRV-2 ORF1. As shown (Figure 1), protein bands at the molecular size expected for the GLV-CP (≈100 KDa) and the GLV-CP/RdRp fusion protein (≈200 KDa) were immunodecorated by the anti-GLV-CP_NT pAb in the total protein lysate of the *G. duodenalis* isolates HP, CAT and P2MER. No signal was observed in the uninfected WBC6 and P15 control isolates, as expected. Surprisingly, anti-GLV-CP_NT pAb did not recognize any protein band in the isolate J17/10 (Figure 1). On the contrary, the GdRV-2-ORF1_NT pAb detected a band of approximately 200 KDa exclusively in the J17/10 isolate (Figure 1), thus confirming the expression of the putative ORF1 encoded by the GdRV-2 in the isolate J17/10.

### 3.2. Alternative Capsid Protein Translation Starting Point in the GLV Genomes

Three nucleotide insertions and one deletion were observed in all of our GLV genomes when compared with both the reference (L13218) and the other two available GLV genomes (Supplementary Materials Figure S1), thus leading to alteration of the ORF coding sequences (see below). Heterogeneous nucleotide positions were also detected within GLV_HP_ and GLV_J17/10A_ genome (Supplementary Materials Figure S1 and Table 3), with some potentially resulting in amino acid substitutions (Supplementary Materials Figures S3 and S4 and Table 3).

**Figure 2:**
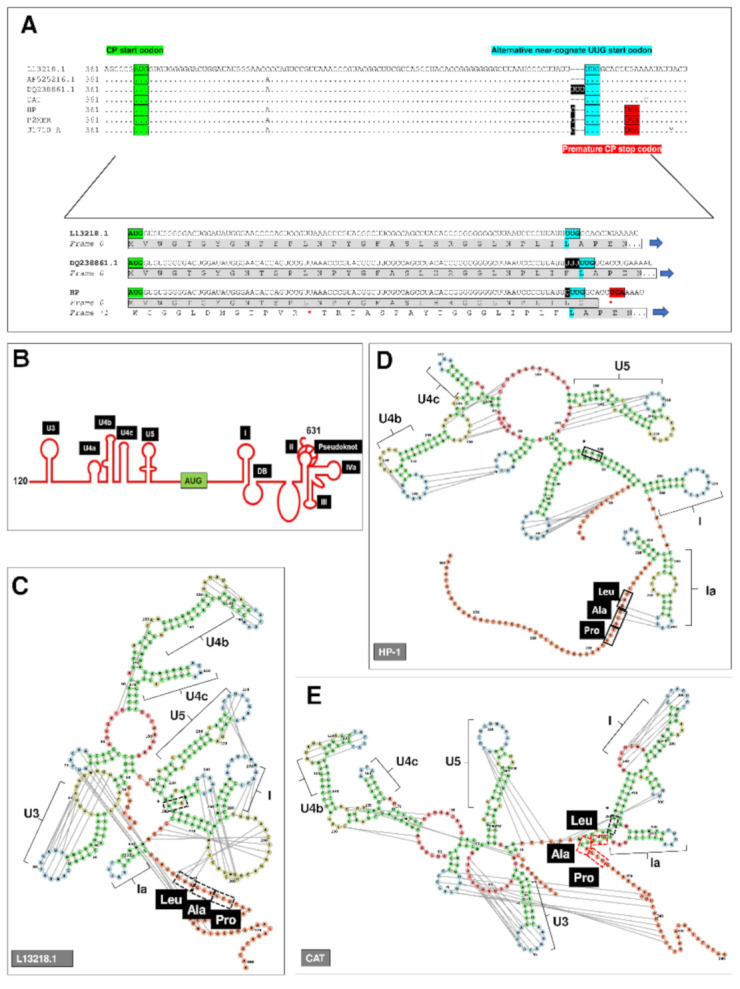
Alternative translational starting site for the GLV capsid protein. (**A**) Upper panel: multiple alignment of the first 114 nt of the GLV-CP ORF. Capsid protein AUG start codon as originally proposed [29] is green boxed. Nucleotide insertion in the GLV genomes are black boxed and the resulting premature stop codon in the capsid protein ORF is red boxed. The alternative near-cognate UUG codon is boxed in light blue. Lower panel: the CP ORF and the corresponding amino acid translation frames for GLV_HP_; DQ238861.1 and the reference L13218.1 are reported. Stop signals in the amino acid sequence are indicated by red asterisks (*). Continuation of translation is indicated by blue arrows. (**B**) Structural model of the IRES in GLV mRNA as originally proposed (adapted from Garlapati and Wang, 2004) [17]. The U3 pseudoknot (nt 134–176) and the upstream stem-loops U4a (nt 204–219), U4b (nt 221–261), U4c (nt 263–292) and U5 (nt 314–344) in the 5′-UTR of the viral genome, together with the stem-loop I (nt 378–402), II, III, Iva, a downstream box (DB) sequence (nt 433–445), and another pseudoknot (nt 511–587) in the CP coding region are depicted and indicated. (**C**–**E**) Visualization, using a force directed graph layout, of the reference L13218.1 (**B**), HP (**C**) and CAT (**D**) putative *Giardiavirus* IRES (nt 120–501) secondary structures (see Materials and Methods). Dashed black box with asterisk, the originally proposed AUG start codon; dashed red boxes, triplets encoding the newly proposed non-AUG cognate start codon and the following two codons (encoding for Leucine, Alanine and Proline respectively). The U3 pseudoknot and the upstream stem-loops U4b, U4c and U5 in the 5′-UTR of the viral genome, together with the (I) and a newly detected (Ia) stem-loops and indicated. Pseudoknots are indicated by gray lines.

A cytosine insertion, with the exception of the GLV_CAT_ strain, was found 89 nucleotides downstream the originally proposed AUG start codon for the *cp* gene (nt 373–375, according to HP sequence) [29] thus leading to a premature stop in the CP ORF (Figure 2 and Supplementary Materials Figures S3 and S4). Indeed, the CP ORF could be preserved in GLV_HP_, GLV_J17/10_A_ and GLV_P2MER_ admitting translation starts from a downstream near-cognate UUG codon (nt 464–466, according to HP sequence), coding for Leu, or from the following non-AUG codons (nt 467–469; 470–472), coding for Ala and Pro, respectively. This is in accordance with bands comparable molecular size (≈100 KDa) immunostained by the anti-GLV-CP_NT pAb in GLV-infected *G. duodenalis* isolates HP, CAT and P2MER (Figure 1). It is well-known that several viral proteins are exclusively translated from a near-cognate or non-AUG start codons by means of unconventional translation regulatory mechanisms [49,50,51], such as IRES.

To support our hypothesis on the occurrence of an alternative translational starting site, we first attempted to predict the conservation of the previously identified IRES structures [15,17,19] in the RNA sequences of GLV_HP_ and GLV_CAT_, as well as in reference L13218.1. Prediction was performed by using the sequence from nt 120 to nt 501, that includes the newly suggested starting codon and 36 nts downstream the CP coding sequence (Figure 2 and Supplementary Materials Figure S6). Most of the relevant IRES secondary structures previously reported [14,15,16,17,18,19] were confirmed, including the pseudoknot U3 (but not in GLV_HP_) and the stem-loops U4b, U4c, U5 and I (Figure 2 and Supplementary Materials Figure S6). Noteworthy, we found two main differences with the originally reported IRES structure. First, the originally CP translation start codon results always embedded in a stem loop structure, thus making unlikely the translation from the original proposed AUG start codon, even in GLV_CAT_ and L13218.1 genome where no ORF interruption occurs (Figure 2 and Supplementary Materials Figure S6). Second, instead of a downstream box (DB) sequence, a stem-loop, here termed Ia, was predicted just upstream (GLV_HP_ and L13218.1) or embedding (GLV_CAT_) the near-cognate UUG codon (nt 464–466, according to HP sequence) (Figure 2). In particular, in GLV_HP_ and L13218.1 models, the stem-loop Ia might form pseudoknots that involve the nucleotides of the near-cognate UUG codon. In both GLV_CAT_ and GLV_HP_ models, these observations prompt for translation starting at codon 470–472 (nt position according to GLV_HP_ sequence) coding for proline and not at the near-cognate UUG codon (nt 464–466) for leucine.

In the attempt to unravel the question, mass spectrometry analysis was conducted by using GLV_HP_ and GLV_CAT_ viral particles purified from spent culture medium and total protein extracts from trophozoites of *G. duodenalis* isolates HP, CAT and P2MER. Almost 90% of the CP protein sequences of both GLV_HP_ and GLV_CAT_ were mapped by mass spectrometry analysis (Supplementary Materials Figure S7). No peptide covering the predicted first N-terminal 32 aa of the CP from GLV_CAT_ strain could be detected, neither in the viral particles sample nor in the total *G. duodenalis* protein lysate. If the CP undergoes a maturation process with the proteolytic cleavage of the first 32 residues, as reported for the original GLV strain [22], we could expect to detect a fraction of the unprocessed CP protein at least in the total *G. duodenalis* protein lysates. Instead, the peptides 33–59 and the 3–29 were the only N-terminal peptides detected for GLV_HP_ and GLV_CAT_, respectively (Supplementary Materials Figure S7), thus suggesting that proline-3 (33 in GLV_CAT_) is the first translated residue. In particular, the peptides 3–29 and 33–59 of GLV_HP_ and GLV_CAT_ capsid proteins, respectively, were the result of a specific tryptic cleavage at the lysine 29 or 59, while the starting amino acid was proline +3, which follows the alanine in position +2 which is not a trypsin-specific cleavage site. Moreover, trypsin activity is usually blocked if a proline is in position P1′ [52], suggesting that these peptides do not derive simply from a not specific trypsin cleavage. To confirm this hypothesis and verify that these peptides come directly from the N-terminus of the proteins, propionylation experiments were performed, and the result obtained from GLV_CAT_ is shown (Supplementary Materials Figure S7). These data, combined with the absence of any spectra derived from peptides containing leucine + 1 and alanine +2, strongly suggested that proline +3 is the translation starting residue for the CP in both GLV_HP_ and GLV_CAT_.

In addition, MS sequence mapping of the GLV_HP_ capsid protein also detected alternative amino acid positions supporting the occurrence of nucleotide heterogeneity in the viral genome (Supplementary Materials Figure S8). Amino acid position 180 (nt position KUA_995–997_) displays either Val or Leu, as shown by the MS/MS spectra of the peptide 174–185 (Supplementary Materials Figure S8); amino acid position 518 (nt ARU_2009–2011_), either Asn or Ser (peptide 515–539 in Supplementary Materials Figure S8); and amino acid position 686 8nt GRC_2513–2515_), either Gly or Asp residue (peptides 678–686 in Supplementary Materials Figure S8). The residue 795 (nt WTA_2840–2842_) remains ambiguous as Leu and Ile, having the same mass, cannot be distinguished. The CP from GLV_P2MER_ was also analyzed by mass spectrometry with a 55% of sequence coverage, although we could not map the N-terminus (Supplementary Materials Figure S9).

### 3.3. An Alternative PRF Mechanism in the Giardiavirus Might Regulate the Expression of the CP/RdRp Fusion Protein

**Figure 3:**
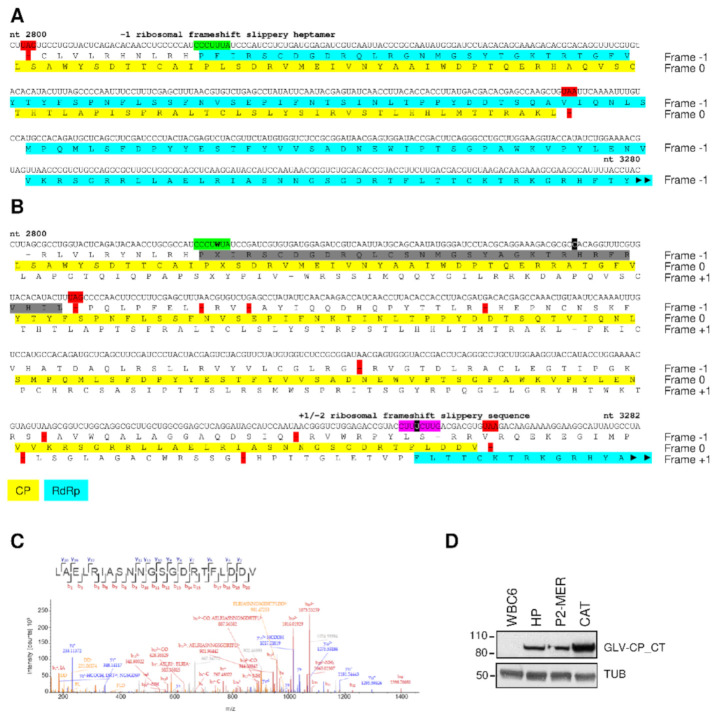
Effect of nucleotide insertions in GLVs on CP sequences and frameshifting mechanism. (**A**,**B**) Overlapping portion of CP and RdRp ORFs and corresponding amino acid sequences in the reference GLV strain L13218 (**A**) and GLVHP (**B**). Nucleotide insertion are in white. Slippery sequences are highlighted: green, originally proposed slippery sequence for the -1 PRF is in green [21]; pink, alternative slippery sequence for the +1/-2 PRF. Stop codons are in red, CP protein sequence is yellow highlighted. (**C**) Mass spectrometry analysis of the GLV CP C-terminus. MS/MS spectrum of the triple charged CP-HP peptide (911–931) derived from digestion with chymotrypsin. The ion at *m*/*z* 755.38 was fragmented and b- and y-series ions are shown in red and blue, respectively. Neutral losses from the precursor ion are shown as green peaks and internal fragments are reported in yellow. The sequence of the peptide matching the fragments detected in the MS/MS spectrum is reported in the panel: the dark lines define the fragments of the b- or y-series ion detected in the MS/MS spectrum. (**D**) Confirmation by immunoblot of the C-terminus of the GLV capsid protein (GLV-CP). Western blot analysis of protein extracts (50 µg) from trophozoites of *G. duodenalis* isolates harboring the viruses (HP, CAT and P2-MER or from control naïve isolate (WBC6). Immunoblotting was performed with mouse anti-GLV-capsid protein C-terminal pAb (GLV-CP_CT). The anti-α-tubulin (TUB) was used as loading control. Molecular size markers (kDa) are reported on the left. The analysis is representative of three independent experiments.

In all the GLV genomes reported in GenBank, the CP and RdRp ORFs show a 220 nt overlap, limited upstream by an UAG stop codon preceding the RdRp ORF and by a downstream UAA stop codon that terminates the CP ORF (Figure 3). This overlapping region contains a CCCUUUA “slippery heptamer” that, in combination with a downstream stem-loop structure with a pseudoknot, has been proposed to promote a -1 PRF and the translation of the CP/RdRp fusion protein [21].

Inspection of our GLV sequences however do not support this mechanism. Despite how the -1 slippery heptamer is conserved in GLV_CAT_ and GLV_P2MER_, it is mutated in GLV_HP_ and GLV_J17/10_A_ (Figure 3 and Supplementary Materials Figure S1). Moreover, in all our GLV sequences a cytosine insertion 65 nt downstream the -1 slippery heptamer results in a premature interruption of the RdRp coding sequence in the -1 frame relative to CP, with the occurrence of multiple stop codons, as exemplified by the GLV_HP_ sequence (Figure 3 and Supplementary Materials Figure S1). The cytosine insertion also alters the CP coding frame resulting in a modified and extended protein C-terminus (Figure 3 and Supplementary Materials Figure S3). This extended C-terminus was confirmed by mass spectrometry in GLV_HP_ and GLV_CAT_, as shown by the MS/MS spectrum of the CP C-terminal peptide (916–931) of GLV_HP_ (Figure 3 and Supplementary Materials Figure S3). Alternative peptides matching the C-terminus of the originally proposed CP sequence (GenBank AAB01578.1) were not found. Furthermore, a mouse pAb raised against the last extended 84 C-terminal residues of the GLV_HP_ CP recognized a single band at the CP molecular size only in trophozoites protein lysate of *G. duodenalis* isolates HP, CAT and also P2MER (Figure 3 and Supplementary Materials Figure S3).

The extension of the CP coding sequence allows for a new and much shorter overlap of 44 nt (nt 307–3250 according to GLV_HP_) with the RdRp ORF now in the +1 frame (bracketed by an upstream UAG stop codon preceding the RdRp ORF and by a downstream UAA stop codon that terminates the CP ORF) (Figure 3). In all our sequences, an uracil insertion within the ORFs overlapping sequence results in a CC_UUU_CUU sequence, that strongly resemble the CC_CUU_UUU “slippery sequence” that allows the translation of the CP/RdRP fusion protein in the all *Trichomonasvirus* TVV1 strains by a −2 PRF [53]. This slippery sequence is compatible with either a +1 or a −2 PRF mechanism. Attempt to address the exact frameshifting mechanism by tandem mass spectrometry was, however, unsuccessful. The low abundance of the CP/RdRp, even using purified viral particles, resulted in a limited sequence coverage compared to the CP, with 42% and 19% of CP/RdRp coverage for GLV_CAT_ and GLV_HP_, respectively (Supplementary Materials Figure S9). No tryptic peptide spanning the CP/RdRp junction was obtained, despite several attempts (Supplementary Materials Figure S9). Nevertheless, partial coverage of the CP/RdRp protein sequence upstream the newly proposed shift site provide evidences to our model (Supplementary Materials Figures S4 and S9).

Moreover, partial coverage of the CP/RdRp C-terminal protein portion support the occurrence of an extended RdRp C-terminus, as a consequence of a guanine deletion in the ORF2 sequence (Supplementary Materials Figures S1, S4 and S9).

### 3.4. Intracellular Localization of GLV in G. duodenalis Isolates

Localization of GLV in the cytoplasm (and at lower intensity in both nuclei) of chronically infected *G. duodenalis* HP trophozoites was originally described by in situ hybridization studies and by transmission electron microscopy [54]. The intracellular localization of GLV in chronically infected *G. duodenalis* HP, CAT and P2MER isolates was hereby studied by immunofluorescence microscopy in trophozoites (harvested at log grow phase) with our anti-CP_NT pAb in combination with a rabbit pAb against 14-3-3, a cytoplasmic protein highly abundant in *G. duodenalis* [40].

**Figure 4:**
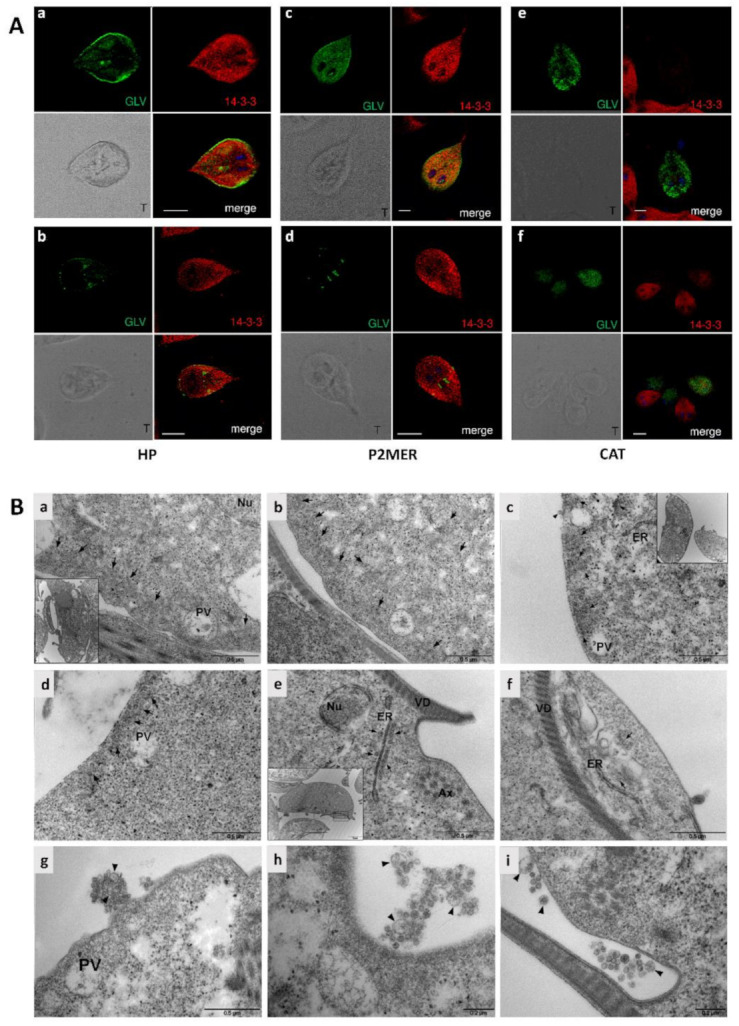
Visualization of GLV in *G. duodenalis* isolates. (**A**) CLSM observations of fixed and permeabilized trophozoites of *G. duodenalis* isolate HP (panels a and b), CAT (panels c and d) and P2-MER (panels d and e). Parasites were stained with mouse α-GLV-CP_NT polyclonal serum (pseudocolor green) and rabbit α-g14-3-3 polyclonal serum (pseudocolor red). Nuclei were DAPI-stained (pseudocolor blue). Displayed micrographs correspond to a single z-stack. T, transmission light acquisition. Scale bars (5 µm) are reported. Images are representative of >50 fields analyzed in two independent experiments. (**B**) Transmission electron microscopy analysis of GLV chronically infected *G. duodenalis* HP and CAT isolates. Panels a and b show CAT trophozoites (inset), at high magnification show numerous scattered GLVs particles in the cytoplasm, also near the peripheral vesicles (PVs) and the plasma membrane (arrows). Panels c and d show HP trophozoites (inset), at high magnification similarly show a very high number of grouped GLVs in the cytoplasm (arrows), prevalently localized under the plasma membrane and between the PVs. Panels e and f show GLV virions (arrows) in *G. duodenalis* CAT isolate adherent to ER cisterna in a trophozoite. g–i: close-up images of GLV virions aggregates outside the trophozoites but in proximity of the plasma membrane in complex with extracellular vesicles (head arrows) and filament-like structures. N, nucleus; ER, endoplasmic reticulum; VD, ventral disk; Ax, axoneme; PV, peripheral vesicles.

GLV immunostaining of the plasma membrane, the ventral disk and areas where flagella exit the cell body was prevalent in *G. duodenalis* HP and P2-MER trophozoites (Figure 4A). A diffuse cytoplasm staining was also observed in 10 ± 5% and 12.5 ± 0.7% of the observed trophozoites, although with different intensity from cell to cell (Figure 4A). Among different experiments, 59 ± 10% and 57 ± 13% *G. duodenalis* HP and P2-MER trophozoites, respectively, did not show any staining, suggesting that virus was at under-detectable level.

A remarkable difference in GLV localization was evident in *G. duodenalis* CAT isolates (Figure 4A). Poor staining of the plasma membrane was observed, and GLV signal was always visible as discrete spots in the cytoplasm of trophozoites, with different intensity from cell to cell. No GLV signal was observed in 55 ± 3.5% of observed cells. In strongly GLV-positive trophozoites, anti-14-3-3 signal was missed (or remarkably faded), suggesting either cells were damaged, and almost devoid of cytosolic protein content, or expression of parasite endogenous proteins was strongly inhibited (Figure 4A). GLV staining was randomly observed in the nuclei of highly GLV positive trophozoites, possibly suggesting entrance of the viral particle following nuclear membrane damage (Figure 4A).

Intracellular localization of GLV was also investigated by TEM in *G. duodenalis* HP and CAT isolates. Trophozoites from both isolates showed a regular ultrastructural aspect by TEM analysis. In CAT, GLV particles were found scattered in the cytoplasm, prevalently near the peripheral vesicles (PVs) and beneath the plasma membrane (Figure 4B, panels a and b), in agreement with CLSM observations. In HP, GLV particles were always gathered together in the cytoplasm, with a prevalent localization under the plasma membrane and between the PVs (Figure 4B, panel c). In ultrathin sections, it was very easily to observe GLVs that seemed to spread directly from the plasma membrane outside the trophozoites (Figure 4B, panel d). In some ultrathin sections of both isolates, tidy trains of GLV particles tightly adherent to the cytoplasmic surface of the ER cisternae and membranes were observed (Figure 4B, panels e and f). Furthermore, aggregates of GLV particle were commonly present outside cells, often tight to microvesicles (MVs), lower than 200 nm in diameter and with filaments compatible with membrane or protein microfilaments (Figure 4B, panels g–i). In highly damaged trophozoites, GLV particles could be observed within the nuclei and tightly adherent to the nuclear membrane (data not shown).

Our attempts to study the intracellular localization of GdRV-1 in *G. duodenalis* J17/10 trophozoites either by immunofluorescence microscopy, using the anti-GdRV-2-ORF1_NT pAb or by TEM, to visualize virion-like particles, were unsuccessful (data not shown). Additionally, following the protocol adopted for GLV purification, no viral-like particles could be purified from the spent culture medium of *G. duodenalis* J17/10 isolates (data not shown).

### 3.5. Infection in G. duodenalis WBC6 Isolate Differs for GLV_HP_ and GLV_CAT_

**Figure 5:**
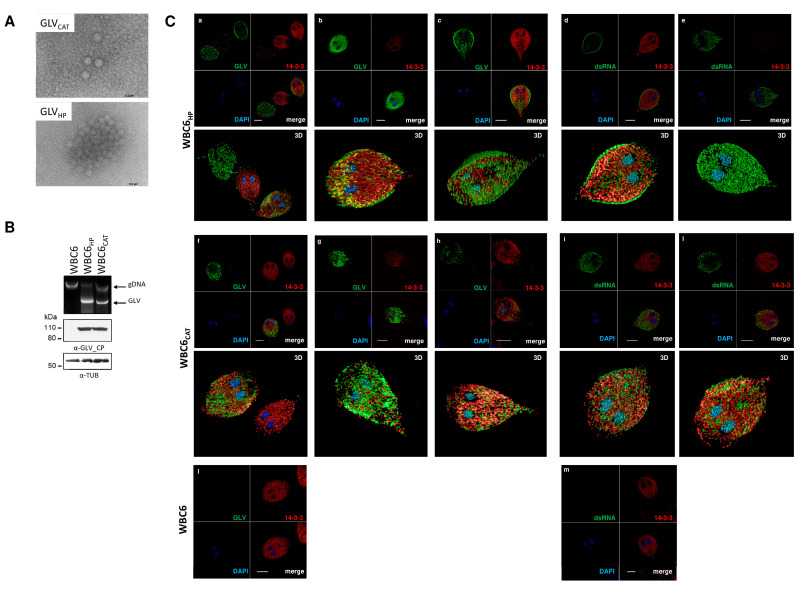
Infection of naïve *G. duodenalis* WBC6 isolate with GLV particles. (**A**) Assessment of GLV particles preparation by negative staining and TEM observation. GLV_CAT_ (viral particle size = 42 ± 4 nm) and GLV_HP_ (viral particle size = 44 ± 3 nm). Scale bars (0.2 µm) are reported. (**B**) Establishment of viral infection. Upper panel, ethidium bromide stained agarose gel electrophoresis (0.8%) of the total nucleic acid (from 2 × 10^6^ trophozoite/lane) isolated from naïve *G. duodenalis* WBC6 and after infection with GLV_HP_ or GLV_CAT_ particles. The band corresponding to *Giardiavirus* dsRNA is indicated (GLV), together with the *G. duodenalis* genomic DNA (gDNA). Lower panel, Western blot analysis of protein extracts (50 µg) from trophozoites of naïve *G. duodenalis* WBC6 and after infection with GLV_HP_ or GLV_CAT_. Protein were separated by 4–12% SDS-PAGE, electroblotted on nitrocellulose membrane and probed with anti-GLV-capsid protein_N-terminal pAb (GLV-CP_NT) and, as loading control, with mouse anti-α-tubulin (α-TUB). Molecular size markers (kDa) are reported on the left. The analysis is representative of three independent experiments. (**C**) Immunofluorescence analysis by CLSM of fixed and permeabilized trophozoites of naïve *G. duodenalis* WBC6 and GLV infected WBC6_HP_ (panels a–e) and WBC6_CAT_ (panels f–m). Parasites were stained with rabbit α-g14-3-3 polyclonal serum (pseudocolor red), either with mouse α-GLV-CP_NT polyclonal serum (pseudocolor green) (panels a–c and f–h) or mouse monoclonal anti-dsRNA (J2) (pseudocolor green) (panels d–e and i–l). Nuclei were stained with DAPI (pseudocolor blue). Displayed micrographs correspond to a single z-stack. Scale bars (5 µm) are reported; 3D, three-dimensional reconstructions of the complete stack series for each acquisition. Different fields of view (>200 cells) were analyzed on the microscope for each labeling condition, and representative results are shown.

To evaluate whether any biological difference might exist between GLV_HP_ and GLV_CAT_ strain, the naïve *G. duodenalis* isolate WBC6 was experimentally infected with viral particles. WBC6 was chosen because it is the most intensively studied *G. duodenalis* Assemblage A isolate [55] and it was originally used for GLV studies [9]. Viral particles, purified from the spent medium of either *G. duodenalis* HP or CAT isolates, were negative contrast stained and analysed by TEM before to carry out the experimental infection. Intact and well-structured virions were mostly observed (Figure 5). However, a remarkable difference between the two viral strains in TEM analysis was noticed, with GLV_HP_ particles tending to clump and to form aggregates, whereas GLV_CAT_ particles appeared more dispersed (Figure 5).

Parasite lines chronically infected with each viral strain were established and designated WBC6_HP_ and WBC6_CAT_, respectively (Figure 5). Immunofluorescence of WBC6_HP_ and WBC6_CAT_ trophozoites with the anti-GLV-CP_NT pAb nicely confirm the peculiar and different intracellular distribution of the two viruses (Figure 5), as already reported in the parental *G. duodenalis* isolates. GLV_HP_ was homogenously distributed in the cytoplasm with an intense staining at the level of plasma membrane (Figure 5). GLV_CAT_ signal was scattered in the cytoplasm and poorly associated with plasma membrane. However, in WBC6_HP_, we could observe a higher number of strongly GLV positive cell, as compared to WBC6_CAT_, in which co-staining with the cytosolic 14-3-3 protein was absent, likely indicating cell ghosts (Figure 5).

Comparable results (Figure 5) were obtained when parasites were immunostained with the anti-dsRNA J2 mAb [56]. Indeed, a more distinct staining of the plasma membrane was evident in the trophozoites infected with any of the GLV strain. Since it is assumed that *Totiviridae* genomic dsRNA is always hidden inside the intact viral particle [57], we might assume that the majority of “intact” infectious-competent viral particles (i.e., viral particle containing the dsRNA) accumulate at plasma membrane.

**Figure 6:**
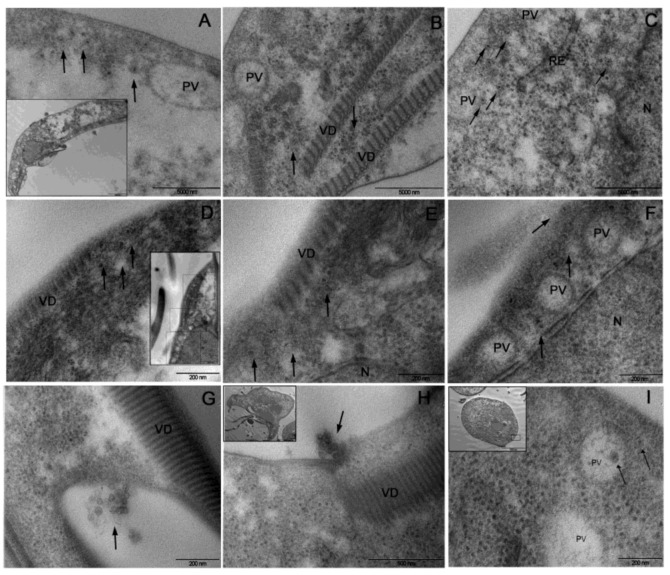
Transmission electron microscopy analysis of *WBC6_CAT_* and *G. duodenalis WBC6_HP_* trophozoites. (**A**–**C**) Electron micrographs of WBC6_CAT_ trophozoites (inset) at high magnification show GLVs particles localized near peripheral vesicles (Pvs) under periplasmatic membrane ad near the ventral dish. (**D**–**F**) Electron micrographs of WBC6_HP_ trophozoites (inset) at high magnification show GLV’s virus particles grouped principally under periplasmatic membrane, near PVs and outside cell near ventral disc area (**G**,**H**). (**I**) shows a detail at higher magnification of a virus particle inside PVs. PV, peripheral vesicles; ER, endoplasmic reticulum; N, nucleus.

Transmission electron microscopy analyses were in agreement with CLSM observations. Although, no relevant morphological changes associated with the infection were observed in *G. duodenalis* WBC6_HP_ and WBC6_CAT_ trophozoites (Figure 6). In WBC6_CAT_ trophozoites, viral particles were scattered in the cytoplasm and poorly aggregated. Although distributed over the whole cell body, pools of viral particles were observed near PVs and the ventral disk (Figure 6A–C). In WBC6_HP_ trophozoites, GLV_HP_ viral particles were strongly clustered and localized near the PVs and the ventral disc (Figure 6D–F).

Taken together, microscopic observations indicate that intracellular distribution of viral particles is exclusively ascribable to unique properties of each virus. Release of GLV_HP_ viruses from the parasite plasma membrane was observed in close proximity with the ventral disk (Figure 6G,H) and in association with MVs (data not shown), further suggesting that virus could egress the infected host via budding at plasma membrane or employing MVs budding. Additionally, in a very limited and not statistically significant microscopic fields, single GLV_HP_ particles were found within the lumen of the PVs (Figure 6I).

**Figure 7:**
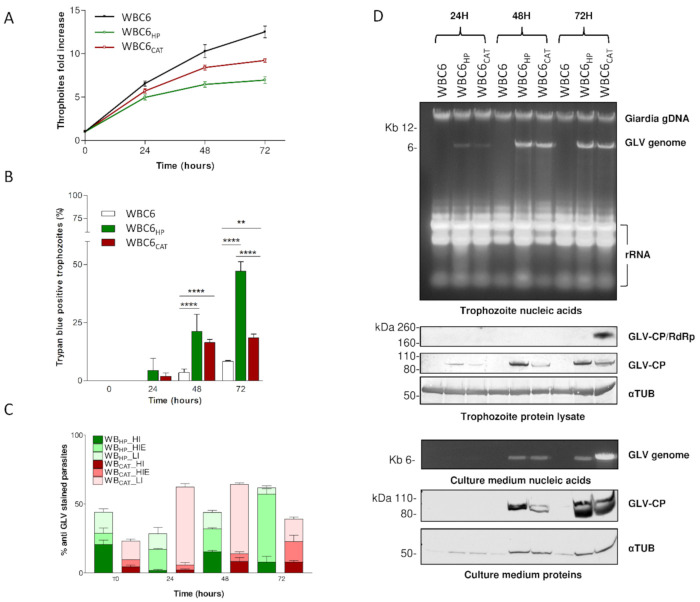
Time course of GLV infection in constitutively WBC6_HP_ and WBC6_CAT_ trophozoites. (**A**) Growth curve of naïve *G. duodenalis* WBC6 isolate (■), WBC6_HP_ strain (○) and WBC6_CAT_ strain (□). WBC6_HP_ and WBC6_CAT_ trophozoites constitutively infected with GLV (after 12 passages from infection) were used. Experiment was conducted in triplicate. Parasite were counted in hemocytometer every 24 h, as reported in Materials and Methods section. (**B**) Percentage of trypan blue positive trophozoites counted throughout the parasites growth curve. ** *p* < 0.01; **** *p* < 0.0001. (**C**) Percentage of WBC6_HP_ and WBC6_CAT_ trophozoites showing or not staining for the rabbit anti-g14-3-3 polyclonal antibody and/or the mouse anti GLV-CP_NT polyclonal serum by immunofluorescence analysis throughout parasites growth curve. Parasites were classified based on staining as follows (see main text for details): HIE, highly infected but empty, with strong GLV signal but faint or absent staining with for 14-3-3; HI, highly infected, with strong signal for both GLV and 14-3-3; LI, low infected, with faint or spotted GLV signals and strong staining for 14-3-3. (**D**) Total nucleic acids analysis by agarose gel electrophoresis (0.8%) and protein expression analysis by SDS-PAGE (4–12%) and immunoblot throughout parasites growth curve. Equal amount of trophozoites (2 × 10^6^) and ultracentrifuged culture medium (3 mL) were processed to extract either the total nucleic acids or the total proteins, as described in Materials and Methods. For the nucleic acids analysis, bands corresponding to *G. duodenalis* genomic DNA (gDNA), GLV genome and ribosomal RNA (rRNA) are indicated. Nucleic acids’ molecular sizes (in kilobases, Kb) are reported on the left. Immunoblot analysis was performed with the mouse anti-GLV-CP_NT and mouse anti-α-Tubulin. Bands corresponding to the CP/RdRP, the CP and α-Tubulin are indicated. Protein molecular sizes (in Kilo Dalton, kDa) are reported on the left.

We also examined the effect of viral infection on parasite growth. In comparison to the uninfected parental isolate, chronic infections with both GLV_HP_ and GLV_CAT_ resulted in the reduction of parasites growth (Figure 7). This effect was, however, more prominent for WBC6_HP_ than for WBC6_CAT_, with the parasite replication rate at 72 h reduced by 45 ± 8% and 27 ± 4%, respectively (Figure 7). Particularly for WBC6_HP_, this impaired growth was associated to a remarkable increase in the percentage of trypan blue permeable trophozoites (47 ± 5% in WBC6_HP_, 18.5 ± 3% in WBC6_CAT_, 8.3 ± 0.8% in WBC6, at 72 h), indicating either death or high damage (Figure 7). Simultaneously, the GLV infection level was evaluated by immunofluorescence. Trophozoites were classified as (i) “highly infected but empty” (HIE), consisting of cell “ghosts” strongly stained by the anti GLV-CP_CT pAb, indicating virus iper-infection, but showing a faint or absent staining with the anti-14-3-3 pAb, indicating parasite voided of giardial protein content; or (ii) “highly infected” (HI), consisting of cells strongly stained both by the anti GLV-CP_CT and anti-14-3-3 pAbs; or (iii) “low infected” (LI), consisting of cells with faint or spotted GLV signals and strong staining by the anti-14-3-3 pAbs. Whereas, the percentage of HIE trophozoites constantly increase for WBC6_HP_, reaching almost the 50% of cells at 72 h, the percentage of HI and LI trophozoites peaked at 48 h and then decreased at 72 h, suggesting a constant accumulation of GLV particles within the cells (Figure 7). These observations were in agreement with Western blot analysis of trophozoite’s protein content and gel electrophoresis of nucleic acids, showing an increasing accumulation of capsid protein and GLV genome over time, associated with the release of viral particles in the medium (Figure 7). On the contrary, in WBC6_CAT_ population, the majority of trophozoites were classified as LI (representing >50% at 24 and 48 h). A limited increase of HIE trophozoites (≈15%) was observed at 72 h (Figure 7). Quantification of intracellular GLV RNA and CP proteins confirmed the immunofluorescence observations; however, in the extracellular medium, the amount of GLV_CAT_ genome was much higher in comparison to the amount of detectable CP, suggesting that a higher proportion of “intact” viral particles might be release by WBC6_CAT_ respect to WBC6_HP_ (Figure 7). A higher efficiency of GLV_CAT_ to assemble “intact” viral particle is also suggested by the presence of a visible band at a size compatible with the CP/RdRp protein only in protein lysate of WBC6_CAT_ at 72 h (Figure 7). This overexpression of CP/RdRp was also observed in *G. duodenalis* CAT isolate (Figure 1). Occurrence of a higher amount of alpha-tubulin in the medium of WBC6_CAT_ and WBC6_HP_, in respect to the WBC6 isolate, pointed out either an increased cell lysis of the infected parasites or the release in the medium of microtubules associated with viral particles.

To provide an explanation for the different behavior of GLV_HP_ and GLV_CAT_ viral particles, automated protein-structure homology modeling of both CP proteins was performed. Despite a low primary-sequence homology, capsid organization among *Totiviridae*, including GLV, is highly similar, with GLV CP subunits showing a remarkable resemblance, on assembly and secondary structures, to those of *Saccharomyces cerevisiae* L-A virus, HvV190S, and *T. vaginalis* virus TVV1 [23]. Only the sequence of CP from residues 364 to residue 900 could be modeled returning models in monomeric form (Supplementary Materials Figures S4 and S10). Four protein segments (residues 465–471, 543–553, 650–654 and 783–792), included in the models, showed sequence divergence between GLV_HP_ and GLV_CAT_ (Supplementary Materials Figures S4 and S10). Based on the superimposition with the ScV-L-A CP monomer A, the CP surface exposed inside and outside the capsid could be defined (Supplementary Materials Figure S10). The fragment 543–553 might form a positively charged loop exposed on the GLV_CAT_ virion outer surface, eventually providing electrostatic repulsion between the virions that could support the tendency of GLV_CAT_ to not aggregate (Supplementary Materials Figure S10). The same protein fragment is instead buried within the protein in GLV_HP_, resulting in a flat, less-charged surface. However, these observations should be taken with caution due to the low reliability of the models (QMEAN~-10), and only experimentally defined high-resolution structures for both GLV_CAT_ and GLV_HP_ virions might confirm or reject this conclusion.

## 4. Discussion

High-throughput sequencing (HTS) has now become a standard method for identifying novel viruses (RNA or DNA) and generating full-genome assemblies of viruses in many contexts [58]. In the present work, by the use of HTS Illumina technology, we provided a vast improvement in the knowledge of GLV genomes. With particular emphasis on GLV_HP_, HTS shows few but relevant discrepancies (two or three nucleotide insertions and one deletion) from the first deposited GLV genomic sequence, L13218.1, as well as from the AF525216.1 and DQ238861.1. Illumina-based approaches produce very high depth and quality coverage when compared with the early Sanger sequencing technology that, combined with PCR and cloning strategy originally adopted to sequence the first GLV genome, would explain potential inaccuracy in the previously reported genome(s) [10,45]. As a consequence, sequencing errors in the L13218.1 might have affected data analysis and interpretation, in particular on current understanding of regulatory mechanism governing translation of GLV proteins.

Our genome-sequencing data, with the additional evidences provided by mass-spectrometry analysis, suggest that IRES-mediated translation of GLV capsid protein, independently from the GLV strain, starts from the unusual AUG-unrelated codon CCU encoding for a proline residue instead of the early-proposed AUG codon [10]. Indeed, previous proteomic analysis of GLV virion could also not identify the putative N-terminal 32 residues of the capsid protein [23]. Additionally, the possibility that CP could start at Pro_33_ (according to deduced ORF1 of L13218.1) was proposed in an earlier report [22] since the CP could not be recognized by an antibody against the first N-terminal residues (residues 6–27) and CP micro-sequencing yielded as N-terminal sequence the peptide P_33_ENITFDT_40_. However, this hypothesis was rejected in favor of a post-translational maturation process of the capsid protein by proteolytic cleavage of the first 32 N-terminal residues by an uncharacterized *G. duodenalis* protease [22]. Although further experiments are necessary to fully confirm our model, methionine-independent translation initiation, promoted by an IRES in an intergenic region (IGR), has been extensively described for the capsid protein of *Dicistroviridae*, a family of ssRNA(+) picorna-like viruses of invertebrates [59]. In these viruses, IGR-IRES forms an RNA pseudoknot-like structure (by means of a base-pairing involving a triplet upstream to the translation initiation) that occupies the P-site of the ribosome, allowing translation start from the ribosome A-site, using a codon encoding either Ala or Glu [59]. Indeed, this *Dicistroviridae* IGR-IRES structure resembles our predicted IRES folding, in particular for the GLV_HP_. In addition, and similar to *Dicistroviridae* [60], it is worth mentioning that the translation initiation inhibitor edeine, which prevents tRNA binding to the ribosomal P-site, has been previously shown to not significantly affect the function of GLV IRES in *G. duodenalis* [19], further suggesting that GLV capsid protein translation might initiate from the ribosome A-site. However, we cannot yet exclude that translation start from the upstream near-cognate AUG initiation codon UUG_464–466_ (according to GLV_HP_ sequence), and methionine is initially incorporated by ribosomal interaction with tRNA_i_^Met^ and then removed by methionine aminopeptidase (MetAP) activity by leaving the observed N-terminal amino acid of the capsid protein. Indeed, a type-2 MetAP is encoded by *G. duodenalis* but its function has not been elucidated so far. However, this possibility is unlikely to occur as removal of the first Met from GLV capsid protein should leave Ala, and not Pro, as first amino acid, but no propionylated peptide starting with APEN were found and, additionally, MetAPs do not efficiently remove Met when the residue at +2 position is a proline [61].

Our sequencing and proteomic data also challenge the current -1 PRF model proposed by Wang et al. [10,21] as the mechanism governing the low-level expression of the RdRp. This model involves a hepta-nucleotide RNA slippery sequence, C_2836-_CCU-UUA_2842_ (codon breaks in frame 0 are indicated by dashes) in association with a downstream pseudoknot RNA structure (nt 2848 to 2876), both located in a 220 nt overlapping region between the ORF1 and ORF2, according to L13218.1 [10,21]. The RNA pseudoknot promotes ribosomal stall that stimulate the ribosome to slide back one base at the end of the slippery sequence and then read CCC or UUU as the next codon. Despite this -1 slippery sequence is conserved in the genome of GLV_CAT_ and GLV_P2MER_, it is mutated in GLV_HP_ [C-CCU-(U/A)UA] and GLV_J17/10A_ [C-CCU-AUA]. In addition, in all GLV strains, the two downstream nucleotide insertions, C_2909_ and U_3239_ (according to GLV_HP_ sequence), alter ORF1 reading frame extending its 3′ end, that lead to a capsid protein with an extended C-terminus, and prevent translation of the RdRp by any -1 frameshifting. The original 220 nt overlapping region within ORF1 and ORF2 [10,21] is then reduced to only 44 nt (3207–3250 according to GLV_HP_ sequence) and must contain a slippery motif that might promote the required access to the +1 frame for RdRp translation by either +1 or −2 PRF [62]. Although reported less frequently and far less characterized, the +1/−2 PRF occurs in various dsRNA and ssRNA(+) virus families, with −2 PRF differing from +1 PRF, as, in the first case, an additional amino acid is encoded at the shift site [62]. In the current model, ribosomes are forced to pause at rare codons waiting longer for the correct cognate, and correspondingly rare, tRNA. Such a delay can result in tRNA dissociation from the mRNA within the ribosome, ribosome sliding ahead or behind along the mRNA and rebinding of the tRNA(s) elsewhere on the mRNA. Translation then continues with the production of a polypeptide other than that specified by original mRNA decoding [63]. For instance, PA-X gene expression of influenza A virus is dependent on +1 PRF promoted by the UCC_UUU_CGU sequence, in which slippage occurs on UUU_C with UUU in the ribosome P-site and A-site either empty or occupied by slowly decoded and rare CGU codon for alanine [64]. Among *Totiviridae* the expression of the RdRp by a +1 PRF, centered on CCC_GAA sequence, was suggested to occur for a strain of *Leishmaniavirus* LRV1-4 ([65]. In *Trichomonasvirus* TVV1, expression of the RdRp occurs via a −2 PRF promoted by the slippery sequence CC_CUU_UUU, located in a very short overlapping region (14 nt) between CP and RdRp ORFs [53]. The mechanism, confirmed by peptide mapping of the CP/RdRP junction, involves a ribosome sliding back two bases at the end of the slippery sequence, following pausing and the addition of the Phe encoded by the UUU codon, thus reading UUN as the next codon [53]. Despite we could not identifying the tryptic peptide that spans the CP/RdRp junction, and then define the slippage mechanism, the proposed ACC-UUU-CUU-GAC sequence present in the ORF1/ORF2 overlapping region in our GLV stains possesses the necessary characteristics to promote either +1 or −2 PRF mechanism. Slippage may occur following ribosome stall while awaiting delivery of tRNA complementary to either the UUU codon for Phe or the CUU codon for Leu, both seldom-used codons in the genomes of *G. duodenalis* [66]. For instance, if UUU is the shifty codon, slippage of the ribosome by one base in the 3′ direction (+1 slippage) from to UUU to UUC, a highly used codon for Phe, can resolve the pause [64]. Less likely to occur, +1 slippage by jumping of one base after UUU will accommodate in the ribosome the mid-used UUG for Leu [66]. A similar consideration might be argued if slippage follows the −2 mechanism described in TVV1. Differential expression level and availability of specific tRNA (e.g., UUU-tRNA^Phe^) in *G. duodenalis* hosts with slightly different genetic phenotypes (as it can occur between strains and Assemblages [66] could eventually affect the RdRp expression and consequently the virus replication efficiency. Beyond the putative slippery sequence and tRNA abundance, other features might anyway regulate the expression of the RdRp in GLV as we observed a higher level of the polymerase fusion protein in trophozoites infected with GLV_CAT_ compared to GLV_HP_. Since this effect was observed independently from the parasite background, nucleotide differences within the GLV strains and the presence of cis-elements within the GLV_CAT_ mRNA, such as secondary structures downstream the slippery site, might promote or hamper the frequency of PRF.

We also reported on the occurrence of single nucleotide polymorphisms (SNPs) in GLV_HP_ and GLV_J17/10A_ genomes. Observed SNPs could be either the result of low replication accuracy and high error rate common to RNA-dependent RNA polymerases [67] and/or the effect of the co-infection with more than one GLV genotype in each parasite isolate. The latest hypothesis is somehow supported by a previous report [68] where two types of GLV particles, namely p100 and p95, differing in CP proteins size, peptide mapping profiles and secretion in the medium, were described in the *G. duodenalis* WB strain experimentally infected with viral particles from the *G. duodenalis* HP isolate. However, these two putative distinct viruses were not individually defined at genomic level [68]. Co-infection of multiple TRV strains in the same *T. vaginalis* cell is also well documented [69]. However, since neither HP nor J17/10 *G. duodenalis* isolates are clonal lineage, we cannot exclude that SNPs are due to the occurrence in the parasite culture of two or more sub-populations of trophozoites infected with only one of the viral genotype.

Viral genomes and infection phenotypes clearly support the existence of two GLV subtypes, being represented by GLV_HP_ and GLV_CAT_, respectively. Indeed, early report on infection with uncharacterized RNA viruses from different *G. duodenalis* isolates, suggested that differences in growth adaptation of naïve *G. duodenalis* isolates could be associated to variability in each virus infectivity [28]. Sequencing of more GLV strains from different *G. duodenalis* isolates/Assemblages (in particular Assemblage B) will indeed help to strength our conclusions.

Concerning the different viral infection phenotypes, the milder cytopathic effect we report in the WBC6_CAT_ strain could be associated to the limited intracellular accumulation of GLV_CAT_ viral particles, due to an efficient release of the viral particles outside the cell. This is in agreement with previous observations that accumulation of high amount of GLV ceases *G. duodenalis* trophozoites division and cellular function, leading to the arrest of parasite growth [25,29]. The more cytopathic effect associated to GLV_HP_ infection could be explained by an impaired release of viral particles that remain trapped inside the parasite, accumulating faster than they are released. Alternative strategies for virions egress has been described for non-enveloped naked viruses hijacking different types of extracellular vesicles, such as microvesicles (MVs), secretory autophagosomes and exosomes for non-lytic release [70,71,72]. The tight association of GLV particles with MVs outside of the cell might suggest GLV can exploit the MVs budding mechanism or stimulate MV budding. The putative deficiency in GLV_HP_ egress, leading to virions accumulation at cell periphery beneath the plasma membrane, could be associated to the tendency of viral particles to aggregate, as observed by electron microscopy and due to low repulsion forces at the virion surface, preventing viral exploitation of MVs secretion.

For instance, LVR1 can exploit exosome secretion pathway to be released from *Leishmania* and survive longer in the extracellular environment thus behaving as an envelope-like virus, facilitating LRV1 transmission and increasing infectivity in the host [73]. Despite we observed GLV particles in close proximity to PVs and PVs are proposed to be involved in exosome biogenesis in *G. duodenalis* [74], we could not provide, at this stage, any strong evidence for GLV release via exosomes. More focused research is needed to define the egress of GLV as a key process for virus stability, infectivity and transmission in the gut harsh environment of the human or animal *G. duodenalis* hosts.

We report here for the first time, a new putative RNA virus, GdRV-2 in at least one isolate of *G. duodenalis* Assemblage E, co-infected with GLV. Based on the sequence homology of the RdRp domain in the expressed GdRV-2 large protein, the virus could be closely related to *Totiviridae.* Nevertheless, the absence of an independent ORF encoding for a putative capsid protein and the lack of evidence of capsid-like structures in the infected *G. duodenalis* trophozoites, might suggest that GdRV-2 belongs to a new unclassified family of capsid-less virus, such as the ssRNA(+) *Narnaviruses* and dsRNA *Hypoviruses* in fungi and dsRNA *Endornaviruses* in fungi and plants [75]. RdRp is the only one gene shared by ssRNA(+) and dsRNA viruses and is the only represented in all capsid-less RNA genomes, although capsid-less viruses (and retrotransposable elements) have been shown to have complex and different evolutionary scenarios [75]. We exclude that GdRV-2 is a new retrotransposon element. Despite retrotransposon sequence [76] and virus of the CRESS family are present in the *G. duodenalis* genome [77], no trace of the GdRV-2 sequence was detected in the genome of *G. duodenalis* isolate J17/10, neither by PCR assay nor by whole-genome sequencing (data not shown). Instead, co-infection of *Totivirus* with satellite replication defective virus has been well documented in *Saccharomyces* yeasts [78]. Some of these satellite viruses did not encode any protein but only RNA sequences that enable their encapsidation and replication, while others encode antifungal “killer” toxin proteins [78]. Recently, co-infection of *Totiviridae* with capsid-less *Narnaviridae* has also been reported for the trypanosomatid parasite *Blechomonas* spp. [79]. Persistence and transmission of capsid-less RNA virus is however challenged by host RNA interference (RNAi) machinery, that recognizes and degrades dsRNA (either viral genomic or transiently generated during ssRNA viral replication) [80]. The problem is overcome by ScNV-23S and ScNV-20S *Narnaviruses*, persistently infecting several *S. cerevisiae* strains [81], by packing viral ssRNA genome in a ribonucleoprotein complex with the RdRp (the only protein encoded by the virus), thus preventing viral RNA degradation by host cytoplasmic exonucleases [81]. Noteworthy, in *G. duodenalis* RNAi machinery is present but does not work efficiently [82,83]. It would then be relevant to verify if the expressed putative RdRp (ORF1) of GdRV-2 can ensure viral replication and possibly form a protective ribonucleoprotein complex with the viral genome, thus allowing vertical transmission of GdRV-1 during trophozoite binary fission. Alternatively, due to co-infection with GLV and GdRV-2 genome having a size comparable to GLV, RdRp might be packed accidentally within GLV capsid instead of the GLV genome, allowing few GdRV-2 genome copy to be protected and inherited in the *G. duodenalis* progeny. More efforts will be needed in the future to yield additional GdRV-2-like sequences, verify its distribution among different *G. duodenalis* Assemblages and allow the characterization of this virus from a functional and evolutionary perspective.

## 5. Conclusions

Our study provides new evidence on GLV genome organization and biology, highlights the importance of performing in deep transcriptomic studies to reveal the potential diversity of viral infections in the protozoan parasite *G. duodenalis* and strengthens the possibility that GLV, or other endosymbiont virus infections, can be associated with alteration of particular phenotypic *G. duodenalis* traits, including virulence.

## Figures and Tables

**Table 1:** List of *G. duodenalis* isolates used in the present work.

Isolate	Origin	Assemblage	GLV Positive	Reference
WBC6 (ATCC-50803)	Human	AI	No	
HP	Human	AI	Yes	[35]
CAT	Cat	AI	Yes	[36]
P2-MER	Pig	AI	Yes	[31]
P15	Pig	E	No	[37]
J17/10	Sheep	E	Yes	[31]

**Table 3:** List of heterozygous nucleotide position in GLV genomes.

GLV Strain	Heterogeneous nt Positions	Predicted aa Substitution	
		ORF1	ORF2
CAT	--	--	--
HP	11	4	--
P2MER	--	--	--
J17/10_A	12	1	2

Abbreviations: nt, nucleotide; aa, amino acid.

## Data Availability

The data presented in this study are available as Supplementary Materials that can be found at https://www.mdpi.com/article/10.3390/biomedicines9060654/s1. Raw sequencing data (fastq file format) are available at https://www.ncbi.nlm.nih.gov/sra, under the BioProject accession number PRJNA720885 (27 April 2021). Viral genomes can be found at https://www.ncbi.nlm.nih.gov/genbank/, under the accession numbers from MW659703 to MW659707 (25 February 2021). Proteomics data were deposited at the ProteomeXchange Consortium via the PRIDE (http://www.proteomexchange.org/), under the accession number PXD025785” (4 May 2021).

## Supplementary Materials

The following are available online at https://www.mdpi.com/article/10.3390/biomedicines9060654/s1. Excel File S1: Blast-P report for JA1710_B RdRp; Supplemental Figures Captions. Figure S1: Multiple sequence alignment of GLV genomes. Figure S2: Phylogenetic analysis GLV strains. Figure S3: Multiple alignment of the CP (ORF1) amino acid sequences. Figure S4: Multiple alignment of the RdRp (ORF2) amino acid sequences. Figure S5: Circular tree graphical view of the BLAST pairwise alignment. Figure S6: Pseudoknots prediction in the GLV 5′-UTR. Figure S7: Mass spectrometry analysis of CP proteins of GLV_HP_ and GLV_CAT_ strain. Figure S8: Mass spectrometry analysis of heterologous position of the GLVHP CP. Figure S9: Mass spectrometry analysis of CP of GLV_P2MER_ and CP/RdRp proteins of GLV_HP_ and GLV_CAT_ strains. Figure S10: Virtual modeling of GLVHP and GLVCAT CP monomer. Table S1: RNAseq sequencing results. Table S2: Primer used for amplification and Sanger sequencing. Table S3: Primers list for antigens amplification. Table S4: Amount of reads mapping on reference viral genome.
