# Supplementary material for: Re-Discovery of Giardiavirus: Genomic and Functional Analysis of Viruses from Giardia duodenalis Isolates
Source: Biomedicines. 2021 Jun 8;9(6):654. doi: 10.3390/biomedicines9060654 (PMC8230311; doi:10.3390/biomedicines9060654)
Supplement: Supplementary file 1 [file biomedicines-09-00654-s001.zip › Supplemental Files/Figure S1.pdf]

```
L13218.1     1 GGAAGGAGUGCCAGGCCAUUACCUUCCGCCCCUGCCUAACAAAACUGGCUCGCCAGAGGCGGGGGGGCGUGGUAGACGGUAUGUCUAGGUCCAGAGAAAGCGGCCGCUGUUAGGCUGCUA
AF525216.1   1 .......................C..........................UA...................................................U........A..C...G
DQ238861.1   1 .......................C..........................UA...................................................U........A..C...G
CAT          1 .......A...........................................................................................A.....A..............
HP           1 .......A..........................................UA...................................................................G
P2MER        1 .......A.............................G............UA....................C...................................A..........G
J1710_A      1 .......A..........................................UA...................................................................G

L13218.1   121 GACUUCGCCACGCUUACACCAUUGAGAACACAUAGGUGGUGUAAUAUUAUGUGUUUGGCUGCGUGUGUCACGAGCACGAUCUAUGGGGGAAUCUAACCCAGGUGGUGUUGUGUAUGAUGA
AF525216.1 121 ......................C...........A............................................C.C....A...............................A.
DQ238861.1 121 ......................C...........A............................................C.C....A...............................A.
CAT        121 ......................C.................................................................................................
HP         121 ......................C...........A..............................................C....................................A.
P2MER      121 ......................C...........A..............................................C....................................A.
J1710_A    121 ......................C...........A..............................................C....................................A.

L13218.1   241 GGUACAAGCCCGUCAACACCACAGGUGGAGAGGGACAGGAGUCUCAUUUCCCCUCCAAAAAACAGCAGUGGUCCGCCCCUCAUGCCCUAUGAAACAUUAGGGGGCCGACACUAGUGGGCA
AF525216.1 241 ...........................................C...........U.G.....................................G........................
DQ238861.1 241 ...........................................C...........U.G.....................................G........................
CAT        241 ...........................................C.........CUUU......................................G........................
HP         241 ...........................................C...........U.G.....................................G........................
P2MER      241 ...........................................C...........U.G.....................................G........................
JA1710_A   241 ...........................................C...........U.G.....................................G........................

                                                                                                 First cytosine insertion and premature CP ORF stop
L13218.1   361 AGCCCGAUGGUGUGGGGACUGGAUAUGGGAACCCCAGUCCGUUAAACCCGUACGGCUUCGCCAGCCUACACCGGGGGGGCUUAAUCCCCUUAUU---UUGGCACCUGAAAAUAUUACU
AF525216.1 361 ................................................................................................---.....................
DQ238861.1 361 .................................A..............................................................UUU.....................
CAT        361 ................................................................................................---..............C......
HP         361 .................................A..............................................................C--........UGA..........
P2MER      361 .................................A..............................................................C--........UGA..........
J1710_A    361 .................................A..............................................................C--........UGA......Y...

L13218.1   478 UUUGACACUUUAAACACACACAAUGAUCACGAGGAGACCCAUGGAGAGUCUCCAGAGGUUCCUAAGGCCUCGAUCGCGCCUGCCGGGAGGCAGAAUGUCCCGGUUCUCCAACAAAAUCAA
AF525216.1 478 ...........G........G.....C............U.C..........................................................................A...
DQ238861.1 481 ...........G........G.....C............U.C..........................................................................A...
CAT        478 C.............U.....G..C..CU.............C..............................................................................
HP         479 ...........G........G.....C............U.C..............................................................................
P2MER      479 ...........G........G.....C............U.C..............................................................................
J1710_A    479 ...C.......G........G.....C............U.C..............................................................................

L13218.1   598 GAAAACGAAGAUAAUCACGCGCUGGGCGGUAGCGAGGACGCCAAAGAUGAGCGCGAAAUACGAUUCUCAGCCAUAAAAACUCUCUACAAUUAUUACUCAGAGGGUCCUUCGACCCCAAUU
AF525216.1 598 .............................C.................C.............AG..U.....U.............................A..........U.......
DQ238861.1 601 .............................C.................C.............AG..U.....U..U..........................A..........U.......
CAT        598 ......A........................................C..................................................G.....................
HP         599 .....Y.......................C.................C.............AG.CU.....U.............................A..................
P2MER      599 ............G................C.................C.............AG..U.....U........C....................A..................
J1710_A    599 .....U....................Y..C.................C.............AG.CU.....U.............................A..................
```

```
L13218.1    718 AUGCCGCAUUUAGUUAACAGGUUACGUGGAUUAGAUGCCCUGGCCAAGAUAGACGCGACGCUGACCAAGGUGGAUAUGAACGCGGCCUACAUAUUCGCCCUGAGACCGACUUUUCCGUAU
AF525216.1  718 .....A........C...........A...........U..A......G...............G.......................................................
DQ238861.1  721 .....A........C...........A.......U...U..A......G...............G...............................C.......................
CAT         718 ..............C...........A...........U..A..............................................................................
HP          719 .....A........C...........A...........U..A......G...............G..........................................R............
P2MER       719 .....A........C...........A...........U..A..U...G...............G.......................................................
J1710_A     719 .....A........C...........A...........U..A......G...............G..........................................A............

L13218.1    838 UCGUAUGGGUAUAAACAGCGCUUCAGUAACAGGAGAUUAACCACUAGUGCGCUUUGCUACGCAAGGACGGGUCUAUCCUCAUUUCUGACCGUCGACAAAACGUACACAUCUAACUCUCCA
AF525216.1  838 .................A..U.................G.............................A..C.................A.................G..C.........
DQ238861.1  841 .................A..U....C............G.G...........................A..C.................A.................G..C.........
CAT         838 ......................................G............G...................C.....................A................C.........
HP          839 .................A..U.................G.............................A..C.................A.................G..C.........
P2MER       839 .................A..U.................G.............................A..C.................A.................G..C.........
J1710_A     839 .................A..U.................G....................Y........A..C.................A.................G..C.........

L13218.1    958 UUAAAAGGGGGCUCUAGAGGGUGGCCCAUUUUCAACGUGGAUGUUUCAUCACACGUGGCGGAGCCUCACAUGAGGACAUUGUCACCUAUCGGUCUUGAAGUAUUCAAUCUGGCUACGUCU
AF525216.1  958 ................................U...A.A.G......GC...................................................................U...
DQ238861.1  961 ................................U...A.A.G......GC...................................................................U...
CAT         958 ......................................A.G......GC................C................................G.....................
HP          959 ....................................K.A.G......GC...................................................................U...
P2MER       959 ....G.................................A.G......GC...................................................................U...
J1710_A     959 ..............Y.....................T.A.G......GC..........K........................................................U...

L13218.1   1078 CAAUUUUCGAAAACUCUAUUAACUGCAUCAUCGAAAGUCUUCACGCAAUCGCUCUACACUGCUGACAUACUAUCGAUCUUUGGUGAGGUGUUCCUGCCACACGUGAUGCAACCGGUCUCA
AF525216.1 1078 ..G............................................G..A.....................................................................
DQ238861.1 1081 ..G............................................G..A.....................................................................
CAT        1078 ..G............................................G..A.....................................................................
HP         1079 ..G...............................................A.....................................................................
P2MER      1079 ..G...............................................A......U.......T......................................................
J1710_A    1079 ..G...............................................A.....................................................................

L13218.1   1198 AACUACACUCCAAUACUUGUGAGGGCACUAUUGGCCUUGAUCCACAUACUGGGGCCAGGAUCUGGUAAUUGCUCACUUUCAUCGUCUAUCUUCGAGAGCUCAAUUCCACAAUUCCUUACG
AF525216.1 1198 ..........................G.................................................................U.....U...............U.A...
DQ238861.1 1201 ......C...................G.................................................................U.....U...............U.A...
CAT        1198 ..........................G.......................................................................U...............U.....
HP         1199 ..........................GY..........................U.....................................U.....U...............U.A...
P2MER      1199 ..........................G...........................U......................C..............U.....U...............U.A...
J1710_A    1199 ..........................GU..........................U.....................................U.....U...............U.A...

L13218.1   1318 GUAAGUCAUAGUACGAACAUGUCGAAUAGAACGAGGUACUGCUUACAUACGAGGUCUGCUUACAAGGAUAUGUUCAGGAACGGUAUCCCACCUCAGUCGACGUUGCCACCGACGCUCGCG
AF525216.1 1318 A....C........A...........C.......................AU.........................A..........................U...............
DQ238861.1 1321 A....C........A...........C.......................AU.........................A.U...............C........U...............
CAT        1318 ..............A...........C....................C..AU....C....................A..U.......................................
HP         1319 A....C........A...........C.......................AU.........................A..........................U...............
P2MER      1319 A....C........A...........C.......................AU.............A...........A..........................U...............
J1710_A    1319 A....C........A...........C.......................AU.........................A..........................U...............
```

```
L13218.1   1438 CCGGAGGGGUCCUCUGCUCGGGUUUUAAUCCCAGAGGCCCUAGUGACCAGCCCUAUGUUUCCAUGGUUACUUAUCUUAGUCUCGAGCGGCCCACAGUUCUUCUUGUAUUCAAAGGACGCU
AF525216.1 1438 ..A..................A..C.........C.....................................G.A.............................................
DQ238861.1 1441 ..A..................A..C.........C.....................................G.A.............................................
CAT        1438 ..A.....................C.........C...G........U.....C..................GC..............................................
HP         1439 ..A..................A..C.........CR..G........U.....C..................G.A.............................................
P2MER      1439 ..A..................A..C.........C...G........U.....C..................G.A.............................................
J1710_A    1439 ..A...............M..A..C.........CA..G........U.....C..................U.A.............................................

L13218.1   1558 AGUAUUAAUACCGUAGACAUCGGUUCGAGGGGGAGGAUUACAUCACCAAUCCCUGACGUCGCCAAGCUUGAUCUCCACAGACUCUGGAAUCUCUUCCGUUUUGAUGGUUACCGCUACAUC
AF525216.1 1558 ...............C...................................U...........C.C..................................................U...
DQ238861.1 1561 ...................................................U...........C.C..................................................U...
CAT        1558 ..............G.........................................U........C..........................................C...........
HP         1559 ...............................................................C.C..................................................U...
P2MER      1559 .................U......................................U......C.C..................................................U...
J1710_A    1559 ...............................................................C.C..................................................U...

L13218.1   1678 GACGUGGUCAUAGUGGGCGCGGAUAGAGAUUACGUGUGGCCCUAUCAAAAUGGCGUGUAUGUACACGGCGGCAAGGGACCCAAUGGUACUGGCAACUACGGGAACGCUGACGUUCAUGAC
AF525216.1 1678 ...................UA.G.........U......................................U...........A.......A........A...U...........C...
DQ238861.1 1681 ...................UA.G.........U......................................U...........A.......A........A...U...........C...
CAT        1678 .................U.U....................................A...........U..U...................A............................
HP         1679 ...................U............U......................................U...........A.......A........A...U...........C...
P2MER      1679 ...................U............U......................................U...........A.......A............U...........C...
J1710_A    1679 ...................U............U......................................U...........A......AA........A...U...........C...

L13218.1   1798 GGAAUAGGGACGAUAUUUUCGUCUUUCAACAACAAUGUUAACGUGCAAACGUCCGACCUCAUGCUGGGCCUAUUGACCCUGUGGAACCACAUAACGACUACGUACGCUACUGAGGAGGAG
AF525216.1 1798 .......................C..U..U..............................C....A...U...C............U......G..........................
DQ238861.1 1801 .......................C..U..U..............................C....A...U...C............U......G..........................
CAT        1798 .......................C..U...G......................U........UU.....U..................................................
HP         1799 .......................C..U.................................U....A...U...C..............................................
P2MER      1799 .......................C..U...G.............................U....A...U...C..............................................
JA1710_A   1799 .......................C..U......R..........................U....A...U...C..............................................

L13218.1   1918 GUCACCAUGGCGAUUAAGAUUGCAGCGGCUUUUGCCCUCGUCUACCCAGUACAGCCUAUUGUCUACUCCGGUUGCCCGAAAGCUUUUCAGAGACACACGUCAUACUACCAGCCCUCAUCG
AF525216.1 1918 ..U.................................................................U..C..UU...GC........U.AU...........U.U.............
DQ238861.1 1921 ..U.................................................................U..C..UU...GC........U.AU...........U.U.............
CAT        1918 .......................................................................C.......GG..C........U....................U......
HP         1919 ..U.................................................................U..C...U...GG...C..U.U.AU.............U.............
P2MER      1919 ..U.................................................................U..C.......GG...C..U.U.AU.............U.............
J1710_A    1919 ..U.................................................................U..C...U...GG...C..U.U.AU.............U.............

L13218.1   2038 GAGAACUGCUACGCAACCGACACGGCUGAAGUCAAGAGCGUGUGGGAUACGGUCGAGCUUAGCGUACAGGUUAAUAACGCUAUGGUACUAGGUAUGACACUACCGUUUGGCCAACCUACA
AF525216.1 2038 ............A.......U...........G......AC.....................U...........CG............................................
DQ238861.1 2041 ............A.......U...........G......AC.....................U......C....CG............................................
CAT        2038 ............A.G.....U...........G......AC.....................U.....................................................C...
HP         2039 ............A...................G......AC.....................U...........C.............................................
P2MER      2039 ............A...................G......AC.....................U...........C.............................A...............
J1710_A    2039 ............A...................G......AC.....................U...........C.............................................
```

```
L13218.1   2158 CUUAGCAGUGCACAAUGGUAUAAUAACAUCGAUAAGGCCGAGAUCUCUAUGUUUAAGGUCGGCAAUCUACCGUUACAGAACCUCGAUUAUCUAUCUCUCGAUAUGAUGGAAUUCUACGCU
AF525216.1 2158 G............G.....U..........................................U......................................C..................
DQ238861.1 2161 G............G.....U..........................................U.................G....U...............C..................
CAT        2158 G..................U.........U..C.....U.......................U........AC............C.................................
HP         2159 G..................U..........................................U..Y...................................C..................
P2MER      2159 G..................U..........................................U......................................C..................
J1710_A    2159 G..................U..........................................U..C...................................C..................

L13218.1   2278 CCUACGACAGGACAACUGUACGACAUACGUAGUGAUAACCUGAUCUUGAGCGCCCAUAGGACGGUGAACCUCGGAAUAGGCUACACAGCUCUGGCAGAUUUCUUCGCUUAUCUAGCUUCA
AF525216.1 2278 ........G......U..............................C..........................................C.................C............
DQ238861.1 2281 ........G......U..............................C..........................................CG................C............
CAT        2278 ............................................U...........................................................U...............
HP         2279 ........G............................G........C..........................................C.................C............
P2MER      2279 ........G............................G........C..........................................C.................C............
J1710_A    2279 ........G............................G........C.....................U..A.................Y.................C............

L13218.1   2398 GUUCCUGCCCAGAGUUUUUACCACGACAGGAUGGUCACCUCACCGAUCUCAAAGCAGACGUACUCCGUCUAUGAACGUUUUAUUGAACGUUUUAUUGAUGACUUCGUAGGCUGGGACAGA
AF525216.1 2398 ...............................................U.........G.................................................G.......G....
DQ238861.1 2401 ...............................................U.........G.................................................G.......G....
CAT        2398 .......................U.....................................................C...............G.............G...........G
HP         2399 ........................A.............U........U.........G.......U.........................................G.......G....
P2MER      2399 ........................A.............U........U.........G.......U.........................................G............
J1710_A    2399 ........................A.............U........U.........G.......U.........................................G.......G....

L13218.1   2518 UGUGACCUGUUCAACCUUGAUACAUUGUUAGGUGCAAAACACAUAGCCGGGGUAGCCUCAUCUCCAAUCCCGUGGCACUGCUCGCUACAGAGAUGCCCACUACCCAUAAUCAUGCAUUAC
AF525216.1 2518 ..............................CA..............U.......C.........U..............................U........................
DQ238861.1 2521 ..............................CA..............U.......C.........U..............................UG.......................
CAT        2518 ...............................C...G....G.....................C....................AU.........................U.........
HP         2519 ........................................G......................................................U........................
P2MER      2519 ........................................G.......................U..............................U........................
J1710_A    2519 ........................................G.....U................................................U........................

L13218.1   2638 ACAGGCCUCACUUUCGGCCAGGAGCAUAUAACAGUCAGGGACGUCGCCGGGGUUGAGGGCUUACAGCAGAUCGUCAUGAGGGACUUCCAGGGUCGUAUCGUAGUAGAGCGCUUGGGCACA
AF525216.1 2638 ........ACA.............................................A..............U..GU.....A..GA.......A........C....CGC.........U
DQ238861.1 2641 .......AACA...........................................C.A..............U..GU.....A..GA.......A........C....CGC.........U
CAT        2638 ........ACA.............................................A.................G......A..GA.......AAA...........CGC.........U
HP         2639 ........ACA....................G........................A.................GU.....A..GA.......A........C....CGC.........U
P2MER      2639 ........ACA....................A........................A.................GU.....A..GA.......A........C....CGC.........U
J1710_A    2639 ........ACA....................G........................A.................GU.....A..GA.......A........C....CGC.........U

                                                                        -1 ribosomal frameshift heptamer (Li et al., 2001)
L13218.1   2758 GCUGCACCAUCUAGAAUUGCAGUCAAAUUAGACUGGUCUAGGCUUAGUGCCUGGUACUCAGACACAACCUGCGCCAUCCCUUUAUCCGAUCGUGUGAUGGAGAUCGUCAAUUACGCGGCA
AF525216.1 2758 ...............C........................A......C..............U.....................................................A...
DQ238861.1 2761 .......G.......C........................A....G................U.......................G.............................A...
CAT        2758 ...............C...............................C......................................U..................A..........A...
HP         2759 ..Y............C...........Y...................C..............U..................W...............................U..A...
P2MER      2759 ...............C...............................C..............U..................................................U..A...
J1710_A    2759 ..C............C...........C...................C..............U..................A...............................U..A...
```

```
                            Second cytosine insertion and CP ORF frame shift
L13218.1   2878 AUAUGGGAUCCUACACAGGAAAGACACGC-ACAGGUUUCGUGUACACAUACUUUAGCCCCAAUUUCCUUUCGAGCUUUAACGUGUCUGAGCCUAUAUUCAAUACGAGUAUCAACCUUACA
AF525216.1 2878 ..............G..........G...-................................C....................................C.C.A.GCC............
DQ238861.1 2881 ..............G..........G...-................................C..U.................................C.C.A.GCC............
CAT        2878 ..............G..........GU..C....................................................C..................C..................
HP         2879 ..............G..........G...C................................C......................................C.A..CC............
P2MER      2879 ..............G..........G...C.................G..............C......................................C.A..CC............
J1710_A    2879 ..............G..........G...C................................C......................................C.A..CC............

                      CP ORF stop codon (Wang et al., 1993)
L13218.1   2997 CCACCUUAUGACGACACGAGCCAAGCUGUAAUUCAAAAUUUGUCCAUGCCACAGAUGCUCAGCUUCGAUCCCUACUACGAGUCUACGUUCUAUGUGGUCUCCGCGGAUAACGAGUGGAUA
AF525216.1 2997 ........C..U............A...............................................................................................
DQ238861.1 3000 ....G...C..U............A..............A................................................................................
CAT        2998 .....C..C..U.........C.CA...............................................................................................
HP         2999 ........C..U............A............................................................................................G..
P2MER      2999 ........C..U............A............................................................................................G..
J1710_A    2999 ........C..U............A............................................................................................G..

                                                                                                                   Uracil insertion
                                                                                          Alternative +1/-2 ribosomal frameshift
L13218.1   3117 CCGACUUCAGGGCCUGCUUGGAAGGUACCAUAUCUGGAAAACGUAGUUAAGCGGUCUGGCAGGCGCUUGCUGGCGGAGCUCAGGAUAGCAUCCAAUAACGGGUCUGGAGACCGUACCUU-
AF525216.1 3117 ................................C......................................................................................-
DQ238861.1 3120 ................................C......................................................................................-
CAT        3118 .....C..........................C.....G.............................................................................CUUU
HP         3119 .....C..........................C...................................................................................CUUU
P2MER      3119 .....C..........................C....................................T..................................C...........CUUU
J1710_A    3119 .....C..........................C...................................................................................CUUU

                    New CP ORF stop codon
L13218.1   3236 CUUGACGACGUGUAAGACAAGAAAGGGAAGGCAUUUUACCUACUUCUCUGCUGCUUUGGGGGGCAAGAUCCUGGAAUUCGUAUGCGCUCCACUGUCGAGUAUCAGUCUCCAGGGCGGGCA
AF525216.1 3236 ........................A..........A.G....U.............................................................................
DQ238861.1 3239 ........................A..........A.G....U.............................................................................
CAT        3238 CUUG........UAA....................A.G....U....................................A........................................
HP         3239 CUUG........UAA.........A..........A.G....U..................................................R..........................
P2MER      3239 CUUG........UAA.........A........C.A.G....U.............................................................................
J1710_A    3239 CUUG........UAA.........A..........A.G....U..................................................A..........................

L13218.1   3356 AACGAUCUACGCUCCGAUUCAGCUCCAAGACGUCAUACCAGUACGUAAAGAGGAUCCAGUGCCAGGGAGCAUAUAUGCAGUCUUUAAGUUCUUCUCAGAACCAAAGGCCUGGGAGGCACG
AF525216.1 3356 ................................C..............G..A.......................C..............U.................U......AU...
DQ238861.1 3359 ................................C..............G..A.......................C..............U.................U......AU...
CAT        3358 ...A............................C........C.....G...........................C........C...C.U...........G.................
HP         3359 ................................C..............G..A.......................C..............U.................U......A....
P2MER      3359 ................................C..................A............A..........C..............U.................U...........
J1710_A    3359 ................................C..............G..A.......................C.....U........U.................U......A....

L13218.1   3476 UGCGCUUAAGUCCUACAAAGUCAGGUUCCAAGAUCUCCCAAGCCACAUAGUGAUUAGUGAGUUGAAAGAGAGAGCGGCGAGAAGCUAUAUCGGGUCGAGGGGUUAUGUCGAUACAGGUUU
AF525216.1 3476 ...A.................U.......................................C.A....................U...................................
DQ238861.1 3479 ...A.................U.......................................C.A....................U...................................
CAT        3478 ....UC.................A................................A................................................C..............
HP         3479 ...A.................U.......................................C.A....................U...................................
P2MER      3479 .....................U.......................................C.A....................U...................................
J1710_A    3479 ...A.................U.......................................C.A....................U...................................
```

```
L13218.1   3596 UAAGGCUUUGGACAUUUAUAUAGAUAUUCUUUCCCAAAUGGAGCUGCCAAAGUACAUACACGAGUUCUUAAUUUUACUCAGAGGCAAGGUGUGUGAAGUCUCGAGGUUGUACAAGAAGGA
AF525216.1 3596 ......................................................................G............................U....................
DQ238861.1 3599 .....G............................................................A...G............................U....................
CAT        3598 .................................U.........U....................................AG.................U.............G......
HP         3599 ......................................................................G............................U....................
P2MER      3599 ......................................................................G..C.........................U....................
J1710_A    3599 ......................................................................G............................U....................

L13218.1   3716 ACAGGUAUUCGUAAUAUUACUAACCGUAUUCUCUGAGCUCACAGCCAUAGUCCGCCAUAGAGGUAACAAGUCCACGGGGUCGAUGGGGAGGAUGUGGACUUUGUUAUCAGACUUCGAGAC
AF525216.1 3716 ..........A.....C..................................U....................................................................
DQ238861.1 3719 ..........A.....C..................................U............................................U.......................
CAT        3718 ...................................................U.....C......................................................C....A..
HP         3719 ................C..................................U....................................................................
P2MER      3719 ................C..................................U....................................................................
J1710_A    3719 ................C..................................U....................................................................

L13218.1   3836 CUUGCUUGGGAAGGUGAGCUAUAAGAACCCGAGUAUCAUUGAGGAGCAGGUUGUUCCCUGGCUAACCUCAGACCCUAUACCACGUACCCCUGACUUCUACUCUACAUAUUUCAAGACGGC
AF525216.1 3836 .C......................................................................U....................U...........G..........U...
DQ238861.1 3839 .C...........................G..........................................U....................U...........G..........U...
CAT        3838 .C.............A.........................................................................................G..............
HP         3839 .C......................................................................U....................U...........G..............
P2MER      3839 .C.................................................C....................U....................U...........G..............
J1710_A    3839 .C......................................................................U....................U...........G..............

L13218.1   3956 UGUCCAGUUUAUGCACAGGACAUUCGUUCCCGUCACUCUCAGAAGUGCCCCUCCGUUAACAUUUCACGAGUACUGUGGGAGGCCGGAGCUCUGGGGAACCACGGGAUCCGGCUACAUCGG
AF525216.1 3956 G..U..........................U.................................U............C....U.....................................
DQ238861.1 3959 G..U..............C...........U.................................U............C....U.....................................
CAT        3958 G.............................U....U............U..............CU...........................................U........U..
HP         3959 G..U..........................U.................................U............C..........................................
P2MER      3959 G..U..........................U.................................U............C..........................................
J1710_A    3959 G..U..........................U.................................U............C..........................................

L13218.1   4076 CUAUGGCAAGCGUAGUUUUAACAAAUGGUCGAUCUACGGAGCUUAUCCUACCGAGGAGAUUUAUCGGCUAGCACUCUAUGGAGAUAAUCCACCCCUCAAGCCGCUGGAAAAACCCGAACU
AF525216.1 4076 ............C.....C.....................................................................................................
DQ238861.1 4079 ............C.....C.................................................C...................................................
CAT        4078 ............C.....C..........................CU..G...........................................U..........................
HP         4079 ............C.....C.....G....................CU..G...........................................U..........................
P2MER      4079 ...C........C.....C.....G....................CU..G...........................................U..........................
J1710_A    4079 ............C.....C.....G....................CU..G...R.......................................U..........................

L13218.1   4196 UACGAAGGUACGUGCUGUAAUCUCCGCCUCCCUCCAAUCGUAUAUAUUGAUGUCUUACCUCGAGUACAUCAUGGCAGACACCAUAGUUGACAAAGCAUUCACUACAACUCUGAUGAACGA
AF525216.1 4196 ..............................UU..........C...........C...........................U.....................................
DQ238861.1 4199 ..............................UU..........C...........C...........................U.....................................
CAT        4198 ..............................UU...............................A..............................A.........................
HP         4199 ..............................UU..........C...........C.................................................................
P2MER      4199 ..............................UU..........C...........C.................................................................
J1710_A    4199 ..............................UU..........C...........C.................................................................
```

```
L13218.1   4316 CAGGCAGCUAGAGAACCUUGAGCGCCACAUGAUGACCAUGACCGGAGGAGUCAGGGUUCCAGUGGACCAGUCCAAUUUUGAUCGCCAGCCUGACUUGGUCCAAAUAGGCAUCUGGCAACA
AF525216.1 4316 .......U............................................................................U...................................
DQ238861.1 4319 .......UAU..............................................................G...........U...................................
CAT        4318 ........................................................................................................................
HP         4319 .......U................Y.................................................................C.............................
P2MER      4319 .......U..........C.......................................................................C.............................
J1710_A    4319 .......U................U.................................................................C.............................

L13218.1   4436 GUUACUGUUUCACCUGGCAUCUGCGUCAGCUCCGUACAGAGCCCGCGACAGUGUCUCAUUAGUCAUAUCACGUUUAGCUUCAACUACGACUUUCCCAAACCUAAAGGUACGGAUGUCGGA
AF525216.1 4436 ......A........A..............C........G..................C...................C...........C.....G.......................
DQ238861.1 4439 ......A........A..............C........G..................C...................C...........C.....G.......................
CAT        4438 .........C.....A.....C............C...AG..................C..................U.........A..C.....G........A..............
HP         4439 ...............A..............C........G..................C...................C...........C.....G.......................
P2MER      4439 ...............A..............C........G..................C...................C...........C.....G.......................
J1710_A    4439 ...............A........K.....C........G..................C...................C...........C.....G.......................

L13218.1   4556 CGGUGACAAACGUGUGCUACACGGCCUCCCCUCGGGGUGGAAGUGGACCGCUCUGCUAGGGGCUCUCAUCAAUGUGACACAACUUUUAACCAUGGCGGAAUUGUCCAACACUCUGGCCUC
AF525216.1 4556 ......U.........................................U........G...........................C..................................
DQ238861.1 4559 ......U............G............................U........G...........................C..................................
CAT        4558 ...................................................A............................................U.......................
HP         4559 ......U..............................................................................C..........A.......................
P2MER      4559 ......U..............T...............................................................C..........A.......................
J1710_A    4559 ......U..............................................................................C..........A.......................

L13218.1   4676 GUUGAGGUCAACGGUGGUGCAGGGCGACGAUAUCGCUCUAAGUAUGACUGACCGGGAGCAGGCCACACAGUUAGUUGACACUUAUGCACGUCAGGGGUUCGAGGUCAACCCGAAGAAGUU
AF525216.1 4676 ..................A..........................................................................A..........................
DQ238861.1 4679 ..................A..........................................................................A..C..G....................
CAT        4678 ..................A.............................................G.......................................................
HP         4679 ..................A.............................................G............................A..........................
P2MER      4679 ..................A.....A.......................................G............................A..........................
J1710_A    4679 ..................A.............................................G............................A..........................

L13218.1   4796 CUGGAUAUCACCCGACCGGGACGAAUUCCUUCGGAGGGUAGCAACCCCUGGUAUAGUCGCGGGGUACCCAGCUAGAAUGAUGAUUAAAUUGUUAUAUCAGCUGACGGAACCUGAAGAGCC
AF525216.1 4796 ..............................C.....................................................................U...................
DQ238861.1 4799 ..............................C.....................................................................U...................
CAT        4798 ..............................C.............................A.........................................A.................
HP         4799 ..............................C.........................................................................................
P2MER      4799 ..............................C.........................................................................................
J1710_A    4799 ..............................C.........................................................................................

L13218.1   4916 CUCACACUACAUCUCCAUGUUGCCAAAGUUAGCGAAGGUGCCUAACGUGGUCCAGGAGCAUGUAAAGCCCCGGUCUGAUGUGCCAUGGUCGGAGACCGUCGAAGAACUUGAUUGGCGCGA
AF525216.1 4916 ............................C.................A......G....U......GC.....................................................
DQ238861.1 4919 ............................C.................A......G....U......GC.....................................................
CAT        4918 U................C..........C.G...........................U......GC.....................................................
HP         4919 ............................C.................A......G....U......GC.....................................................
P2MER      4919 ............................C.................A......G....U......GC.....................................................
J1710_A    4919 ............................C.................A......G....U......GC.....................................................
```

```
L13218.1   5036 AGCUCUCGCUAUACUGAGGCAUAGGCCUGCCCGUACGUCAGAACUGGUGACUCAAUGGCUGCAACUGAUUGGUAGGUUUACAGCUGCUCAUCCAGAUAAGCGAGCCUUAACUCUUCUCUA
AF525216.1 5036 ....................................A........................................................U.......................U..
DQ238861.1 5039 ....................................A........................................................U.......................U..
CAT        5038 .......................................................................................C........C....................U..
HP         5039 .............................................................................................U..C....................U..
P2MER      5039 ..............................U..............................................................U..C....................U..
J1710_A    5039 ..................R..........................................................................U..C....................U..

L13218.1   5156 CCGUUGGUUCGUGAGAGAUUUGACACAUGCUACAAAAAUAAAAAAAAGAAAUCUUCUGGUCCUGUUACAGCAGCCGGGCUUCUGGGGGGGUUAUUCCCAGAGCCUGGUUGGCUUGUUAAG
AF525216.1 5156 ..A...............C.....G..............................................................A................................
DQ238861.1 5159 ..A...............C.....G..............................................................A................................
CAT        5158 .......................................................................................A................................
HP         5159 ..A.......................................................A............................A................................
P2MER      5159 ..A.......................................................A............................A................................
J1710_A    5159 ..A.......................................................A............................A................................

L13218.1   5276 GACCACUCACCUCGCAGAUAUGAUAGUUAGCGAACUAGACAUAGGACUGCCAGGUCCUCGGGCCACGUCUAGUCGCUUUGGGACAUCCCCUACCUCACUGGCCCCGCACUACCUCACUCU
AF525216.1 5276 .......................................................................A.....................U..........................
DQ238861.1 5279 .......................................................................A.....................U..........................
CAT        5278 .............................................................................................U..G.......................
HP         5279 ......................................G......................................................U..........................
P2MER      5279 ......................................G......................................................U..........................
J1710_A    5279 ......................................G......................................................U..........................

L13218.1   5396 GAUAGUUCCGAGUAGCGUGCAUGUGCAGUCGCAAAAGGAAUUGGAUUUGUGGGGGCUGUGCAGAUCCAGUAAAUAUGCUAAGCACUAUUCAAACAUAUUUCGAUACUACAAACUGACUCU
AF525216.1 5396 ........................................C..............................U................................................
DQ238861.1 5399 ........................................C..............................U................................................
CAT        5398 ......................................................C.......A........C................................................
HP         5399 ........................................C..............................U................................................
P2MER      5399 ........................................C..............................C................................................
J1710_A    5399 ........................................C..............................U................................................

L13218.1   5516 UCCUACGUUGGUGCUUUGGGCCCAACGGCUAGGUGAUAAACACGUCACCGAUUUCAUUAGAUCUGUCACCCUGGGUUCAGAAAUACCAAAAUAUGACCCGCAUGCGCGUCUAUUCACAAG
AF525216.1 5516 .......................................................................................................A................
DQ238861.1 5519 .......................................................................................................A................
CAT        5518 C..................................................C...................C...............................A................
HP         5519 .......................................................................................................A................
P2MER      5519 .......................................................................................................A................
J1710_A    5519 .......................................................R...............................................A................

L13218.1   5636 UAACGGGGUGUCAGUAGGAAUGUUAAUAAGGAUAAGAGUACGACAUUUUACCCACAGAGUGCUUCGAAGAGAGACACCUAAGUGUAUCCCCGUAAUCGUCGGGCUAUAUCGGGAGCAGAC
AF525216.1 5636 ....................C...................................................................................................
DQ238861.1 5639 ....................C.......................................................................................C...........
CAT        5638 ....................C...................................................................................................
HP         5639 ....................C...................................................................................................
P2MER      5639 ....................C...................................................................................................
J1710_A    5639 ....................C...................................................................................................
```

```
L13218.1   5756 ACUAUCCGUUCCGGAGAGUGUUCGUCUAUCGGAACCUGACAAAAUACUAUUAAGCCUACAACGGGCGUCGGGUUACUCACUGAAUCUAGUUAAGAAGAUAUUACAGAUCACUAGGAAACC
AF525216.1 5756 ..........................................G.......C................................G....................................
DQ238861.1 5759 ..........................................G.......C................................G....................................
CAT        5758 ..........................................G.......C................................G..................G.................
HP         5759 ..........................................G.......C................................G....................................
P2MER      5759 ..........................................G.......C................................G....................................
J1710_A    5759 ..........................................G.......C................................G....................................

L13218.1   5876 AGUGGACCAUCCGGUGGACGCGAGGUUCUCACAGGCAUUCCCUAACAACUGGAGCGACUUGGCUAGAACGGCUGGCACCUUUUUGUUGACUGUACCGGCUUAGGAGGUUUCAGGAGACAA
AF525216.1 5876 .......................................................................................A................................
DQ238861.1 5879 .......................................................................................A..............A.................
CAT        5878 ......U...........U..............................................................................-......................
HP         5879 .......................................................................................A.........-......................
P2MER      5879 ......U................................................................................A.........-......................
J1710_A    5879 .......................................................................................A.........-......................

                                                                                                                          GA(AA)deletion
L13218.1   5996 UGCUGACCUUGUGCCUGAGCGUUUGGGCUUCAACCAUAGCAGCUGGCUUCACGCAAUUUUUAGGUCAAGGAAAUUUCUCCUUUGCGUAGUAGCAUAGCUGUUCAAAGAAAGAAAAAGAAA
AF525216.1 5996 ....AG......CG.............................C.................................................G......C.........AG....----
DQ238861.1 5999 ....AG......CG.............................C.....G...................................C.......G......C.........AG....----
CAT        5997 .....G......CG.............................C........................................................C.........AG....--AC
HP         5998 .....G......CG.............................C.................................................G......C.........AG....----
P2MER      5998 .....G......CG.............................C.................................................G......C.........AG....----
J1710_A    5998 .....G......CG.............................C.................................................G......C.........AG....----

L13218.1   6116 UACUGGUAAUUACCGCACACGAUCGGUGGGUCGACCAGCAUAGGGUGGUGUGCGGUUCGAGAACGGGGCUGGCCUUAGUACCGUCAUAACACCUGGACACGUGUAGAAACUGUCCCCCCU
AF525216.1 6112 ....A........U...................G......................C..........A....................................U...............
DQ238861.1 6115 ....A........U...................G........C.............C..........A.............G.......G.......................G......
CAT        6115 ....A............................G.......................U................C.............................................
HP         6114 ....A........U...................G......................................................................................
P2MER      6114 ....A........U...................G.......................U..............................................................
J1710_A    6114 ....A........U...................G......................................................................................

L13218.1   6236 GUAGACUCCGUUGCCUGUAGGAGGCAGCGUACGAGGGGGUCG
AF525216.1 6232 .............G............................
DQ238861.1 6235 ..........................................
CAT        6235 ..........................................
HP         6234 ..........................................
P2MER      6234 ..........................................
J1710_A    6234 ..........................................
```
