# Supplementary material for: Re-Discovery of Giardiavirus: Genomic and Functional Analysis of Viruses from Giardia duodenalis Isolates
Source: Biomedicines. 2021 Jun 8;9(6):654. doi: 10.3390/biomedicines9060654 (PMC8230311; doi:10.3390/biomedicines9060654)
Supplement: Supplementary file 1 [file biomedicines-09-00654-s001.zip › Supplemental Files/Figure S3.pdf]

A

```
CP_HP                                        LAPENITFDTLNTQNDHEETYGESPEVPKASIAPAGRQNVPVLQQNQENEDNHALGGSEDAKDEREIQS
CP_J1710_A                                   ........H............................................................
CP_P2MER                                     ...................................................D................F
CP_CAT        MVWGTGYGNPSPLNPYGFASLHRGGLNPLI-.......L........Y...H............................K.................RF
CP_L13218     MVWGTGYGNPSPLNPYGFASLHRGGLNPLI-.............H......H..............................................RF
CP_AF52521    MVWGTGYGNTSPLNPYGFASLHRGGLNPLI-.............................................K......................F
CP_DQ23886    MVWGTGYGNTSPLNPYGFASLHRGGLNPLIF.............................................K......................F

CP_HP         SAIKTLYNYYSEGPSTPIMPHLVNRLRGLDALAKVDATLSKVDMNAAYIFALRPTFPYSYGYKQRFSNRRLTTSALCYARTGLSSFLTVDKTYTSNSPL
CP_J1710_A    ...........................................................................L.......................
CP_P2MER      ...........................................................................L.......................
CP_CAT        ..................................I....T...................................V.............N.........
CP_L13218     ..................................I....T...................................L.......................
CP_AF52521    ...............I...........................................................L.......................
CP_DQ23886    ...............I.............V....................P...............T....S...L.......................

CP_HP         KGGSRGWPIFN[VL]GVSPHVAEPHMRTLSPIGLEVFNLATSQFSKTLLTASSKVFTQSLYTADILSIFGEVFLPHVMQPVSNYTPILVRALLALIHILGSG
CP_J1710_A    ...........[V-].......................................................................................
CP_P2MER      R..........[V-]..............................................S........................................
CP_CAT        ...........[V-].....................................................................................P.
CP_L13218     ...........[V-]D..S.................................................................................P.
CP_AF52521    ...........[-I].....................................................................................P.
CP_DQ23886    ...........[-I].....................................................................P...............P.

CP_HP         SGNCSLSSSIFESSIPQFLTISHSTNMSNRTRYCLHTWSAYKDMFRNGIPPQSTFPPTLAPEGSSARILIPAALVTSPMFPWLLVLVSSGPQFFLYSKDA
CP_J1710_A    ....................................................................................L...............
CP_P2MER      ....................................................................................................
CP_CAT        ..................F.V.................................L............V................A...............
CP_L13218     ....................V................R................L............V...E............I...............
CP_AF52521    ....................................................................................................
CP_DQ23886    ..............................................I....H................................................

CP_HP         SINTVDIGSRGRITSPIPDVAHLDLHRLWNLFRFDGYRYIDVVIVGVDRDYVWPYQNGVYVHGGKGPKGTDNYENADVHDGIGTIFSSFNN[N-]VNVQT
CP_J1710_A    ......................................................................N....................[ND].....
CP_P2MER      .........................................................................G................D[N-].....
CP_CAT        .....................N..............H..............................N.....G................D[N-].....
CP_L13218     .....................K........................A....................N..G..G.................[N-].....
CP_AF52521    .....H...........S.............................G............................................[N-].....
CP_DQ23886    .................S.............................G............................................[N-].....
```

```
CP_HP       SDLLLGLSTLWNHITTTYATEEEVTMAIKIAAAFALVYPVQPIVYSGCSRALY[NS]HTSYFQPSSENCYTTDTAEVKSTWDTVELSVQVNNAMVLGMTLPF
CP_J1710_A  .....................................................[N-].............................................
CP_P2MER    ................................................P....[N-].............................................
CP_CAT      ...I...L........................................P..FQ[-S]....Y........................................
CP_L13218   ...M...L........................................PK.FQ[-R]....Y........A........V......................
CP_AF52521  ..............A..................................S.FH[N-]..................................D..........
CP_DQ23886  ..............A..................................S.FH[N-]................................L.D..........

CP_HP       GQPTVSSAQWFNNIDKAEISMFKVGNLPLQNLDYLSLDMMEFYAPTTGQLYDIRSDSLISSAHRTVNLGIGYTALADFFAYLASVPAQSFYHNRMVTSPI
CP_J1710_A  ....................................................................................................
CP_P2MER    ....................................................................................................
CP_CAT      ........................................................N..L................................D.......
CP_L13218   ....L.....Y.............................................N..L................................D.......
CP_AF52521  ........R...............................................N...................................D.......
CP_DQ23886  ........R.....................K.V.......................N.................V.................D.......

CP_HP       SKQAYSVYERFIERFIDDFVGW[GD]RCDLFNLDTLLGAKRIAGVASSPIPWHCSLQRCPLPIIMHYTGLHFGQEHIRVRDVAGVEGLQQIVLRNDQGSIVL
CP_J1710_A  ......................[G-]................V...........................................................
CP_P2MER    ......................[-D]......................L............................K........................
CP_CAT      ...T...........V......[-D]...........A.......................................T..............M.....K..V
CP_L13218   ...T..................[-D]..............H.............................T......T..............M.DF..R..V
CP_AF52521  ......................[G-]...........H..H.V..P..L............................T........................
CP_DQ23886  ......................[G-]...........H..H.V..P..L..........A.........Q.......T.......Q................

CP_HP       DALGTAAPSRLAVKLDWSRLSAWYSDTTCAIP[IL]SDRVMEIVNYAAIWDPTQERRATGFVYTYFSPNFLSSFNVSEPIFNKTINLTPPYDDTSQTVIQNL
CP_J1710_A  ................................[I-]..................................................................
CP_P2MER    ................................[-L]..................................................................
CP_CAT      ................................[-L].......IT.............................A......S...........H......
CP_L13218   ER........I.....................[-L]....................H.QVSCTHTLAPISFRALTCLSLYSIRVSTLHHLMTTRAKL
CP_AF52521  ..................K.............[-L]......................QVSCTHTLAPTSFRALTCLSLYSTRPSTLHHLTMTRAKL
CP_DQ23886  .......R..........K.G...........[-L]......................QVSCTHTLAPTFFRALTCLSLYSTRPSTLHHV

CP_HP       SMPQMLSFDPYYESTFYVSADNEWVPTSGPAWKVPYLENVVKRSGRRLLAELRIASNNGSGDRTFLDDV
CP_J1710_A  .....................................................................
CP_P2MER    .....................................................................
CP_CAT      ........................I............................................
```
