# Supplementary material for: Re-Discovery of Giardiavirus: Genomic and Functional Analysis of Viruses from Giardia duodenalis Isolates
Source: Biomedicines. 2021 Jun 8;9(6):654. doi: 10.3390/biomedicines9060654 (PMC8230311; doi:10.3390/biomedicines9060654)
Supplement: Supplementary file 1 [file biomedicines-09-00654-s001.zip › Supplemental Files/Figure S4.pdf]

```
RdRp_HP                                                 LTTCKTRKGRHYAYFSAALGGKILEFVCAPLSSISLQGGQTIYAPIQLQDAIPVRR
RdRp_JA17                                               ........................................................
RdRp_P2MER                                              .......................................................K
RdRp_CAT                                                ..........................I..........................A..
RdRp_L1321  NEWIPTSGPAWKVPYLENVVKRSGRRLLAELRIASNNGSGDRTF...........FT.....................................V....K
RdRp_AF525  NEWIPTSGPAWKVPYLENVVKRSGRRLLAELRIASNNGSGDRTF........................................................
RdRp_DQ238  NEWIPTSGPAWKVPYLENVVKRSGRRLLAELRIASNNGSGDRTF........................................................

RdRp_HP     EDPVPGSIYAVFKFFSEPKAWETRALKSYKVRFQDLPSHIVISELKERAARSYIGSRGYVDTGFKALDIYIDILSQMELPKYIHEFLVLLRGKVCEVSR
RdRp_JA17   ...................................................................................................
RdRp_P2MER  .....R................A............................................................................
RdRp_CAT    .............L........A..S.....K..........N............................................I..K........
RdRp_L1321  ......................A................................................................I...........
RdRp_AF525  ......................I............................................................................
RdRp_DQ238  ......................I..........................................G...................L.............

                                                            1
RdRp_HP     LYKKEQVFVILLTVFSELTAIVRHRGNKSTGSMGRMWTLLSDFETLLGKVSYKNPSIIEEQVVPWLTSDPIPRTPDFYSTYFKTAVQFMHRTFVPVTLRS
RdRp_JA17   ....................................................................................................
RdRp_P2MER  ....................................................................................................
RdRp_CAT    ..R.......................................L.....................................................I...
RdRp_L1321  ....................................................................................................
RdRp_AF525  ........I..........................................................................M................
RdRp_DQ238  ........I...........................C.................R............................M......S.........

                               2                                                    3
RdRp_HP     APPLTFYEYCARPELWGTTGSGYIGYGKRSFNKWSIYGAYSA[E-]EIYRLALYGDNPPLKPLEKPELTKVRAVISASFQSYILMSYLEYIMADTIVDKAFT
RdRp_JA17   ..........................................[EG]........................................................
RdRp_P2MER  ..........................................[E-]........................................................
RdRp_CAT    ..........G...............................[E-].....................................................T..
RdRp_L1321  ......H...G.............................PT[E-]................................L.......................
RdRp_AF525  ............S...........................PT[E-].................................................L......
RdRp_DQ238  ............S...........................PT[E-]....-............................................L......

                                           4
RdRp_HP     TTLMNDRQLENLERHMMTMTGGVRVPVDQSNFDRQPDLVQIGIWQQLLFHLASASAPYRARDSVSLVISRLASTTTFPNLKVRMSDGDKRVLHGLPSGWK
RdRp_JA17   ....................................................................................................
RdRp_P2MER  ....................................................................................................
RdRp_CAT    .........................................................HK............V............................
RdRp_L1321  ....................................................................................................
RdRp_AF525  ....................................................................................................
RdRp_DQ238  ........Y...................................................................................D.......

                  5                            6                                                7       8
RdRp_HP     WTALLGALINVTQLLTMAELSNTLASLRSTVVQGDDIALSMTDREQAAQLVDTYARQGFEVNPKKFWISPDRDEFLRRVATPGIVAGYPARMMIKLLYQL
RdRp_JA17   ....................................................................................................
RdRp_P2MER  ....................................................................................................
```

```
RdRp_CAT     ....................................................................................................
RdRp_L1321   ...............................................T....................................................
RdRp_AF525   ...............................................T....................................................
RdRp_DQ238   ...............................................T..........L.........................................

RdRp_HP      TEPEEPSHYISMLPKLAKVPNMVREYVSPRSDVPWSETVEELDWREALAILRHRPARTSELVTQWLQLIGRFTAAHPDKRALTLLYHWFVRDLTHATKI
RdRp_JA17    ...................................................................................................
RdRp_P2MER   ...................................................................................................
RdRp_CAT     ...........T.........V.Q..............................................................R............
RdRp_L1321   .....................V.Q.H.K..........................................................R............
RdRp_AF525   ...................................................................................................
RdRp_DQ238   ...................................................................................................

RdRp_HP      KKRNLLILLQQPGFWGGYSQSLVGLLRTTHLADMIVSELGIGLPGPRATSSRFGTSPTSLAPHYLTLIVPSSVHVQSQKELDLWGLCRSSIYAKHYSNI
RdRp_JA17    ...................................................................................................
RdRp_P2MER   ..........................................................................................T........
RdRp_CAT     ......V................................D...............................................K..T........
RdRp_L1321   ......V................................D..................................................K........
RdRp_AF525   ......V................................D..........N.......................................I........
RdRp_DQ238   ......V................................D..........N.......................................I........

RdRp_HP      FRYYKLTLPTLVLWAQRLGDKHVTDF[I-]RSVTLGSEIPKYDPHTRLFTSNGVSVGTLIRIRVRHFTHRVLRRETPKCIPVIVGLYREQTLSVPESVRLSE
RdRp_JA17    ..........................[IV].........................................................................
RdRp_P2MER   ..........................[I-].........................................................................
RdRp_CAT     ..........................[I-].....P...................................................................
RdRp_L1321   ..........................[I-]................A...........M............................................
RdRp_AF525   ..........................[I-].........................................................................
RdRp_DQ238   ..........................[I-].........................................................................

RdRp_HP      PDKILSSLQRASGYSLSLVKKILQITRKPVDHPVDARFSQAFPNNWSDLARTAGTFLLTVPLRRFQETMLALSLSVWASTIAAGFTQFLGQGNFSFA
RdRp_JA17    .................................................................................................
RdRp_P2MER   .................................................................................................
RdRp_CAT     .................................................................................................
RdRp_L1321   .....L..........N............................................A
RdRp_AF525   .............................................................A
RdRp_DQ238   ............................................................
```
