# Supplementary material for: Re-Discovery of Giardiavirus: Genomic and Functional Analysis of Viruses from Giardia duodenalis Isolates
Source: Biomedicines. 2021 Jun 8;9(6):654. doi: 10.3390/biomedicines9060654 (PMC8230311; doi:10.3390/biomedicines9060654)
Supplement: Supplementary file 1 [file biomedicines-09-00654-s001.zip › Supplemental Files/Figure S6.pdf]

A)

```
>5′-UTR_HP (120-501nt)
GGACUUCGCCACGCUUACACCAUCGAGAACACAUAAGUGGUGUAAUAUUAUGUGUUUGGCUGCGUGUGUCACGAGCACGAUCCAUGGGGGAAUCUAACCC
....[[[[[[[[.((((((((((((.((((((((((]]]]]]].]]..))))))))))((((.((((({{{{...))))).....(((([[[[[[[[.)))
AGGUGGUGUUGUGUAUGAUAAGGUACAAGCCCGUCAACACCACAGGUGGAGAGGGACAGGAGUCCCAUUUCCCCUCUAGAAAACAGCAGUGGUCCGCCCC
).(((((((((([[[.}}}}..(((]]].)))...)))))))))(((]]..]](((((....)))))..]]]])))..........[[[[[[.[[.[[[[[
UCAUGCCCUAUGAAACGUUAGGGGGCCGACACUAGUGGGCAAGCCCGAUGGUGUGGGGGACUGGAUAUGGGAACACCAGUCCGUUAAACCCGUACGGCUU
[[[(((((..]]........]]]]]].]]]]]].]])))))))))))))))))))))(((((((...........)))))))......((((...((((.
CGCCAGCCUACACCGGGGGGGGCUUAAUCCCCUUAUUCUUGGCACCUGAAAAUAUUACUUUUGACACUUUGAACACACAGAA
.[[[)))).....))))(((((.....)))))........]]].......................................
```

B)

```
>5′-UTR_CAT (120-500nt)
AGACUUCGCCACGCUUACACCAUCGAGAACACAUAGGUGGUGUAAUAUUAUGUGUUUGGCUGCGUGUGUCACGAGCACGAUCUAUGGGGGAAUCUAACCC
........[[{{..[[[[[[[[[[..((((((((((]]]]]]]]]...))))))))).{{{{[[[[(((((.(((]]]].....(({{{{{{{{{.[[.)
AGGUGGUGUUGUGUAUGAUGAGGUACAAGCCCGUCAACACCACAGGUGGAGAGGGACAGGAGUCCCAUUUCCCCCUUUAAAAACAGCAGUGGUCCGCCCC
).(((((((((((((..[[[.[[))))..]]]]]]))))))))((]]]]}}}(((((....)))))..}}}}}})).......}}}}[[[[.[[.[[[[[
UCAUGCCCUAUGAAACGUUAGGGGGCCGACACUAGUGGGCAAGCCCGAUGGUGUGGGGGACUGGAUAUGGGAACCCCAGUCCGUUAAACCCGUACGGCUU
[.[[((((........]]..]]]]]].]]]]]].}}))))(([[[[{{[[[[[[[[[(((((((.[[[[[[[[.))))))))]]]...]]]]]][[[[))
CGCCAGCCUACACCGGGGGGGGCUUAAUCCCCUUAUUUUGGCACCUGAAAACAUUACUCUUGACACUUUAAAUACACAGAA
.]]].]]]]]]]]]]](((((((.....))))))...}}.]]]..............))).)))))................
```

C)

```
>5′-UTR_ GLV-L13218.1 (120-500nt)
AGACUUCGCCACGCUUACACCAUUGAGAACACAUAGGUGGUGUAAUAUUAUGUGUUUGGCUGCGUGUGUCACGAGCACGAUCUAUGGGGGAAUCUAACCC
........((....[[[[[[[[[[..((((((((((]]]]]]]]]...)))))))))))[[[[[[[[((((.((]]]]].....(([[[[[.[[[.[[[)
AGGUGGUGUUGUGUAUGAUGAGGUACAAGCCCGUCAACACCACAGGUGGAGAGGGACAGGAGUCUCAUUUCCCCUCCAAAAAACAGCAGUGGUCCGCCCC
).(((((((((((((..[[[.[[))))..]]]]]])))))))).]]]]]](((((((....))))).))]]]]].........]]]..(((.((.(((((
UCAUGCCCUAUGAAACAUUAGGGGGCCGACACUAGUGGGCAAGCCCGAUGGUGUGGGGGACUGGAUAUGGGAACCCCAGUCCGUUAAACCCGUACGGCUU
(.((([[[[[.....)))]]))))))).)))))((.((({{{{{{))).))((((((((((((((....]]][[[.)))))))]]].[[[[[[....}}}}
CGCCAGCCUACACCGGGGGGGGCUUAAUCCCCUUAUUUUGGCACCUGAAAAUAUUACUUUUGACACUUUAAACACACACAA
.}}{{.))))))))]]]](((((.....)))))....]]}}................)).))))................
```
