# Supplementary material for: Re-Discovery of Giardiavirus: Genomic and Functional Analysis of Viruses from Giardia duodenalis Isolates
Source: Biomedicines. 2021 Jun 8;9(6):654. doi: 10.3390/biomedicines9060654 (PMC8230311; doi:10.3390/biomedicines9060654)

A

>CP_HP1 (96% sequence coverage combined from trypsin and chymotrypsin digestion)

```
         10         20         30         40         50         60         70         80         90        100
LAPENITFDT LNTQNDHEET YGESPEVPKA SIAPAGRQNV PVLQQNQENE DNHALGGSED AKDEREIQSS AIKTLYNYYS EGPSTPIMPH LVNRLRGLDA LAKVDATLSK
VDMNAAYIFA LRPTFPYSYG YKQRFSNRRL TTSALCYART GLSSFLTVDK TYTSNSPLKG GSRGWPIFN[V/L180]GVSPHVAEPH MRTLSPIGLE VFNLATSQFS
KTLLTASSKV FTQSLYTADI LSIFGEVFLP HVMQPVSNYT PILVRALLAL IHILGSGSGN CSLSSSIFES SIPQFLTISH STNMSNRTRY CLHTWSAYKD MFRNGIPPQS
TFPPTLAPEG SSARILIPAA LVTSPMFPWL LVLVSSGPQF FLYSKDASIN TVDIGSRGRI TSPIPDVAHL DLHRLWNLFR FDGYRYIDVV IVGVDRDYVW PYQNGVYVHG
GKGPKGTDNY ENADVHDGIG TIFSSFNNNV NVQTSDLLLG LSTLWNHITT TYATEEEVTM AIKIAAAFAL VYPVQPIVYS GCSRALY[N/S518]HT SYFQPSSENC
YTTDTAEVKS TWDTVELSVQ VNNAMVLGMT LPFGQPTVSS AQWFNNIDKA EISMFKVGNL PLQNLDYLSL DMMEFYAPTT GQLYDIRSDS LISSAHRTVN LGIGYTALAD
FFAYLASVPA QSFYHNRMVT SPISKQAYSV YERFIERFID DFVGW[G/D686]RCDL FNLDTLLGAK RIAGVASSPI PWHCSLQRCP LPIIMHYTGL HFGQEHIRVR
DVAGVEGLQQ IVLRNDQGSI VLDALGTAAP SRLAVKLDWS RLSAWYSDTT CAIP[I/L794]SDRVM EIVNYAAIWD PTQERRATGF VYTYFSPNFL SSFNVSEPIF
NKTINLTPPY DDTSQTVIQN LSMPQMLSFD PYYESTFYVV SADNEWVPTS GPAWKVPYLE NVVKRSGRRL LAELRIASNN GSGDRTFLDDV
```

>CP_CAT (93% sequence coverage combined from trypsin and chymotrypsin digestion)

```
         10         20         30         40         50         60         70         80         90        100
MVWGTGYGNP SPLNPYGFAS LHRGGLNPLI LAPENITLDT LNTQNDYEET HGESPEVPKA SIAPAGRQNV PVLQQNQENK DNHALGGSED AKDEREIRFS AIKTLYNYYS
EGPSTPIMPH LVNRLRGLDA LAKIDATLTK VDMNAAYIFA LRPTFPYSYG YKQRFSNRRL TTSAVCYART GLSSFLTVNK TYTSNSPLKG GSRGWPIFNV GVSPHVAEPH
MRTLSPIGLE VFSQNLATFS KTLLTASSKV FTQSLYTADI LSIFGEVFLP HVMQPVSNYT PILVRALLAL IHILGPGSGN CSLSSSIFES SIPQFFTVSH STNMSNRTRY
CLHTWSAYKD MFRNGIPPQS TLPPTLAPEG SSARVLIPAA LVTSPMFPWL LALVSSGPQF FLYSKDASIN TVDIGSRGRI TSPIPDVANL DLHRLWNLFR FDGHRYIDVV
IVGVDRDYVW PYQNGVYVHG GKGPNGTDNY GNADVHDGIG TIFSSFNDNV NVQTSDLILG LLTLWNHITT TYATEEEVTM AIKIAAAFAL VYPVQPIVYS GCPRAFQSHT
SYYQPSSENC YTTDTAEVKS TWDTVELSVQ VNNAMVLGMT LPFGQPTVSS AQWFNNIDKA EISMFKVGNL PLQNLDYLSL DMMEFYAPTT GQLYDIRSDN LILSAHRTVN
LGIGYTALAD FFAYLASVPA QSFYHDRMVT SPISKQTYSV YERFIERFVD DFVGWDRCDL FNLDTLLAAK RIAGVASSPI PWHCSLQRCP LPIIMHYTGL HFGQEHITVR
DVAGVEGLQQ IVMRNDQGKI VVDALGTAAP SRLAVKLDWS RLSAWYSDTT CAIPLSDRVM EIINYAAIWD PTQERRATGF VYTYFSPNFL SSFNASEPIF NTSINLTPPY
DDTSHTVIQN LSMPQMLSFD PYYESTFYVV SADNEWIPTS GPAWKVPYLE NVVKRSGRRL LAELRIASNN GSGDRTFLDD V
```

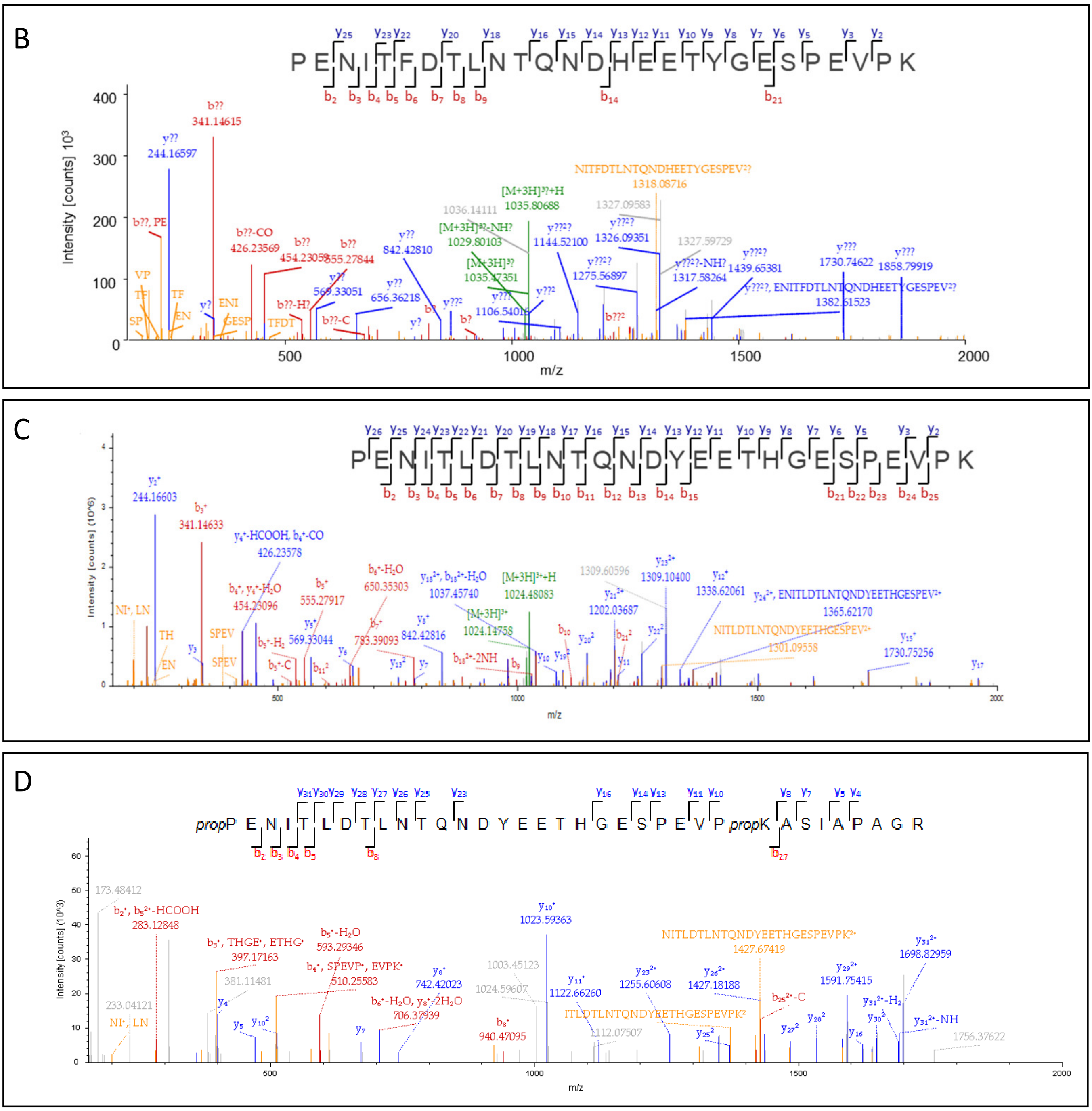

Supplement: Supplementary file 1 [file biomedicines-09-00654-s001.zip › Supplemental Files/Figure S7.pdf]
