# Supplementary material for: Re-Discovery of Giardiavirus: Genomic and Functional Analysis of Viruses from Giardia duodenalis Isolates
Source: Biomedicines. 2021 Jun 8;9(6):654. doi: 10.3390/biomedicines9060654 (PMC8230311; doi:10.3390/biomedicines9060654)
Supplement: Supplementary file 1 [file biomedicines-09-00654-s001.zip › Supplemental Files/Figure S8.pdf]

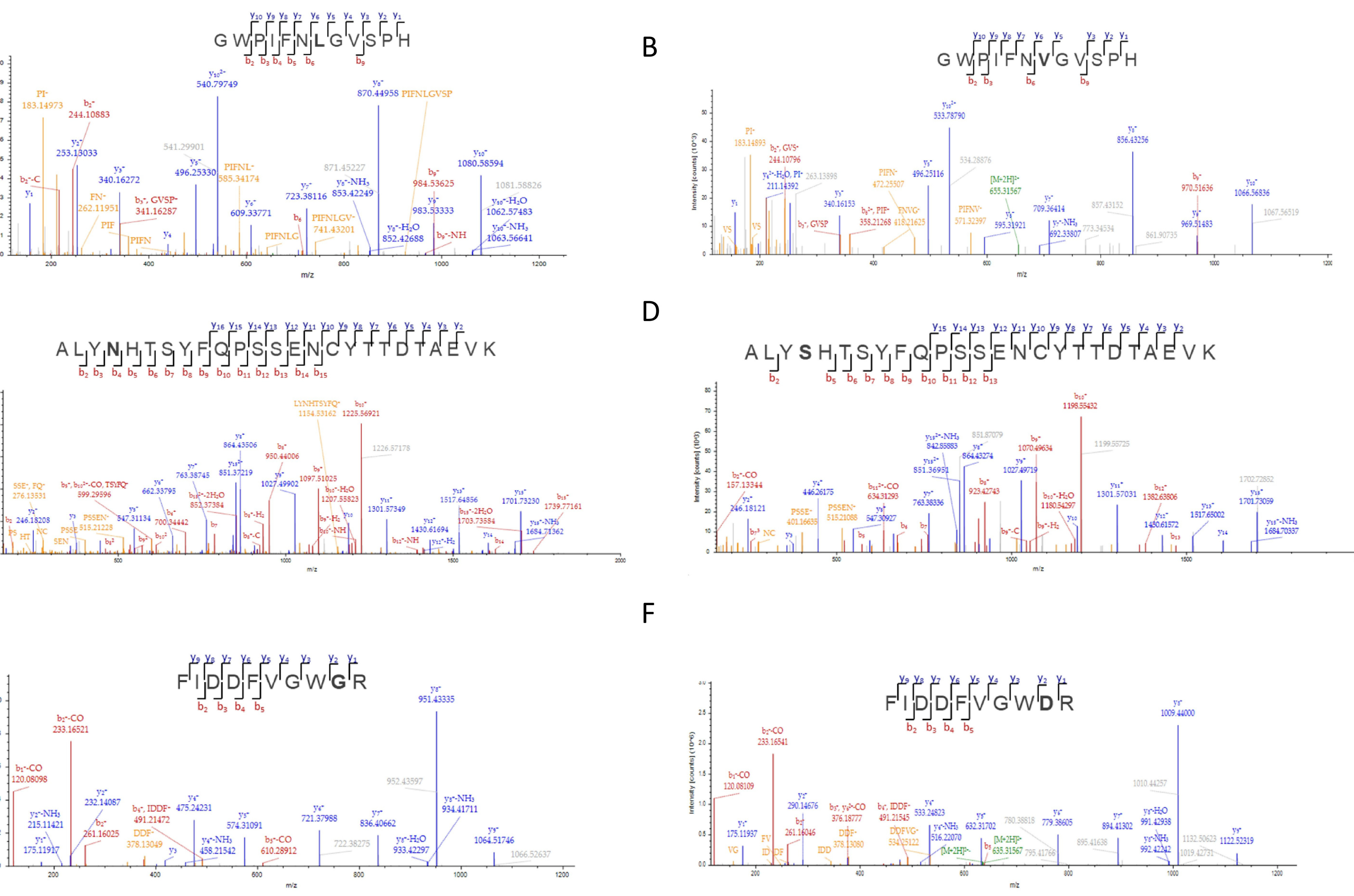

B
D
F
GWPIFNLGVSPH
GWPIFNVGVSPH
ALYNHTSYFQPSSENCYTTDTAEVK
ALYSHTSYFQPSSENCYTTDTAEVK
FIDDFVGWGR
FIDDFVGWDR
m/z
Intensity [counts] (10^3)
Intensity [counts] (10^6)
