# Supplementary material for: Re-Discovery of Giardiavirus: Genomic and Functional Analysis of Viruses from Giardia duodenalis Isolates
Source: Biomedicines. 2021 Jun 8;9(6):654. doi: 10.3390/biomedicines9060654 (PMC8230311; doi:10.3390/biomedicines9060654)
Supplement: Supplementary file 1 [file biomedicines-09-00654-s001.zip › Supplemental Files/Figure S9.pdf]

A

>CP_P2MER (55% sequence coverage from trypsin digestion)

```
LAPENITFDT LNTQNDHEET YGESPEVPKA SIAPAGRQNV PVLQQNQENE DDHALGGSED AKDEREIQFS AIKTLYNYYS EGPSTPIMPH LVNRLRGLDA 100
LAKVDATLSK VDMNAAYIFA LRPTFPYSYG YKQRFSNRRL TTSALCYART GLSSFLTVDK TYTSNSPLRG GSRGWPIFNV GVSPHVAEPH MRTLSPIGLE 200
VFNLATSQFS KTLLTASSKV FTQSLYSADI LSIFGEVFLP HVMQPVSNYT PILVRALLAL IHILGSGSGN CSLSSSIFES SIPQFLTISH STNMSNRTRY 300
CLHTWSAYKD MFRNGIPPQS TFPPTLAPEG SSARILIPAA LVTSPMFPWL LVLVSSGPQF FLYSKDASSN TVDIGSRGRI TSPIPDVAHL DLHRLWNLFR 400
FDGYRYIDVV IVGVDRDYVW PYQNGVYVHG GKGPKGTDNY [G/E]NADVHDGIG TIFSSFNDNV NVQTSDLLLG LSTLWNHITT TYATEEEVTM AIKIAAAFAL 500
VYPVQPIVYS GCPRALYNHT SYFQPSSENC YTTDTAEVKS TWDTVELSVQ VNNAMVLGMT LPFGQPTVSS LPFGQPTVSS EISMFKVGNL PLQNLDYLSL 600
DMMEFYAPTT GQLYDIRSDS LISSAHRTVN LGIGYTALAD FFAYLASVPA QSFYHNRMVT SPISKQAYSV SPISKQAYSV DFVGWDRCDL FNLDTLLGAK 700
RIAGVASSLI PWHCSLQRCP LPIIMHYTGL HFGQEHIKVR DVAGVEGLQQ IVLRNDQGSI VLDALGTAAP VLDALGTAAP RLSAWYSDTT CAIPLSDRVM 800
EIVNYAAIWD PTQERRATGF VYTYFSPNFL SSFNVSEPIF NKTINLTPPY DDTSQTVIQN LSMPQMLSFD LSMPQMLSFD SADNEWVPTS GPAWKVPYLE 900
NVVKRSGRRL LAELRIASNN GSGDRTFLDD V 931
```

B

>CP/RdRp_HP (19% sequence coverage combined from trypsin and AspN digestion)

```
LAPENITFDT LNTQNDHEET YGESPEVPKA SIAPAGRQNV PVLQQNQENE DNHALGGSED AKDEREIQSS AIKTLYNYYS EGPSTPIMPH LVNRLRGLDA 100
LAKVDATLSK VDMNAAYIFA LRPTFPYSYG YKQRFSNRRL DNHALGGSED GLSSFLTVDK TYTSNSPLKG GSRGWPIFN[V/L] GVSPHVAEPH MRTLSPIGLE 200
VFNLATSQFS KTLLTASSKV FTQSLYTADI LSIFGEVFLP TTSALCYART PILVRALLAL IHILGSGSGN CSLSSSIFES SIPQFLTISH STNMSNRTRY 300
CLHTWSAYKD MFRNGIPPQS TFPPTLAPEG SSARILIPAA GLSSFLTVDK LVLVSSGPQF FLYSKDASIN TVDIGSRGRI TSPIPDVAHL DLHRLWNLFR 400
FDGYRYIDVV IVGVDRDYVW PYQNGVYVHG GKGPKGTDNY HVMQPVSNYT TIFSSFNNNV NVQTSDLLLG LSTLWNHITT TYATEEEVTM AIKIAAAFAL 500
VYPVQPIVYS GCSRALYNHT SYFQPSSENC YTTDTAEVKS PILVRALLAL VNNAMVLGMT LPFGQPTVSS AQWFNNIDKA EISMFKVGNL PLQNLDYLSL 600
DMMEFYAPTT GQLYDIRSDS LISSAHRTVN LGIGYTALAD LVTSPMFPWL QSFYHNRMVT SPISKQAYSV YERFIERFID DFVGWDRCDL FNLDTLLGAK 700
RIAGVASSPI PWHCSLQRCP LPIIMHYTGL HFGQEHIRVR LVLVSSGPQF IVLRNDQGSI VLDALGTAAP SRLAVKLDWS RLSAWYSDTT CAIP[I/L]SDRVM 800
EIVNYAAIWD PTQERRATGF VYTYFSPNFL SSFNVSEPIF ENADVHDGIG DDTSQTVIQN LSMPQMLSFD PYYESTFYVV SADNEWVPTS GPAWKVPYLE 900
NVVKRSGRRL LAELRIASNN GSGDRTFLTT CKTRKGRHYA TIFSSFNNNV LEFVCAPLSS ISLQGGQTIY APIQLQDAIP VRREDPVPGS IYAVFKFFSE 1000
PKAWETRALK SYKVRFQDLP SHIVISELKE RAARSYIGSR TWDTVELSVQ DIYIDILSQM ELPKYIHEFL VLLRGKVCEV SRLYKKEQVF VILLTVFSEL 1100
TAIVRHRGNK STGSMGRMWT LLSDFETLLG KVSYKNPSII VNNAMVLGMT DPIPRTPDFY STYFKTAVQF MHRTFVPVTL RSAPPLTFYE YCARPELWGT 1200
TGSGYIGYGK RSFNKWSIYG AYSAEEIYRL ALYGDNPPLK FFAYLASVPA RAVISASFQS YILMSYLEYI MADTIVDKAF TTTLMNDRQL ENLERHMMTM 1300
TGGVRVPVDQ SNFDRQPDLV QIGIWQQLLF HLASASAPYR QSFYHNRMVT RLASTTTFPN LKVRMSDGDK RVLHGLPSGW KWTALLGALI NVTQLLTMAE 1400
LSNTLASLRS TVVQGDDIAL SMTDREQAAQ LVDTYARQGF DVAGVEGLQQ PDRDEFLRRV ATPGIVAGYP ARMMIKLLYQ LTEPEEPSHY ISM[L/F]PKLAKV 1500
PNMVREYVSP RSDVPWSETV EELDWREALA ILRHRPARTS IVLRNDQGSI GRFTAAHPDK RALTLLYHWF VRDLTHATKI KKRNLLILLQ QPGFWGGY[S/P]Q 1600
SLVGLLRTTH LADMIVSELG IGLPGPRATS SRFGTSPTSL NKTINLTPPY SSVHVQSQKE LDLWGLCRSS IYAKHYSNIF RYYKLTLPTL [V/M]LWAQRLGDK 1700
HVTDFIRSVT LGSEIPKYDP HTRLFTSNGV SVGTLIRIRV DDTSQTVIQN ETPKCIPVIV GLYREQTLSV PESVRLSEPD KILSSLQRAS GYSLSLVKKI 1800
LQITRKPVDH PVDARFSQAF PNNWSDLART AGTFLLTVPL YFSAALGGKI SLSVWASTIA AGFTQFLGQG NFSFA 1873
```

>CP/RdRp_CAT (42% sequence coverage combined from trypsin and AspN digestion)

```
MVWGTGYGNP SPLNPYGFAS LHRGGLNPLI LAPENITLDT LNTQNDYEET HGESPEVPKA SIAPAGRQNV PVLQQNQENK DNHALGGSED AKDEREIRFS 100
AIKTLYNYYS EGPSTPIMPH LVNRLRGLDA LAKIDATLTK VDMNAAYIFA LRPTFPYSYG YKQRFSNRRL TTSAVCYART GLSSFLTVNK TYTSNSPLKG 200
GSRGWPIFNV GVSPHVAEPH MRTLSPIGLE VFNLATSQFS KTLLTASSKV FTQSLYTADI LSIFGEVFLP HVMQPVSNYT PILVRALLAL IHILGPGSGN 300
CSLSSSIFES SIPQFFTVSH STNMSNRTRY CLHTWSAYKD MFRNGIPPQS TLPPTLAPEG SSARVLIPAA LVTSPMFPWL LALVSSGPQF FLYSKDASIN 400
TVDIGSRGRI TSPIPDVANL DLHRLWNLFR FDGHRYIDVV IVGVDRDYVW PYQNGVYVHG GKGPNGTDNY GNADVHDGIG TIFSSFNDNV NVQTSDLILG 500
LLTLWNHITT TYATEEEVTM AIKIAAAFAL VYPVQPIVYS GCPRAFQSHT SYYQPSSENC YTTDTAEVKS TWDTVELSVQ VNNAMVLGMT LPFGQPTVSS 600
AQWFNNIDKA EISMFKVGNL PLQNLDYLSL DMMEFYAPTT GQLYDIRSDN LILSAHRTVN LGIGYTALAD FFAYLASVPA QSFYHDRMVT SPISKQTYSV 700
YERFIERFVD DFVGWDRCDL FNLDTLLAAK RIAGVASSPI PWHCSLQRCP LPIIMHYTGL HFGQEHITVR DVAGVEGLQQ IVMRNDQGKI VVDALGTAAP 800
SRLAVKLDWS RLSAWYSDTT CAIPLSDRVM EIINYAAIWD PTQERRATGF VYTYFSPNFL SSFNASEPIF NTSINLTPPY DDTSYTVIQN LSMPQMLSFD 900
PYYESTFYVV SADNEWIPTS GPAWKVPYLE NVVKRSGRRL LAELRIASNN GSGDRTFLTT CKTRKGRHYA YFSAALGGKI LEFICAPLSS ISLQGGQTIY 1000
APIQ[L/F]QDAIP ARREDPVPGS IYAVFKLFSE PKAWEARASK SYKVKFQDLP SHIVINELKE RAARSYIGSR GYVDTGFKAL DIYIDILSQM ELPKYIHEFL 1100
ILLKGKVCEV SRLYRKEQVF VILLTVFSEL TAIVRHRGNK STGSMGRMWT LLSDLETLLG KVSYKNPSII EEQVVPWLTS DPIPRTPDFY STYFKTAVQF 1200
MHRTFVPVIL RSAPPLTF[H/Y]E YCGRPELWGT TGSGYIGYGK RSFNKWSIYG AYSAEEIYRL ALYGDNPPLK PLEKPELTKV RAVISASFQS YILMSYLEYI 1300
MADTIVDKTF TTTLMNDRQL ENLERHMMTM TGGVRVPVDQ SNFDRQPDLV QIGIWQQLLF HLASASAPHK ARDSVSLVIS RLVSTTTFPN LKVRMSDGDK 1400
RVLHGLPSGW KWTALLGALI NVTQLLTMAE LSNTLASLRS TVVQGDDIAL SMTDREQAAQ LVDTYARQGF EVNPKKFWIS PDRDEFLRRV ATPGIVAGYP 1500
ARMMIKLLYQ LTEPEEPSHY ISTLPKLAKV PNVVQEYVSP RSDVPWSETV EELDWREALA ILRHRPARTS ELVTQWLQLI GRFTAA[H/Y]PDK RALTLLYRWF 1600
VRDLTHATKI KKRNLLVLLQ QPGFWGGYSQ SLVGLLRTTH LADMIVSELD IGLPGPRATS SRFGTSPTSL APHYLTLIVP SSVHVQSQKE LDLWGLCKSS 1700
TYAKHYSNIF RYYKLTLPTL VLWAQRLGDK HVTDFIRSVT PGSEIPKYDP HTRLFTSNGV SVGTLIRIRV RHFTHRVLRR ETPKCIPVIV GLYREQTLSV 1800
PESVRLSEPD KILSSLQRAS GYSLSLVKKI LQITRKPVDH PVDARFSQAF PNNWSDLART AGTFLLTVPL RRFQETMLAL SLSVWASTIA AGFTQFLGQGNFSFA 1904
```
