# Supplementary material for: Re-Discovery of Giardiavirus: Genomic and Functional Analysis of Viruses from Giardia duodenalis Isolates
Source: Biomedicines. 2021 Jun 8;9(6):654. doi: 10.3390/biomedicines9060654 (PMC8230311; doi:10.3390/biomedicines9060654)
Supplement: Supplementary file 1 [file biomedicines-09-00654-s001.zip › Supplemental Files/Supplemental Figures Captions and Tables.pdf]

Supplemental Figure Captions

Figure S1 Multiple sequence alignment of GLV genomes. The consensus genome sequences of the sequenced GLV strains (HP; CAT; P2MER and J10/17_A) were aligned with the GLV genomes available in GenBank (L13218.1; AF525216.1; DQ238861.1) using Clustal X software. Sequence gaps are grey boxed. Nucleotide insertion (compared to reference sequences) in the HP, CAT, P2MER and J10/17_A are black boxed, whereas heterogeneous nucleotide positions are in bold and white boxed. Heterogeneous position are indicated following the IUPAC nomenclature. Relevant stop codons (red) are marked and self-explained. The original -1 ribosomal frameshift heptamer (green) and the proposed +1/-2 ribosomal frameshift sequence (pink) are marked.

Figure S2. Phylogenetic analysis GLV strains. A) Left, unrooted cladogram of the viral genomes obtained from G. duodenalis isolates. Bootstrap values are indicated next to each branch point. Right, pairwise comparison table of the viral genomes obtained from *G. duodenalis* isolates. Number of different nucleotides and percentage of identity are showed. Lower values are highlighted in dark violet, higher values are highlighted in dark red. B) Left, unrooted cladogram of the deduced amino acidic sequences of the viral capsid protein (ORF1); right, corresponding pairwise comparison table. C) Left, unrooted cladogram of the deduced amino acidic sequences of the viral RNA-dependent RNA polymerase (ORF2); right, corresponding pairwise comparison table. Bootstrap values in the cladograms are indicated next to each branch point. In pairwise comparison tables,number of different aminoacids and percentage of identity are showed. Lower values are highlighted in dark violet, higher values are highlighted in dark red.

Figure S3. Multiple alignment of the CP (ORF1) aminoacid sequences. Light blue arrow indicate the portion of the protein used for 3D structure prediction, and red portion indicate region higher sequence divergence between CAT and HP (see Figure 3). Dark grey boxed sequence were omitted for the CP phylogenetic analysis (see Figure 3). Alternative aminoacid positions, as inferred from sequencing of the viral genomes, are in square brackets [] and black boxed when confirmed by mass spectrometry (see Figure 3). Note that residues Ile and Leu cannot be distinguished from each other in mass spectrometry

Figure S4. Multiple alignment of the RdRp (ORF2) aminoacid sequences. Only the protein region encoded after the proposed ribosomal frameshift site were aligned. Alternative aminoacid positions, as inferred from sequencing of the viral genomes, are light grey boxed and in square brackets [ ]. Conserved motifs within the RDRP sequences [46] are black boxed. Dark grey boxed sequence were omitted for the RdRp phylogenetic analysis (see Figure 3). Alternative aminoacid positions, as inferred from sequencing of the viral genomes, are in square brackets [ ] and black boxed when confirmed by mass spectrometry (see Figure 3). Note that residues Ile and Leu cannot be distinguished from each other in mass spectrometry.

Figure S5. Circular tree graphical view. BLAST pairwise alignment between the JA1710_B putative viral RNA-directed RNA-polymerase domain (yellow highlighted sequence in Figure 1 panel C) and the non-redundant protein sequence database (setting: Tree method is neighbor joining; Max Seq Difference is 0.85; Evolutionary distance model is Grishin). Sequence are labelled according to the common names (as reported in Supplemental Excel File 1).

Figure S6 Pseudoknots prediction in the GLV 5′-UTR. Dot-bracket representation (Vienna format) of the maximum expected accuracy (MEA) of RNA structure and pseudoknots prediction by IPknot web server (Version 1.3.1) of the 5′-UTR region (nucleotide 120-501) of GLV_{HP}, GLV_{CAT} and reference GLV L13218.1 genome by IPknot web server (Version 1.3.1) (Scoring model: CONTRAfold model; With refining parameters; Weight for true base pairs: Level 1: 2; Level 2: 16; Level 3: 16) and used for the force directed graph layout of Figure 2.

Figure S7. Mass spectrometry analysis of CP proteins of GLV_{HP} and GLV_{CAT} strain. A) Deduced aminoacid sequences, as inferred from sequencing of the viral genomes, are shown. Sequences represented in the peptides

recovered from LC MS/MS after trypsin or chymotrypsin cleavage of viral proteins are indicated (yellow boxed). Alternative aminoacid positions are in square brackets [ ] and grey boxed when confirmed by mass spectrometry. Note that residues Ile and Leu cannot be distinguished from each other in mass spectrometry. B) MS/MS spectrum of the triple charged CP GLV_{HP} peptide (3-29) derived from tryptic digestion. The ion at m/z 1035.47 was fragmented and b- and y-series ions are shown in red and blue, respectively. Neutral losses from the precursor ion are shown as green peaks and internal fragments are reported in yellow. The sequence of the peptide matching the fragments detected in the MS/MS spectrum is reported in the panel: the dark lines define the fragments of the b- or y- series ion detected in the MS/MS spectrum. C) MS/MS spectrum of the triple charged CP GLV_{CAT} peptide (33-59) derived from digestion with trypsin. The ion at m/z 1024.48 was fragmented and b- and y-series ions are shown in red and blue, respectively. Neutral losses from the precursor ion are shown as green peaks and internal fragments are reported in yellow. The sequence of the peptide matching the fragments detected in the MS/MS spectrum is reported in the panel: the dark lines define the fragments of the b- or y- series ion detected in the MS/MS spectrum. D) MS/MS spectrum of the triple charged CP GLV_{CAT} peptide (33-67) derived from propionylation and tryptic digestion. The precursor ion at m/z 1302.63 was fragmented and b- and y-series ions are shown in red and blue, respectively. The MS/MS spectrum is assigned to the peptide 33-67 propionylated at the N-terminus and at lysine 59.

Figure S8. Mass spectrometry analysis of heterologous position of the GLV_{HP} CP. A) and B) MS/MS spectrum of the triple charged CP- GLV_{HP} peptide (174-185) derived from tryptic digestion. Two ions at m/z 662.35 and 655.34 (A and B, respectively) were detected and fragmented: b- and y-series ions are shown in red and blue, respectively. The masses and fragmentation patterns allow to localize a Leu (A) and a Val (B) in position 180. C) and D) MS/MS spectrum of the double charged CP-HP peptide (515-539) derived from tryptic digestion. Two ions at m/z 976.44 and 967.43 (A and B, respectively) were detected and fragmented: b- and y-series ions are shown in red and blue, respectively. The masses and fragmentation patterns allow to localize an AspN (A) and a Ser (B) in position 518. E) and F) MS/MS spectrum of the double charged CP-HP peptide (678-686) derived from tryptic digestion. Two ions at m/z 606.30 and 635.30 (A and B, respectively) were detected and fragmented: b- and y-series ions are shown in red and blue, respectively. The masses and fragmentation patterns allow to localize a Gly (A) and an Asp (B) in position 686. In all panels, precursor ions and neutral loss from them are shown as green peaks and internal fragments are reported in yellow. The sequence of the peptide matching the fragments detected in the MS/MS spectrum is reported in the panel.

Figure S9 Mass spectrometry analysis of CP from GLV_{P2MER} (A) and CP/RdRp proteins GLV_{HP} and GLV_{CAT} (B) strains. Precursor sequences, as inferred from sequencing of the viral genomes, are shown. Sequences represented in the peptides recovered from LC MS/MS after trypsin or chymotrypsin cleavage of viral proteins are indicated (yellow boxed). Alternative aminoacid positions are in square brackets []. Note that residues Ile and Leu cannot be distinguished from each other in mass spectrometry. Phenylalanine and Leucine residue at the junction between the CP and RdRp are black boxed.

Figure S10 Virtual modelling of GLV_{HP} and GLV_{CAT} CP monomer A) Representation of the capsid of Saccharomyces cerevisiae virus L-A (ScV-L-A), (pdb: 1m1c; monomer A light grey, monomer B dark grey). The model of CAT monomer (magenta) is superimposed to monomer A of ScV-L-A to identify the surface exposed outside the capsid. B) Surface representation of CAT, same view as a. The regions that differ significantly in sequence between CAT and HP are colored green. C) Superposition of the models CAT (magenta, green) and HP-NG (with Asn_{518} and Gly_{686}) (cyan). The dashed circle highlights the exposed fragment 543-553. D and E) Electrostatic surface potential of CAT and HP-NG.

Supplemental Tables

Table S1. RNAseq sequencing results

	Yield (Mbases)	% >= Q30	Mean Quality
HP	9.023	94,89	39,09
CAT	8.035	94,36	38,93

P2MER	8.533	94,8	39,08
J1710	6.683	91,57	38,18

Table S2. Primer used for amplification and Sanger sequencing

Name	5′ -> 3′ sequence	Product Size (bp)
Primers used for PCR and sequencing of JA1710_B isolate		
GLV-JAC7-R*	GCCTGTCCCGCGGGTGCTCCG	-
GLV-JAC7-F	AGAAGGTCTTCGGGCCACTGAGGGG	1026
GLV-JAC7-R8	CGAAGCCACTCCTGAAGGAGAGC	
GLV-JAC7-F7	GCTCTCCTTCAGGAGTGGCTTCG	982
GLV-JAC7-R7	CTGGGCGAAGAGCCAGGC	
GLV-JAC7-AgeF	GAGGGTGTCACCGGTGCGGCGTTC	1788
GLV-JAC7-TestF	GGGGAGCTTGTACTTGGTGC	
GLV-JAC7-NaeF	GCCTCACTTGCAGCCGGCGTGCGCC	1103
GLV-JAC7-F3	CTTGAGGCCCTTGGTGGG	
GLV-JAC7-F6	ACACTCTCCGCGACGTGAG	1428
GLV-JAC7-F4	AGATGACTTCCTCAAGCCC	
GLV-JAC7-F5	GTACCGCTTGACCAGGGCG	399
GLV-JAC7-intR	CGGGGTGGAGGGCGGGCTGCG	
Primers used for PCR and sequencing of HP, CAT, P2MER and JA1710_A isolates		
GLV-Pst-R*	CGACCCCCTCGTACGCTGCCTCCTACAG	-
GLV-Sma-F	GGAAGGAGTGCCAGGCCATTACC	3247
GLV-Sac-R	CACGTCGTCAAGAAGGTACGGTC	
GLV-RecCp-F	GCACCTGAAAATATTACTTTTG	822
GLV-RecCp-R	CGATGAAAGTGAGCAATTACC	
GLV-C-F	GCCAGGATCTGGTAATTGCT	1077
GLV-seqPCR2-R	CTCAAGATCAGGTTATCACTAC	
GLV-Sac-F	CTTACACCACCTTATGACGACACGAG	2600
GLV-Psp-R	CCCAGGGTGACAGATCTAATGAAATC	
GLV-PCR3-F1	GTCACTCTCAGAAGTGCCCCTC	649
GLV-PCR3-R2	GTGTCACATTGATGAGAGCCCC	
GLV-PCR3-R1	GAGGGGCACTTCTGAGAGTGAC	sequencing only
GLV-PCR3-F2	GGGGCTCTCATCAATGTGACAC	sequencing only
GLV-Psp-F	GATTTGTGGGGGCTGTGCAG	sequencing only

* used as primer for the reverse transcription reaction
**size estimated on GLV reference isolate sequence (L13218.1)

Table S3. Primers list for antigens amplification

Primer	Sequence (5′-3′)	Primer features	Localization on GLV reference (L13218.1)	Localization on J17/10_B sequence
**GLV-CP-NT_F**	*ggatcc*GCACCTGAAAATATTACTTTTG	*ggatcc BamHI* restriction site	460-481	
**GLV-CP-NT_R**	*gaattc*CTACGATGAAAGTGAGCAATTACC	*gaattc EcoRI* restriction site	1261-1281	

**GLV-CP-CT_ F**	*ggatcc*ACACCACCTTACGATGACACG	*ggatcc BamHI* restriction site	2294-3014	
**GLV-CP-CT_R**	*gtcgac*TTACACGTCGTCAAGAAAGGTAC	*ctgcag PstI* restriction site	3229-3250	
**J1710_B-NT_F**	*ggatcc*CCACCGCGCCAGCCACTG	*ggatcc BamHI* restriction site		264-281
**J1710_B-NT_R**	*gtcgac***tca**GACGGGGGCGGAGAGCCG	*ctgcag PstI* restriction site; **tca** stop codon		504-521

Table S4. Amount of reads mapping on reference viral genome

Sample	Total reads	Mapped reads	Mapped reads (%)
HP	59,752,148	47,567,247	79.61
CAT	53,209,120	45,416,124	85.35
P2MER	56,509,112	42,600,291	75.39
JA1710	44,256,076	326,624	0.74
