# Supplementary figures and images for: Re-Discovery of Giardiavirus: Genomic and Functional Analysis of Viruses from Giardia duodenalis Isolates
Source: Biomedicines. 2021 Jun 8;9(6):654. doi: 10.3390/biomedicines9060654 (PMC8230311; doi:10.3390/biomedicines9060654)

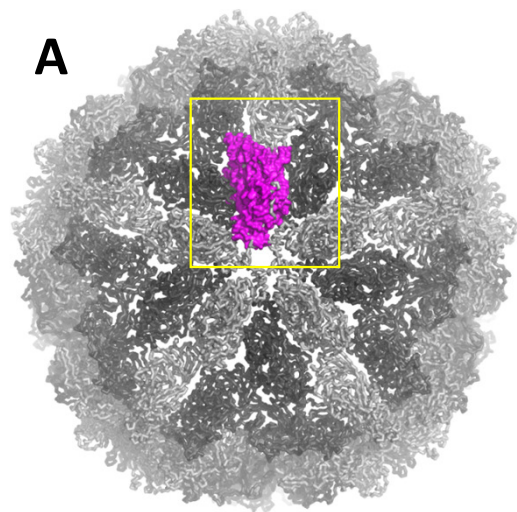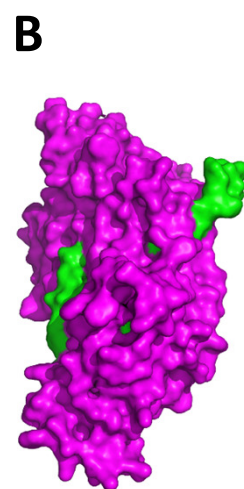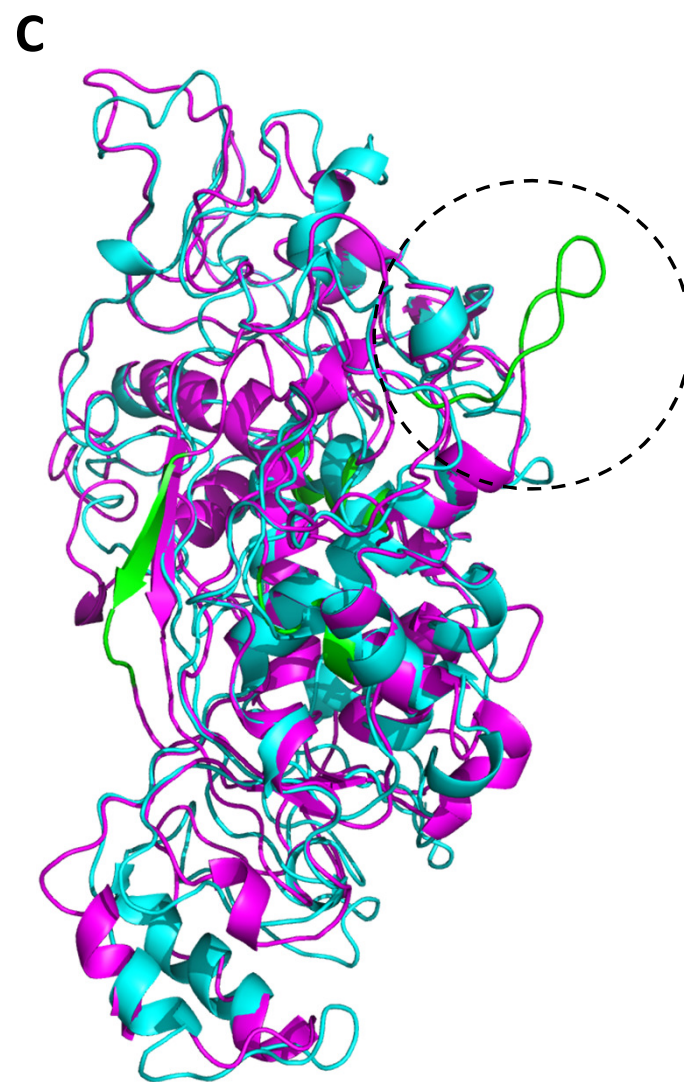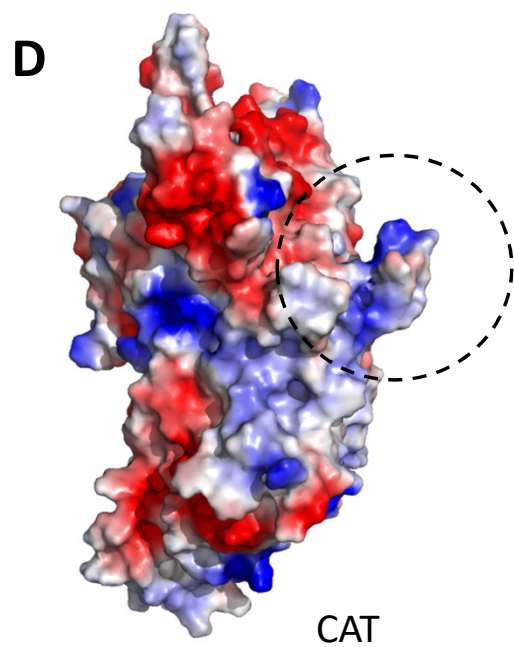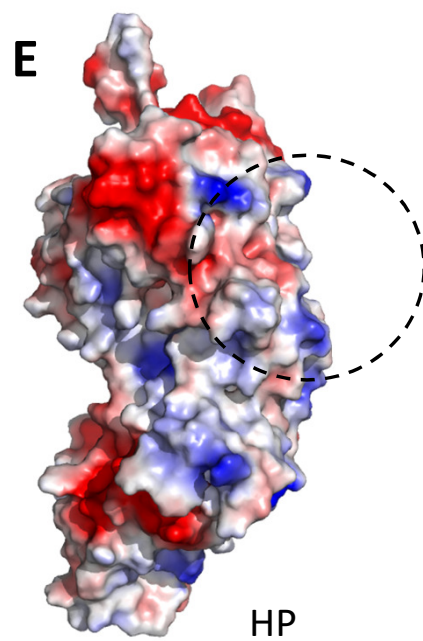

Supplement: Supplementary file 1 [file biomedicines-09-00654-s001.zip › Supplemental Files/Figure S10.pdf]

A

|            |   | 1     | 2     | 3     | 4     | 5     | 6     | 7   |
|------------|---|-------|-------|-------|-------|-------|-------|-----|
| HP         | 1 |       | 7     | 40    | 233   | 256   | 87    | 127 |
| J17/10_A   | 2 | 99,89 |       | 58    | 250   | 272   | 102   | 142 |
| P2MER      | 3 | 99,36 | 99,08 |       | 250   | 280   | 122   | 162 |
| CAT        | 4 | 96,29 | 96,02 | 96,02 |       | 258   | 279   | 320 |
| L13218.1   | 5 | 95,92 | 95,67 | 95,54 | 95,89 |       | 275   | 315 |
| AF525216.1 | 6 | 98,61 | 98,37 | 98,06 | 95,56 | 95,62 |       | 48  |
| DQ238861.1 | 7 | 97,98 | 97,74 | 97,42 | 94,90 | 94,98 | 99,24 |     |

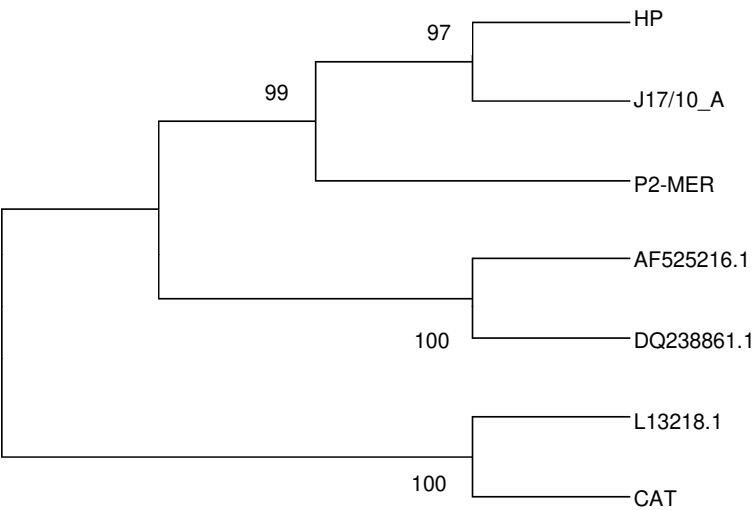

B

|            |   | 1     | 2     | 3     | 4     | 5     | 6     | 7  |
|------------|---|-------|-------|-------|-------|-------|-------|----|
| HP         | 1 |       | 4     | 11    | 41    | 50    | 24    | 39 |
| J17/10_A   | 2 | 99,51 |       | 15    | 44    | 52    | 26    | 41 |
| P2MER      | 3 | 98,65 | 98,16 |       | 39    | 50    | 28    | 43 |
| CAT        | 4 | 94,98 | 94,61 | 95,23 |       | 36    | 49    | 64 |
| L13218.1   | 5 | 93,88 | 93,64 | 93,88 | 95,59 |       | 56    | 71 |
| AF525216.1 | 6 | 97,06 | 96,82 | 96,57 | 94,00 | 93,15 |       | 17 |
| DQ238861.1 | 7 | 95,23 | 94,98 | 94,74 | 92,17 | 91,31 | 97,92 |    |

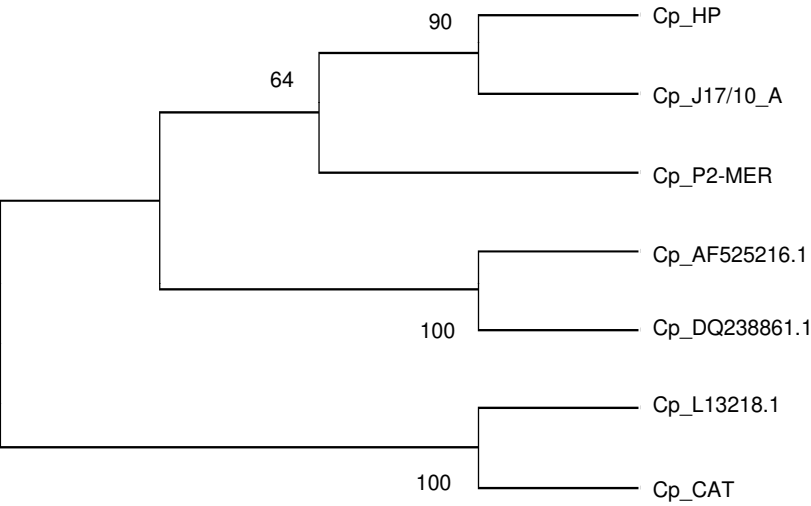

C

|            |   | 1      | 2     | 3     | 4     | 5     | 6     | 7  |
|------------|---|--------|-------|-------|-------|-------|-------|----|
| HP         | 1 |        | 0     | 2     | 26    | 24    | 11    | 19 |
| J17/10_A   | 2 | 100,00 |       | 2     | 26    | 24    | 11    | 19 |
| P2MER      | 3 | 99,78  | 99,78 |       | 26    | 22    | 12    | 20 |
| CAT        | 4 | 97,15  | 97,15 | 97,15 |       | 33    | 32    | 40 |
| L13218.1   | 5 | 97,37  | 97,37 | 97,59 | 96,38 |       | 24    | 32 |
| AF525216.1 | 6 | 98,79  | 98,79 | 98,68 | 96,49 | 97,37 |       | 8  |
| DQ238861.1 | 7 | 97,92  | 97,92 | 97,81 | 95,61 | 96,49 | 99,12 |    |

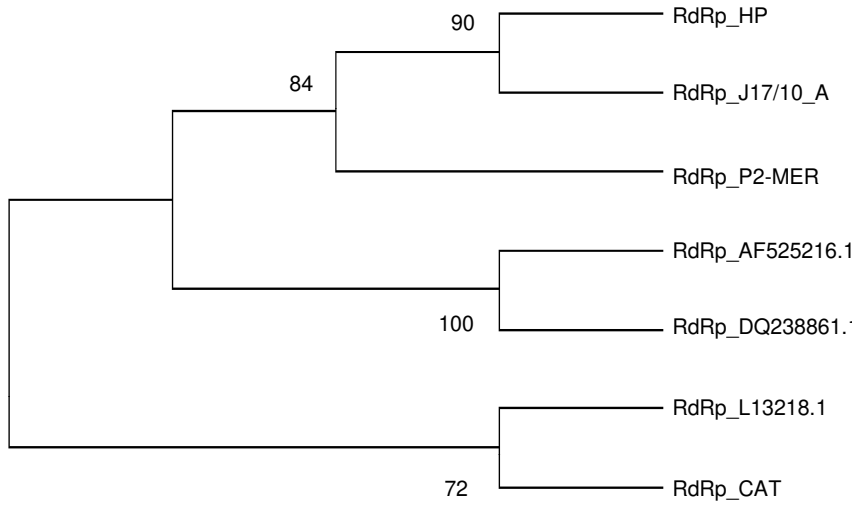

Supplement: Supplementary file 1 [file biomedicines-09-00654-s001.zip › Supplemental Files/Figure S2.pdf]
