# Supplementary material for: Re-Discovery of Giardiavirus: Genomic and Functional Analysis of Viruses from Giardia duodenalis Isolates
Source: Biomedicines. 2021 Jun 8;9(6):654. doi: 10.3390/biomedicines9060654 (PMC8230311; doi:10.3390/biomedicines9060654)
Supplement: Supplementary file 1 [file Figure S5.pdf › Supplemental Files/Figure S5.pdf]

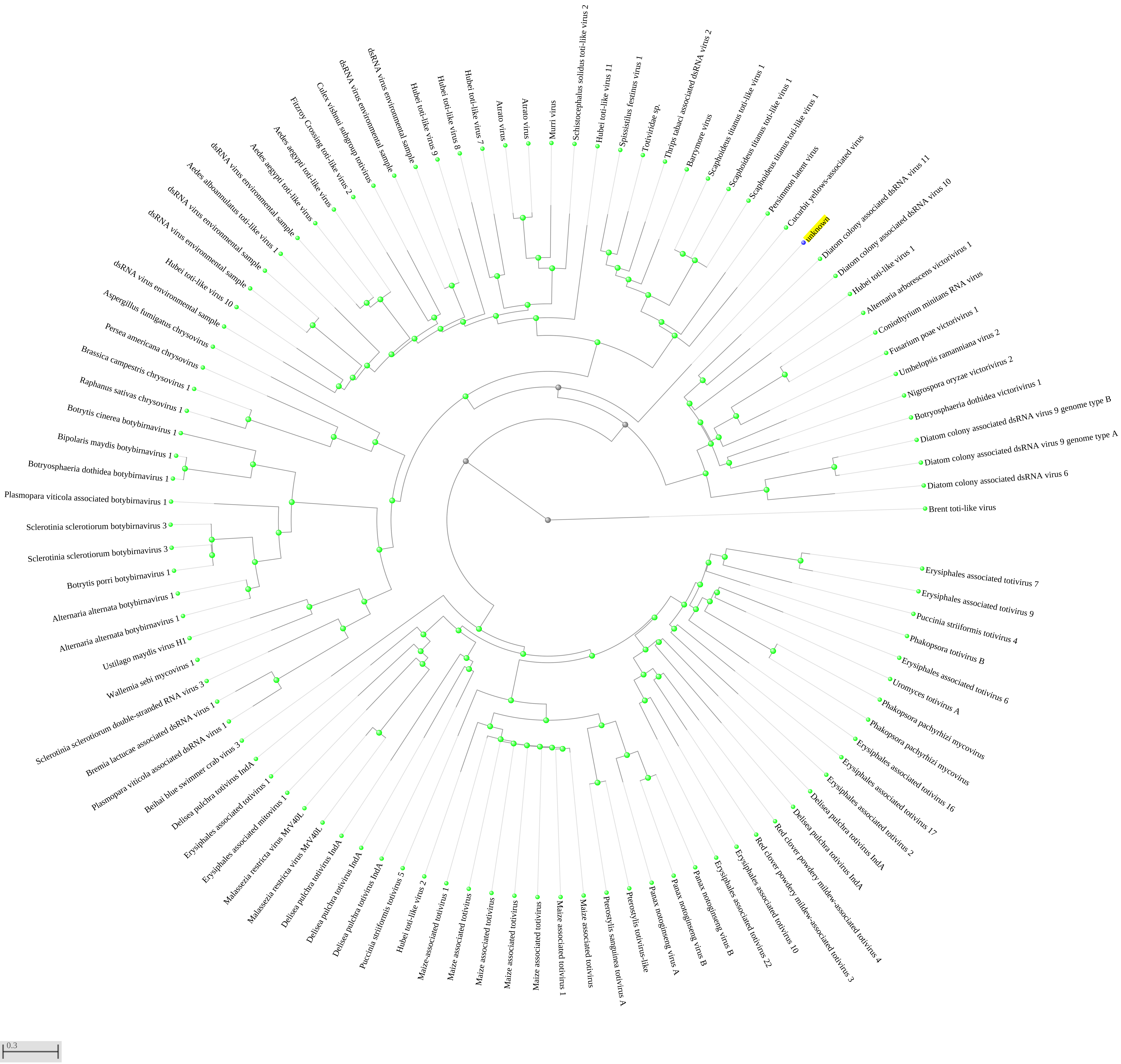
Brent toti-like virus
Diatom colony associated dsRNA virus 6
Diatom colony associated dsRNA virus 9 genome type A
Diatom colony associated dsRNA virus 9 genome type B
Botryosphaeria dothidea victorivirus 1
Nigrospora oryzae victorivirus 2
Umbelopsis ramanniana virus 2
Fusarium poae victorivirus 1
Coniothyrium minitans RNA virus
Alternaria arborescens victorivirus 1
Hubei toti-like virus 1
Diatom colony associated dsRNA virus 10
Diatom colony associated dsRNA virus 11
unknown
Cucurbit yellows-associated virus
Persimmon latent virus
Scaphoideus titanus toti-like virus 1
Scaphoideus titanus toti-like virus 1
Scaphoideus titanus toti-like virus 1
Barnymore virus
Thrips tabaci associated dsRNA virus 2
Totiviridae sp.
Spissistilus festinus virus 1
Hubei toti-like virus 11
Schistocephalus solidus toti-like virus 2
Murri virus
Atrato virus
Atrato virus
Hubei toti-like virus 7
Hubei toti-like virus 8
Hubei toti-like virus 9
dsRNA virus environmental sample
dsRNA virus environmental sample
Culex vishnui subgroup totivirus
Fitzroy Crossing toti-like virus 2
Aedes aegypti toti-like virus
Aedes aegypti toti-like virus
dsRNA virus environmental sample
Aedes alboannulatus toti-like virus 1
dsRNA virus environmental sample
dsRNA virus environmental sample
Hubei toti-like virus 10
dsRNA virus environmental sample
Aspergillus fumigatus chrysovirus
Persea americana chrysovirus
Brassica campestris chrysovirus 1
Raphanus sativas chrysovirus 1
Botrytis cinerea botybirnavirus 1
Bipolaris maydis botybirnavirus 1
Botryosphaeria dothidea botybirnavirus 1
Plasmopara viticola associated botybirnavirus 1
Sclerotinia sclerotiorum botybirnavirus 3
Sclerotinia sclerotiorum botybirnavirus 3
Botrytis porri botybirnavirus 1
Alternaria alternata botybirnavirus 1
Alternaria alternata botybirnavirus 1
Ustilago maydis virus H1
Wallemia sebi mycovirus 1
Sclerotinia sclerotiorum double-stranded RNA virus 3
Bremia lactucae associated dsRNA virus 1
Plasmopara viticola associated dsRNA virus 3
Beihai blue swimmer crab virus 3
Delisea pulchra totivirus IndA
Erysiphales associated totivirus 1
Erysiphales associated mitovirus 1
Malassezia restricta virus MrV40L
Malassezia restricta virus MrV40L
Delisea pulchra totivirus IndA
Delisea pulchra totivirus IndA
Puccinia striiformis totivirus 5
Hubei toti-like virus 2
Maize-associated totivirus 1
Maize associated totivirus
Maize associated totivirus
Maize associated totivirus
Maize associated totivirus
Maize associated totivirus 1
Maize associated totivirus
Pterostylis sanguinea totivirus A
Pterostylis totivirus-like
Panax notoginseng virus A
Panax notoginseng virus B
Panax notoginseng virus B
Erysiphales associated totivirus 22
Erysiphales associated totivirus 10
Red clover powdery mildew-associated totivirus 3
Red clover powdery mildew-associated totivirus 4
Delisea pulchra totivirus IndA
Delisea pulchra totivirus IndA
Delisea pulchra totivirus IndA
Erysiphales associated totivirus 2
Erysiphales associated totivirus 17
Erysiphales associated totivirus 16
Phakopsora pachyrhizi mycovirus
Phakopsora pachyrhizi mycovirus
Uromyces totivirus A
Erysiphales associated totivirus 6
Phakopsora totivirus B
Puccinia striiformis totivirus 4
Erysiphales associated totivirus 9
Erysiphales associated totivirus 7
0.3
